# International Union of Basic and Clinical Pharmacology. CXXI. Apelin receptor pharmacology in the human cardiovascular system and emerging clinical applications

**DOI:** 10.1016/j.pharmr.2026.100130

**Published:** 2026-03-14

**Authors:** Anthony P. Davenport, Thomas L. Williams, Duuamene Nyimanu, Robyn G.C. Macrae, Rhoda E. Kuc, Fiona A. Chapman, Peiran Yang, Neeraj Dhaun, Janet J. Maguire

**Affiliations:** 1 Experimental Medicine and Immunotherapeutics, University of Cambridge, Cambridge, United Kingdom; 2 The Jared Grantham Kidney Institute, University of Kansas Medical Center, Kansas City, Kansas; 3 BHF/University of Edinburgh Centre for Cardiovascular Sciences, Queen’s Medical Research Institute, Edinburgh, United Kingdom; 4 Department of Renal Medicine, Royal Infirmary of Edinburgh, Edinburgh, United Kingdom; 5 State Key Laboratory of Respiratory Health and Multimorbidity, Institute of Basic Medical Sciences, Chinese Academy of Medical Sciences, School of Basic Medicine, Peking Union Medical College, Beijing, China

## Abstract

The apelin receptor binds 2 families of endogenous peptide, apelin and Elabela, but unusually these share little sequence similarity in the N-terminal sequences of the binding domains. Cryo-electron microscopy, X-ray crystallography combined with AlphaFold has yielded a molecular map of the interaction of amino acids with the apelin receptor in complex with endogenous peptides and biased ligands. In the early embryo, the apelin signaling pathway is essential for cardiovascular development, with receptor knockout models displaying severe cardiovascular defects. In adults, the principal short-term effects of [Pyr^1^]apelin-13, infused into healthy volunteers was increased cardiac output and decreased peripheral resistance without side effects. Importantly, these beneficial effects of systemic apelin were retained in patients with heart failure and pulmonary arterial hypertension. In chronic kidney disease, [Pyr^1^]apelin-13 showed additional therapeutic potential, increasing glomerular filtration rate while reducing proteinuria. Identification of these favorable actions in disease has sparked the development of more effective agonists with improved pharmacokinetics and pharmacodynamics profiles. Among these are G protein-biased peptide agonists, designed to minimize receptor desensitization by reducing internalization via the *β*-arrestin pathway. These have shown efficacy in proof-of-concept studies and in animal models of pulmonary arterial hypertension, one of the most promising therapeutic targets. This review focuses on the clinical pharmacology of the apelin receptor, exploring the pathophysiology of diseases where the apelin signaling pathway is dysregulated that have emerged during the last 5 years.

**Significance Statement:**

This review focuses on the pharmacology of the apelin receptor where structural analysis has generated a molecular map of interaction with endogenous ligands, apelin and Elabela, as well as with peptide and small molecule agonists. Novel unbiased and biased apelin agonists are progressing through the clinic targeting pathophysiological conditions where the apelin signaling pathway is dysregulated.

## I Introduction

**Fig. 1:**
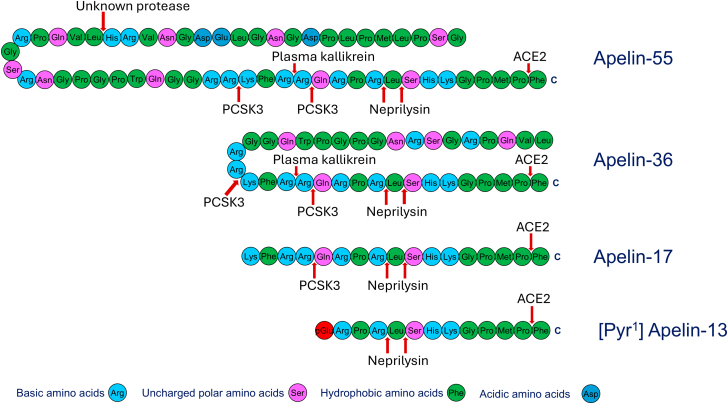
Amino acid sequences of the apelin family of peptides. The protease that cleaves apelin-55 (proapelin) to apelin-36 (mature apelin) is not yet known, but the furin, proprotein convertase subtilisin/kexin-3 (PCSK3) is known to cleave apelin-55 into apelin-17 and apelin-13. Plasma kallikrein cleaves apelin-17 into a 14-mer, apelin-14, at the N-terminus, and may also cleave the longer isoforms, apelin-36 and apelin-55. Apelin-13, undergoes further posttranslational modification to form [Pyr^1^]apelin-13 the most abundant isoform in humans. ACE2 cleaves all apelin isoforms at the C-terminus to remove the terminal phenylalanine residue. Neprilysin cleaves the RPRL motif and is the only protease that cleaves and completely inactivates apelin peptides. The physicochemical properties of the amino acid residues are as indicated by the color-coding.

**Fig. 2:**
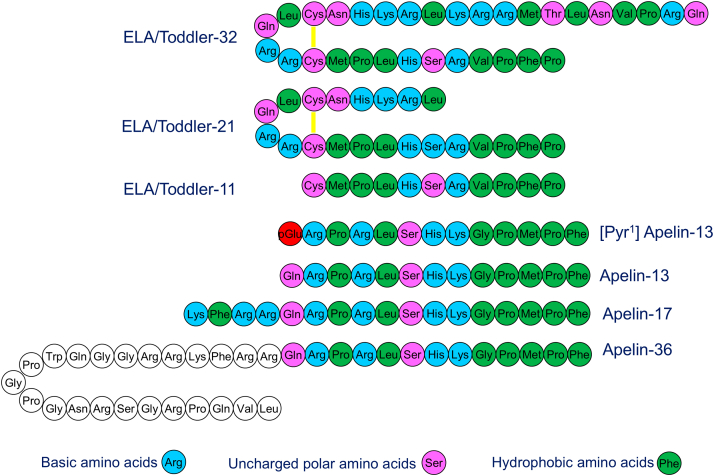
The amino acid sequence of ELA-32, ELA-21 and ELA-11 compared with [Pyr^1^]apelin-13, the principal isoform in humans together with the sequences of longer endogenous isoforms. Both apelin and have similar physicochemical properties (as indicated by the color-coding) and partially conserved C-terminus. The longer isoforms, ELA-32 and ELA-21 are predicted to forma disulfide bridge (yellow) between the cysteine residues.

The apelin receptor belongs to Family A of the G protein-coupled receptors (GPCRs) comprising nearly 200 transmembrane proteins that are targets for about a third of all clinically approved medicines. The receptor is unusual in binding 2 distinct families of peptide ligands, apelin and Elabela/Toddler (ELA). These are predicted to be synthesized from their respective preproproteins (apelin 55 and ELA 55) to generate shorter active fragments such as [Pyr^1^]apelin-13 (Fig. 1) and ELA-21 (Fig. 2).

In humans, the apelin receptor gene *APLNR* (located on chromosome 11q12.1) encodes a 380 amino acid protein, principally coupled to G protein alpha subunit (G_i_). The gene was originally identified as a potential GPCR and named *APJ*^1^ based on 30% sequence homology (with up to 54% in the transmembrane domains) with the angiotensin II type 1 receptor (AT_1_). The gene is also known as angiotensin II receptor-like 1 (*AGTRL1*) but does not bind angiotensin II (ANG II) and the receptor was classified as an “orphan GPCR” as the endogenous ligand(s) were not known. An endogenous ligand was identified by screening tissue extracts from bovine stomach that activated *AGTRL1* gene that was expressed in Chinese hamster ovary (CHO) cells and named apelin-36 apelin (APJ endogenous ligand, Tatemoto et al^2^). Further peptides were synthesized based on dibasic proteolytic cleavage sites in the 77 amino acid preproprotein encoded by apelin peptide gene (*APLN*) located on the X chromosome in humans and shown to have similar pharmacological actions: apelin-17,^3^ apelin-13,^4^ and pyroglutamated [Pyr^1^]apelin-13^5^ (Figs. 1 and 2).

The majority of orphan GPCRs in Class A have been paired with their cognate ligands by screening libraries of existing compounds with known physiological and/or pharmacological actions.^6^ The discovery of a novel peptide isolated from tissue extracts activating an orphan GPCR is unusual. It ignited the field to explore the role of this new signaling pathway. With the development of labeled agonists and selective antagonists, sufficient data had accumulated for the International Union of Basic and Clinical Pharmacology to recommend in 2010 the receptor protein be classified as the apelin receptor.^7^ Since then, there has been a large body of work studying the relationship between the ligand and receptor, as well as their physiological and pathophysiological roles in a number of diseases.

In 2013, the field was surprised by the discovery of a second endogenous apelin receptor ligand by 2 independent groups. Chng et al^8^ reported first, naming the peptide Elabela (Epiboly LAte Because Endoderm LAte), which was the first observable developmental phenotype when the gene was deleted in zebrafish (*Danio rerio*) embryos. Pauli et al^9^ named the peptide “Toddler” after absence or overproduction of the peptide was observed to reduce motility of mesendodermal cells during zebrafish gastrulation. These authors proposed ELA was a 54 amino acid prepropeptide that was cleaved to produce a 32 amino acid mature secreted peptide (ELA-32, Table 1).^8^, ^9^, ^10^, ^11^, ^12^, ^13^, ^14^, ^15^, ^16^, ^17^, ^18^, ^19^, ^20^, ^21^, ^22^, ^23^, ^24^, ^25^, ^26^, ^27^, ^28^, ^29^, ^30^, ^31^, ^32^, ^33^, ^34^, ^35^, ^36^, ^37^, ^38^, ^39^, ^40^, ^41^, ^42^, ^43^, ^44^, ^45^, ^46^, ^47^, ^48^, ^49^, ^50^ As with apelin, ELA is also proposed to undergo proteolytic cleavage, generating smaller active isoforms (ELA-22, ELA-21, and ELA-11), which surprisingly share little sequence similarity (∼25%) with apelin^8^^,^^9^^,^^51^^,^^52^ although there is some similarity in the location of hydrophobic residues. In 2019 there was sufficient pharmacological data to recommend that ELA was a second endogenous ligand for the apelin receptor, although it shows little sequence homology to apelin. In this review, the peptide will be referred to using the abbreviation ELA for Elabela, following the convention of recommending naming the peptide according to the precedence of discovery.^53^ The gene encoding ELA-54 was originally identified in the genomes of fish and humans but although highly conserved (including in rodents) it was originally misclassified as a noncoding region. The HUGO Gene Nomenclature Committee recommended nomenclature in humans is *APELA* (apelin receptor early endogenous ligand), which is located on chromosome 4.

**Table 1:** Pharmacological parameters for apelin and ELA endogenous peptides, synthetic peptides, small molecule agonists, biased agonists, and antagonists

1	Action	Value	Parameter	Reference
Human endogenous apelin peptides				
Apelin-13	Full agonist	8.8, 9.5	pIC_50_	Hosoya et al,^10^ Fan et al,^11^ Medhurst et al^12^
[Pyr^1^]apelin-13	Full agonist	8.9	pIC_50_	Medhurst et al^12^
Apelin-17	Agonist	9.0	pIC_50_	Medhurst et al^12^
Apelin-36	Full agonist	8.6	pIC_50_	Hosoya et al,^10^ Fan et al,^11^ Medhurst et al^12^
Proposed endogenous human ELA peptides				
Elabela/Toddler-11 (ELA-11)	Agonist	7.2	pIC_50_	Yang et al^13^
Elabela/Toddler-21 (ELA-21)	Agonist	8.7	pIC_50_	Yang et al^13^
Elabela/Toddler-32 (ELA-32)	Agonist	8.7	pIC_50_	Yang et al^13^
Fluorescently labeled apelin peptide analogs				
Apelin488	Agonist	7.29	pK_i_	Williams et al^14^
Apelin647	Agonist	8.92	pK_i_	Williams et al^14^
Fluorescently labeled ELA peptide analogs				
ELA488	Agonist	6.96	pK_i_	Williams et al^14^
ELA647	Agonist	6.57	pK_i_	Williams et al^14^
Radiolabeled apelin peptide analogs				
[^3^H](Pyr^1^)[Met(0)11]-apelin-13	Full agonist	8.6	pK_d_	Medhurst et al^12^
[^125^I]apelin-13	Full agonist	9.2	pK_d_	Fan et al^11^
[^125^I](Pyr^1^)apelin-13	Full agonist	9.5	pK_d_	Katugampola et al^15^
[^125^I][Glp^65^Nle^75^,Tyr^77^]apelin-13	Full agonist	10.7	pK_d_	Hosoya et al^10^
[^125^I][Nle^75^,Tyr^77^]apelin-36	Full agonist	11.2	p*K*_d_	
Apelin agonists: peptide analogs				
Apelin-12 analogs				
Cyclo apelin-12 (1–12)	Full agonist	6.3	pEC_50_	Hamada et al^16^
Cyclourea apelin-12 (1–7)	Full agonist	6.8	pEC_50_	Hamada et al^16^
Cyclo apelin-12 (7–12)	Full agonist	7.1	pEC_50_	Hamada et al^16^
Palmitate-VTLPLWATYTYR (compound 1)	Full agonist	7.5	pEC_50_	McKeown et al^17^
Apelin-13 analogs				
Apelin-13 (F13A)	Partial agonist	9.54	pD_2_	Yang et al^13^
PyrRPRLSHKGPNle-Aia-F-OH (compound 47)	Agonist	9.75	pK_i_	Tran et al^18^
PyrRPRLSHKGPNle1-Nal-Dbzg-OH (compound 53)	Agonist	9.75	pK_i_	Tran et al^18^
Apelin-17 analogs				
LIT01-196	Agonist	9.1	pK_i_	Flahault et al^,^^19^^,^^20^
hArg17A2	Agonist	9.08	pK_i_	Fernandez et al^21^
hArgNMeCha17A2	Agonist	9.19	pK_i_	Fernandez et al^21^
AlbudAb-MM202 AlbudAb-sSMCC-(PEG)_4_-QRPRLSHKGP-Nle-P-(3,4,5 trifluoro)F	Full agonist	9.4	pK_i_	Read et al^22^
		9.52	pD_2_	
JN241-9 (single-domain antibody)	Full agonist	7.45	pD_2_	Ma et al^23^
ELA agonists: peptide analogs				
Alkaline phosphatase (AP)-tagged ELA-32 (AP-apela)	Agonist	9.3	pKd	Deng et al^24^
Pal-E11 Pal-*γ*Glu-CMPLHSRVPFP-amide	Agonist	9.66	pK_i_	Wang et al^25^
Biased apelin peptide agonists				
MM07 H-C(1)RPRLC(1)HKGPMPF-OH	Biased agonist	9.5	pEC_50_	Brame et al^26^
H2N-c[X-R-L-S-X]-K-G-P-(D-1Nal) (compound 39)	Biased agonist	9.2	pK_i_	Tran et al^27^
H2N-c[X-R-L-S-X]-K-G-P-(D-2Nal) (compound 40)	Biased agonist	8.2	pK_i_	Tran et al^27^
NXE’065	Biased agonist	7.0	pK_i_	Williams et al^28^
Unbiased agonists: small molecules				
E339-3D6 [9-[4-[12-[[6-[[3-(1-benzyl-3-methylimidazol-1-ium-4-yl)-1-[(1-methylpiperidin-1-ium-4-yl)amino]-1-oxopropan-2-yl]amino]-5-[[2-(2-imino-3-methyl-3H-1,3-thiazol-3-ium-4-yl)acetyl]amino]-6-oxohexyl]carbamoylamino]dodecylsulfamoyl]-2-sulfophenyl]-6-(diethylamino)xanthen-3-ylidene]-diethylazanium;2,2,2-trifluoroacetate	Agonist	6.4	pK_i_	Iturrioz et al^29^
ML233 [(Z)-5-Cyclohexyl-2-methyl-4-oxocyclohexa-2,5-dien-1-ylidene]amino benzenesulfonate)	Full agonist	5.4	pEC_50_	Khan et al^30^
Compound 13 (S)-N-(1-(Cyclobutylamino)-1-oxo-5-(piperidin-1-yl)pentan-3-yl)-1-cyclopentyl-5-(2,6-dimethoxyphenyl)-1H-pyrazole-3-carboxamide	Agonist	7.05	pKi	Narayanan et al^31^
Compound 47 (S)-1-Cyclopentyl-N-(4-(3,3-difluoropiperidin-1-yl)-1-(1H-tetrazol-5-yl)butan-2-yl)-5-(2-(trifluoromethyl)phenyl)-1H-pyrazole-3-carboxamide	Agonist	8.2	pEC_50_	Narayanan et al^32^
BMS-986224 3-[5-[(5-Chloropyridin-2-yl)methyl]-1,3,4-oxadiazol-2-yl]-5-(2,6-dimethoxyphenyl)-6-(ethoxymethyl)-4-hydroxy-1H-pyridin-2-one	Agonist	10.3	pK_i_	Myers et al,^33^ Gargalovic et al^34^
Compound 14a (R)-2-Butyl-5-(3-(5-chloropyridin-2-yl)pyrrolidine-1-carbonyl)-1-(2,6-dimethoxyphenyl)-6-hydroxypyrimidin-4(1H)-one	Agonist	10.6	pEC_50_	Pi et al^35^
Compound 14 3-(5-((5-Chloropyridin-2-yl)methyl)-1,3,4-oxadiazol-2-yl)-5-(2,6-dimethoxyphenyl)-6-(ethoxymethyl)-4-hydroxypyridin-2(1H)-one	Agonist	10.1	pKi	Johnson et al^36^
Compound 21 (R)-5-(3-(5-Chloro-3-fluoropyridin-2-yl)pyrrolidine-1-carbonyl)-1-(2,6-diethylphenyl)-6-hydroxy-2-(1-methyl-1H-pyrazol-3-yl)-pyrimidin-4(1H)-one	Agonist	10.2	pEC_50_	Meng et al^37^
Compound 15a (S)-2′-(1H-1,3-Benzodiazol-2-yl)-6′-chloro-4-{[(1R)-1-phenylbutyl]carbamoyl}-[1,1′-biphenyl]-2-carboxylic acid	Agonist	10.0	pEC_50_	Su et al^38^
AM-8123 (1S,2S)-N-(4-(2,6-Dimethoxyphenyl)-5-(5-methylpyridin-3-yl)-4H-1,2,4-triazol-3-yl)-1-isopropoxy-1-(5-methylpyrimidin-2-yl)propane-2-sulfonamide	Agonist	9.4	pEC_50_	Ason et al^39^
APT-101 ((S)-3-(1-Cyclopentyl-5-(2-(trifluoromethyl)phenyl)-1H-pyrazole-3-carboxamido)-5-(3,3-difluoropiperidin-1-yl)pentanoic acid hydrochloride)				
Cmpd644 ((1S,2R)-N-(4-(2,6-dimethoxyphenyl)-5-(6-methylpyridin-2-yl)-4H-1,2,4-triazol-3-yl)-1-hydroxy-1-(5-methylpyrimidin-2-yl) propane-2-sulfonamide	Agonist			Yue et al^40^
G protein biased apelin small molecule agonists				
CMF-019 (3S)-5-methyl-3-[[1-pentan-3-yl-2-(thiophen-2-ylmethyl)benzimidazole-5-carbonyl]amino]hexanoic acid	Biased agonist	8.58	pK_i_	Read et al^41^
		10.0	pEC_50_	
WN353	Biased agonists			Wang et al^42^
WN561				
AP-7 and AP-16	Biased agonists			Sun et al^43^
Antagonists: peptides				
ALX40-4C Ac-(D-Arg)9-NH2	Antagonist	5.5	pIC_50_	Zhou et al^44^
Protamine	Antagonist			Le Gonidec et al^45^
MM54 cyclo(1-6)CRPRLC-KH-cyclo(9-14)CRPRLC	Antagonist	8.2	pK_i_	Macaluso et al^46^
MM193 CRPRNleCKHCRAibRNle (3,4,5-trifluoro-)F	Antagonist	7.7	pK_i_	Davenport et al^47^
MM315 Myristoyl-ARPRNleAbuKHAbuRAibRNle(3,4,5-trifluoro-)F	Antagonist	8.5	pK_i_	Davenport et al^47^
Antagonists: small molecule				
ML221 [4-oxo-6-(pyrimidin-2-ylsulfanylmethyl)pyran-3-yl] 4-nitrobenzoate	Antagonist	5.8	pIC_50_	Maloney et al^48^
Amodiaquine 4-[(7-chloroquinolin-4-yl)amino]-2-(diethylaminomethyl)phenol	Noncompetitive antagonist			McAnally et al^49^
Investigational clinical G protein biased peptide agonist				
ANPA-0073 (structure not published)	Biased agonist	8.3 cAMP	pEC_50_	Shi et al^50^
		7.5 *β*-Arrestin		
Investigational clinical small molecule agonist				
Azelaprag (AMG 986; BGE-105) (2S,3R)-N-[4-(2,6-dimethoxyphenyl)-5-(5-methylpyridin-3-yl)-1,2,4-triazol-3-yl]-3-(5-methylpyrimidin-2-yl)butane-2-sulfonamide	Agonist	9.5	pEC_50_	Ason et al^39^

The aim of this review is to focus on progress in apelin receptor pharmacology mainly in humans since the last Pharmacological Review,^53^ particularly on new insights gained from structural biology in ligand receptor interactions using X-ray crystallography, cryo-electron microscopy (cryo-EM) and AlphaFold. The field has been catalyzed by the emergence of new and unexpected clinical indications (Table 2),^51^^,^^53^, ^54^, ^55^, ^56^, ^57^, ^58^, ^59^, ^60^, ^61^, ^62^, ^63^, ^64^, ^65^, ^66^, ^67^, ^68^, ^69^, ^70^, ^71^, ^72^, ^73^, ^74^, ^75^, ^76^ such a beneficially improving glomerular filtration while reducing proteinuria in chronic kidney disease (CKD) and slowing muscle wasting.

**Table 2:** Investigational studies on the effects of [Pyr^1^]apelin on forearm blood flow or following systematic infusion and clinical trials of small molecule or peptide agonists

National Clinical Trial Number (Clinical Trial Name) ClinicalTrials.gov Study URL	Clinical Study Title	Compound/Route of Administration	Subjects	Results	Reference
Cardiovascular and cardiopulmonary interventional studies					
	Vascular effects of apelin in vivo in man	[Pyr^1^]apelin-13 or apelin-36 0.1–30 mol/min Intrabrachial FBF	Healthy volunteers	Nitric oxide dependent peripheral vasodilatation	Japp et al,^54^ Barnes et al,^55^ Japp and Newbyl^56^
		[Pyr^1^]apelin-13 Intrabrachial, FBF Systemic Apelin-36 Intracoronary bolus	Healthy volunteers and HF	Nitric oxide dependent peripheral vasodilation Increased cardiac index, lowered mean arterial pressure, peripheral vascular resistance Vasodilation Increased cardiac output	Japp et al^57^^,^^58^
NCT00901719 NCT00901888 NCT01049646 NCT01179061	Investigating the inotropic potential of apelin	[Pyr^1^]apelin-13 Intrabrachial, FBF 0.3–3.0 nmol/min Systemic 30–300 nmol/min Prolonged systemic 30 nmol/min 6 h	Healthy volunteers and HF	Beneficial cardiovascular actions preserved in the presence of renin-angiotensin system activation	Barnes et al^59^
NCT02150694 (HEAP)	Local hemodynamic effects of apelin agonists and antagonists in man in vivo	[Pyr^1^]apelin-13 1–100 nmol/min Intrabrachial, FBF	Healthy volunteers	Dose-dependent increase in forearm blood flow	Brame et al^26^
NCT02150694 (HEAP)	Local hemodynamic effects of apelin agonists and antagonists in man in vivo	1–100 nmol/min [Pyr^1^] Apelin-13_(1-12)_ Intrabrachial FBF	Healthy volunteers	Dose-dependent increase in forearm blood flow	Yang et al^60^
NCT02129309 (ALPHA)	Hemodynamic effects of apelin agonists and antagonists in man in Chronic obstructive pulmonary disease (COPD) with raised pulmonary artery pressures	1–100 nmol/min [Pyr1] Apelin Intrabrachial FBF Systemic	COPD with pulmonary hypertenison	Significant increase in FBF, peripheral vasodilatation, cardiac output	Brame et al^61^
NCT01590108	The study of apelin-APJ system on patients with pulmonary hypertension and healthy subjects	Ascending doses for 5 min of 10, 30, and 100 nmol/min Intravenous during invasive right heart catheterization	Idiopathic pulmonary arterial hypertension	Reduction in pulmonary vascular resistance and increase in cardiac output Potentiated by PDE5 inhibition	Brash et al^62^
Metabolic interventional studies					
NCT02724566	Effect of apelin on insulin sensitivity in type 2 diabetic volunteers	[Pyr^1^]apelin-13 2 h infusion 30 nmol/kg Systemic	Diabetes	Increased insulin sensitivity. After 2 h infusion increased glucose uptake of skeletal muscles and adipose tissue in overweight humans.	Gourdy et al^63^
	Beneficial effects of apelin on vascular function in patients with central obesity	[Pyr^1^]apelin-13 1.5 nmol/min Intrabrachial FBF	Centrally obese patients	Increased insulin stimulated vasodilation reduced Ang II and ET-1-dependant vasocontraction	Schinzari et al^64^
NCT03449251 (Define)	A series of pilot studies to evaluate the hemodynamic and metabolic effects of apelin and relaxin	[Pyr^1^]apelin-13 30 nmol/min for 2 h	Increased body mass index and type 2 diabetes	Increased cardiac and stroke volume index, reduced peripheral vascular resistance	Sulentic et al^65^
Renal interventional studies					
NCT03956576	Apelin as a potential treatment for chronic kidney disease	1 nmol/min and 30 nmol/min Systemic	Chronic kidney disease and healthy volunteers	Beneficial significant reduced glomerular filtration rate, effective filtration fraction, and proteinuria in CKD	Chapman et al^66^
NCT06277336 (ESCAPE)	Effects of intravenous [Pyr^1^]apelin-13 on healthy volunteers with artificially induced SIAD	[Pyr^1^]Apelin-13 180-mL infusion over 3 h at 1 or 10 nmol/min	SIAD	In progress	Christ-Crain et al, in progress
Peptide analogs					
NCT02696967 (phase 2)	A study of CLR325 in patients with chronic stable heart failure	Cyclic peptide agonist apelin analog 0.25, 2.5, and 8 mg/kg/min Intravenous	Chronic stable heart failure	CLR325 safe and well tolerated for the treatment of patients with chronic stable heart failure through the monitoring of relevant clinical and laboratory safety parameters	Novartis^67^
Small molecule agonist AMG 986; BGE-105, azelaprag (clinical studies discontinued, raised liver enzymes)					
NCT03276728 (phase 1B)	Study to evaluate the safety and tolerability of AMG 986 in healthy volunteers and patients with heart failure	Oral/IV AMG 986 or placebo (part A); multiple-dose oral/IV AMG 986 or placebo (part B); or escalating-dose oral AMG 986 or placebo (part C)	Healthy volunteers and patients with heart failure	AMG 986 treatment was well tolerated but lack of efficacy. No clinically meaningful pharmacodynamic effects in patients with HF	Winkle et al^68^
NCT03318809 (phase 1)	Study to evaluate the safety, tolerability, and pharmacokinetics of AMG 986 administered orally to healthy volunteers and participants with severely impaired renal function	AMG 986 200 mg Oral	Normal renal function vs renal impairment	C_max_ and AUC similar in both group and AMG 986 had acceptable safety profile	Trivedi et al^69^, ^70^, ^71^, ^72^
NCT06141889 (phase 1) https://clinicaltrials.gov/study/NCT06141889	Pharmacokinetics study of azelaprag (BGE-105) in older adult healthy volunteers	Azelaprag (AMG 986; BGE-105) Single dose, open label, cross over, and multiple dose	Healthy volunteers	No results posted	
NCT06515418 (STRIDES, phase 2) https://clinicaltrials.gov/study/NCT06515418	Efficacy and safety of oral azelaprag plus once weekly tirzepatide compared with tirzepatide alone in participants with obesity aged ≥55 y	Azelaprag 300 mg daily Complicated dosing Tirzepatide 5 mg once weekly sc	≥55 y with obesity	Terminated, liver transaminitis without clinically significant symptoms in some subjects receiving azelaprag	Bioage^73^^,^^74^
Small molecule G protein biased agonist					
ACTRN12621000644864 ANPA-0073 (phase 1)	A first-in-human single/multiple ascending dose study of ANPA-0073, a novel small molecule G-protein biased apelin receptor agonist, in healthy volunteers	Single daily ascending oral doses 2–600 mg Multiple daily ascending oral doses 75–500 mg, 7 days ANPA-0073	Healthy volunteers	ANPA-0073 generally well tolerated, maximal dose of 600 mg recommended	Bach et al^75^
Investigational peptide antagonist MM54					
NCT02129309 (ALPHA)	Hemodynamic effects of apelin agonists and antagonists in man in COPD with raised pulmonary artery pressures	MM54	Healthy volunteers	Arterial and venous vasodilation	Davenport et al^76^

FBF, forearm blood flow; PDE5, phosphodiesterase-5.

The following reviews should be consulted for more details of the role of apelin in areas not covered by this article. Marsault et al^77^ provides a wide-ranging overview of the apelinergic system. The review by de Oliveira et al^78^ is recommended for comprehensive information and schematics on the dysregulated downstream pathways in cardiovascular, renal, and metabolic diseases and modulation by agonists. Wagenaar and Moll^79^ should be consulted for comprehensive information on the pharmacology of apelin compounds and their action in animal models. Couvineau and Llorens-Cortes^80^ review apelin receptor distribution and pharmacology focusing on water balance and cardiovascular function in rodents. Gao and Chen^81^ summarized the emerging actions of actions ELA (as well as apelin) in animal models.

The physiology and role in cardiovascular disease of apelin and ELA, the effect of deleting genes encoding these peptides in mice, as well as the action of modified apelin analogs in the cardiovascular system has been reviewed by Zhong.^82^ Interactions between apelin and renin-angiotensin-aldosterone system (RAAS) are reviewed by Chatterjee et al^83^ and Wagenaar and Moll.^84^

The role of apelin signaling in cancer is complex: Naldi et al^85^ review the role of apelin in individual cancers and modulation by apelin compounds as well as microRNAs in detail. Chen et al^86^ focus on the tumor microenvironment and interaction of apelin signaling with current cancer therapies. Jafarzadeh et al^87^ highlight the reciprocal interaction between apelin and noncoding RNAs, rapidly expanding new targets for therapeutic interventions. The following are recommended for further information on specific conditions: ischemic stroke,^88^ diseases of the central nervous system (CNS),^89^, ^90^, ^91^, ^92^, ^93^ Alzheimer’s,^94^ neuroprotection,^95^ cerebral ischemia,^93^^,^^96^ ischemic brain injury^97^; and obesity.^98^, ^99^, ^100^ Downstream receptor signaling has been reviewed by Wang et al,^101^ Zhang et al,^102^ Seo et al,^103^ Dagamajalu et al,^104^ and Murali and Aradhyam.^105^

## II Apelin receptor structure

### A X-ray crystallography and cryo-electron microscopy

To better understand the structural organization of the apelin receptor, a number of studies have successfully modeled or resolved the receptor protein, docked or bound to endogenous or exogenous ligands.

Initially, a 3-dimensional homology model of the human apelin receptor was built using a cholecystokinin receptor-1 model as a template.^106^ Docking apelin-17 into the binding site of this model allowed the investigators to visualize a hydrophobic cavity at the bottom of the binding pocket in which the C-terminal F17 of the peptide was embedded by W152 in transmembrane domain (TMD)4 and F255 and W259 of TMD6. Further molecular modeling and site-directed mutagenesis showed that the F255 and W259 residues were important in triggering receptor internalization, rather than directly playing a role in apelin binding or G protein activation.

These findings were supported in a subsequent study that used the validated cholecystokinin receptor-1 model, along with X-ray structures of the *β*2 and C-X-C chemokine receptor type 4 (CXCR4) receptors as templates.^107^ Docking of [Pyr^1^]apelin-13 into these models revealed the conservation of the hydrophobic cavity at the bottom of the binding site, in which the C-terminal F13 of the peptide was shown to be embedded. There were discrepancies at the top of the binding site between the models, but site-directed mutagenesis of the rat apelin receptor pointed to acidic residues D92, E172, and D282 as key mediators of apelin binding through interaction with the basic residues K8, R2, and R4 of the peptide, respectively.

Another homology model, based on the 2.5 Å resolution crystal structure of the human CXCR4 receptor, was used to investigate and compare the binding poses of apelin-13 and MM07, a G protein-biased cyclic peptide analog.^26^ The findings highlighted that sufficient volume was available in the binding site for the cyclized MM07 peptide to occupy a similar binding pose to apelin-13. This model was used subsequently to computationally dock a G protein-biased small molecule agonist, CMF-019.^41^ The key interactions identified between CMF-019 and the receptor were *π*-stacking (thiophene to Y88) and an ionic bond (carboxylate to R168), and an overlay with apelin-13 positioned CMF-019 approximately at the SHK region of the peptide. Finally, this model has also been used to investigate the structural alignment of apelin-13 versus ELA-11.^13^ Overall, apelin-13 and ELA-11 showed a high degree of overlap in the binding site, with both endogenous peptides sharing common hydrophobic binding derived from the presence of C-terminal hydrophobic moieties embedding into the complementary hydrophobic pocket of the receptor. It was hypothesized that F10 of ELA-11 would assume a pose corresponding with that of F13 of apelin-13, and the model indicated that the binding cavity of the receptor is large enough to accept the P11 of ELA-11 that extends beyond this point.

**Fig. 3:**
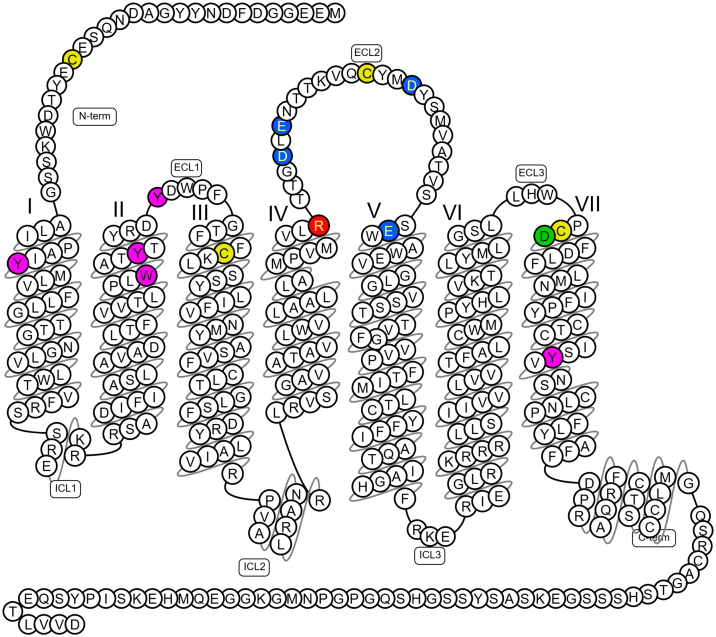
Snake plot summarizing the predicted key molecular interactions in the X-ray crystal structure of the 380 amino acid human apelin receptor in complex with the conformationally restrained 17-amino acid apelin peptide mimetic, AMG3054^108^ (see section X-ray crystallography and cryo-electron microscopy for further description). Key to amino acids: Blue: groove 1 (D172 ^ECL2^, E174 ^ECL2^, D184 ^ECL2^ and E194^5.31^). Green: groove 2 (D282^7.26^). Yellow: disulfide bonds between cysteine residues (C19^N-terminus^ - C281^7.25^ and C102^3.25^ - C181 ^ECL2^). Pink: hydrophobic cavity of binding pocket (Y35^1.39^, W85^2.60^, Y88^2.63^ Y93 ^ECL1^ and Y299^7.43^). Red: R168^4.64^ interacts with both P16 and C-terminal F (4-Cl-Phe17) residues in AMG3054. Image generated from data in GPCRdb,^109^ with superscript showing the Ballesteros–Weinstein numbering system for 7 transmembrane helices (1-7) and amino acids.^110^ ECL, extracellular loop; ICL, intracellular loop.

The first reported crystal structure of the apelin receptor was resolved at 2.6 Å in complex with a conformationally restrained 17-amino acid apelin peptide mimetic, AMG3054.^108^ The structure (Fig. 3)^109^^,^^110^ revealed that the peptide agonist adopts a lactam constrained curved 2-site binding pose. The first site is made up of groove 1 (D172, E174, and D184 in ECL2 and E194 in TM5) and groove 2 (D282 and surrounding residues), whilst the second site is formed of the N-terminal loop that is conformationally restricted by a disulfide bond between C19 and C281. A second disulfide bridge was identified between C102 and C181. The C-terminal end of AMG3054 binds deeply into the canonical binding pocket that lies approximately perpendicular to the membrane plane, with the C-terminal F (4-Cl-Phe17) interacting with a hydrophobic cavity comprising Y35, W85, Y88, Y93, and Y299 that also line site 1. P16 and 4-Cl-Phe17 of the AMG3054 ligand both interact with R168. The lactam ring formed by the sidechains of E10 and K13 in AMG3054 induce a perpendicular kink in the peptide that allows the N-terminus to slide into site 2 at the extracellular surface of the receptor, in parallel with the membrane plane. Interestingly, the authors demonstrated that 17-amino acid dynorphin A peptide binds to the *κ* opioid receptor via a similar 2-site binding mode, indicating this may be common among peptide-binding GPCRs.

Alanine scanning mutagenesis of the sites in groove 1 showed no impact on binding of apelin-13. It was concluded that shorter apelin isoforms may not interact with the acidic amino acids in groove 1 whilst longer isoforms such as apelin-36, which contain many positively charged amino acids, may. This could provide an explanation for longer lasting actions of apelin-36, although this was not experimentally determined. Further mutagenesis showed that many of the amino acids (eg, Y35, W85, and R168) in sites 1 and 2 critical for binding of AMG3054, were also critical for [^125^I]-apelin-13 radioligand binding and G protein signaling.

After this, another crystal structure of the apelin receptor in complex with a single domain antibody antagonist, JN241, was resolved at 3.2 Å.^23^ JN241 was shown to adopt a “wine stopper” shape, with all 3 complementarity-determining region (CDR) loops making extensive contacts on the extracellular side of the receptor, and with CDR3 deeply inserted into the orthosteric site. Residue E104 of JN241 was shown to be well accommodated in the positively charged apelin receptor binding pocket, forming electrostatic and hydrogen bonds with R168 and Y264. Comparison with AMG3054 revealed that JN421 did not make interactions with the hydrophobic pocket of the receptor, and, remarkably, the introduction of a hydrophobic Y residue between E104 and S105 of the antibody switched JN241 into a full apelin receptor agonist, JN241-9.

The first crystal structure of the apelin receptor in complex with a small molecule ligand, the agonist cmpd644, was resolved at 2.7 Å.^40^ Superimposition showed that the receptor adopted a conformation that was nearly identical to the inactive AMG3054 apelin receptor cocrystal structure described above. Unlike the 2-site binding mode observed for AMG3054, cmpd644 occupied site 1 of the pocket aligning with the C-terminal portion of the peptide, although cmpd644 notably inserted much deeper into site 1 than AMG3054. The cmpd644 cocrystal structure revealed 2 major conformational changes related to receptor activation; a movement of Y299 inducing the inward movement of TMD7, and a rotamer switch of W85 on activation.

**Fig. 4:**
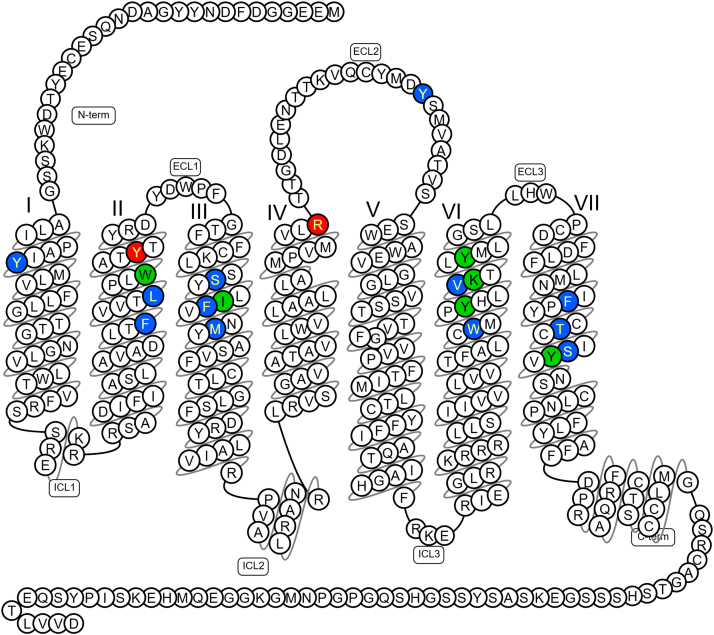
Snake plot of the X-ray crystal structure of the human apelin receptor in complex with the small molecule biased agonist CMF-019 summarizing the predicted key molecular interactions (see section X-Ray Crystallography and Cryo-Electron Microscopy, and Williams et al^28^). Key to amino acids: Green: both apelin 13 and CMF-019 interact with the following amino acids: (6) (W85^2.60^, I109^3.32^, Y264^6.51^, K268^6.55^, Y271^6.58^, Y299^7.43^). Red: apelin 13 only (2) (R168^4.64^, Y88^2.63^). Blue: CMF-019 only (12) (Y35^1.39^, L82^2.57^, F78^2.53^, S105^3.29^, F110^3.33^, M113^3.36^, Y185^ECL2^, V267^6.54^, W261^6.48^, F291^7.35^, T295^7.39^, S298^7.42^). C-terminus Pro^12^ and Phe^13^ of apelin-13 both interact with Arg (R168^4.64^) but R168H R/H168^4.64^ (red) variant results in loss of function. In contrast, the carbonyl amide of CMF-019 makes only a minor single interaction with R168^4.64^ and still able to activate/rescue R168H R/H168^4.64^ variant via other amino acids not shared with the endogenous ligand. Image generated from data in GPCRdb,^109^ with superscript showing the Ballesteros–Weinstein numbering system for 7 transmembrane helices (1-7) and amino acids.^110^ ECL, extracellular loop; ICL, intracellular loop.

Separately, an X-ray crystal structure of the apelin receptor was resolved in complex with the small molecule G protein-biased agonist, CMF-019.^28^ Overall, this structure adopted an intermediate conformation, reminiscent of an active state in the absence of full activation by G protein binding (Fig. 4).^28^^,^^109^^,^^110^ The elongated CMF-019 molecule was shown to bind in a semivertical pose, extending from the upper part of the large orthosteric pocket, making mostly Van der Waals contacts with the receptor. Comparisons with the cmpd644 structure showed that, despite CMF-019 and cmpd644 being of different chemotypes, they generally share overlapping binding sites and exhibit very similar binding modes, with hydrophobic moieties binding to the same lipophilic subpockets. Comparisons with apelin and ELA peptide bound structures showed that the CMF-019 binding sites overlap in part with those for C-terminal residues of the endogenous peptides, but the small molecule reaches deeper down into the orthosteric pocket. Of note in this structure, the R168 receptor residue formed only a minor direct interaction with the core of CMF-019, engaging mostly indirectly via a salt bridge with an oleic acid molecule that was observed to protrude into the binding site. Additionally, CMF-019 was observed to stabilize a rotamer of Y299, which is conserved in several other class A GPCRS as a “bias switch,”^111^^,^^112^ in a manner that is distinct from the rotamer observed in the peptide complex structures, and possibly explains the G-protein biased agonist activity of CMF-019.

Cryo-EM has become a key method for the determination of GPCR structures, opening up new avenues of investigation in structural biology, including the study of transient intermediates, multiple conformations from a single preparation, and determination of flexible complexes.^113^

Cryo-EM single-particle analysis, at a global nominal resolution of 3.6 Å, of apelin receptor protein preparations in the presence of the synthetic small molecule ligand, cmpd644, or endogenous ELA-32 peptide, demonstrated that the receptor exists more abundantly as a labile dimeric species than a monomer (in a 4:1 dimer:monomer ratio).^40^ Within this dimer, only one protomer engages with the G protein and accommodation of 2 G protein complexes is likely impossible because of steric constraints introduced by the dimer interface, but the overall organization of the dimeric G_i_ complex is otherwise similar to the monomeric G_i_ complex. The dimer interface itself was shown to be very small, consisting of protein–protein interactions mainly at the tip of transmembrane domain 3 and intracellular loop 1, governed by only 5 residues of each protomer, with the “dimer-switch” receptor residue F101 appearing to be particularly important in stabilizing the interface. In line with previous findings, single-particle analysis of the receptor in the absence of ligand indicated that dimerization is ligand-independent.

Binding of ELA to the apelin receptor has also been explored in docking and binding studies.^114^ Key findings by the authors showed that the F residue at position 10 of the ELA-11 peptide binds to a groove distinct from that of the C-terminal F of apelin-13, and the C-terminal P residue of ELA-11 may bind to the F13 apelin-13 pocket. G protein and *β*-arrestin assays revealed the necessity of a P or F aromatic residue at the C-terminal end of the peptides for receptor activation. Further exploration of the ELA-32-bound structure revealed that the 11 C-terminal amino acids of the peptide govern molecular interactions with the receptor, with the F31 residue of ELA-32 being observed to protrude deeper down into the large aromatic hydrophobic pocket than the terminal F17 residue of the apelin analog, AMG3054.^40^ Key receptor interaction residues, W85, Y88, Y271, F110, Y264, R168, and K268 are engaged by both ELA-32 and AMG3054, but, intriguingly, an artificial mutation of the dimer-switch residue, F110A, was shown to reduce the potency of ELA-32 by ∼10-fold whilst having no impact on apelin-13. The biological implications of this remain to be studied.

In another study, cryo-EM was used to resolve the structures of apelin receptor-G_i_ complexes in the presence of apelin, the G protein biased peptide MM07, and the G protein biased small molecule CMF-019, at resolutions of 3.2, 2.6, and 2.7 Å, respectively.^42^ Although the 3 agonist-bound structures had similar overall structures, key differences in the recognition modes of the agonists were identified. In brief, the findings highlighted the enhanced interactions between the G protein-biased agonists and receptor residues of TMD6/7 in the binding pocket, allosterically impacting the conformation of the intracellular half of TMD7, and ultimately forming a conformation that favors accommodation of the G protein. This was shown to be tightly regulated by “twin hotspots” in the receptor, with involvement of several residues including, crucially, Y299. Guided by the structures and role of the twin hotspots, the authors rationally designed the G protein biased ligands, WN353 and WN561 (Table 1), which were shown to adopt the desired poses using cryo-EM of the receptor in complex with these ligands. WN651 was taken forward into in vitro and in vivo experiments, demonstrating reduced *β*-arrestin-dependent hypertrophy of cardiomyocytes versus established apelin receptor agonists. Sun et al^43^ aimed to design completely G protein biased apelin agonists that may be beneficial in heart failure with preserved ejection fraction (HFpEF) (see sections Biased agonists: Small molecules and heart failure). Using cryo-EM they also resolved a high-resolution CMF-019-apelin receptor-G_i_ complex at 2.9 Å combined with mutagenesis of key amino acids involved in binding of CMF-019 to identify residues that contribute to either G protein (eg, S298 and Y299) or *β*-arrestin (eg, Y264-K268 electrostatic interaction) signaling. They went on to use molecular dynamics, cheminformatics and a Split-and-Mix based strategy to develop G protein biased small molecule agonists (including AP-7 and AP-16, Table 1) lacking the ability to recruit *β*-arrestin and with improved pharmacokinetics and bioavailability that showed further benefit over CMF-019 in in vitro and in vivo models of hypertrophy and fibrosis.

Overall, the comprehensive findings from the studies outlined above showcase the impetus of techniques such as X-ray crystallography and cryo-EM in structural biology to guide our understanding of protein-ligand interactions and rational drug design.

### B Apelin receptor variants

GPCRs are susceptible to natural mutation, and more than 2350 mutations across 55 different GPCRs have been associated with over 60 different human diseases.^115^ Although a number of artificial mutational techniques such as site-directed mutagenesis and alanine scanning mutagenesis have provided highly valuable insights into the key residues regulating protein binding and function, few naturally occurring apelin receptor variants have been identified or characterized.

Aligning with the finding that G protein-coupled Apelin receptor (Agtrl1b), the zebrafish (*D rerio*) homolog of the human apelin receptor, is critical for proper formation of the heart during gastrulation,^116^ researchers identified a naturally occurring, single allele of a novel recessive mutation, *grinch*^*s608*^*,* in zebrafish.^117^ Sequencing of *agtrl1b* cDNA showed that the *s608* allele contains a G to T base pair change at position 269, inducing a W to L amino acid change in the second transmembrane domain. In contrast to the wild-type gene, the *grinch*^*s608*^ allele of *agtrl1b* was shown to be completely unresponsive to stimulation by apelin peptide in in vitro assays. Phenotypically, *grinch*^*s608*^ mutant zebrafish were found to possess a reduced number of myocardial progenitor cells, resulting in a profound deficit in cardiomyocyte number and closely phenocopying zebrafish in which *agtrl1b* expression is synthetically downregulated.

Later, W85, the human apelin receptor residue corresponding to the conserved W residue mutated in the Agtrl1b *grinch*^*s608*^ zebrafish mutation, was shown to form one of the critical contact points between the receptor and the apelin peptide mimetic, AMG3054.^40^ As described in section X-ray crystallography and cryo-electron microscopy, W85 forms part of the hydrophobic cavity that engages with the C-terminal amino acid of the peptide. Alanine mutagenesis of this residue (W85A) resulted in abolished signal in response to apelin-13 and ELA-32 in cAMP assays, consistent with the *grinch*^*s608*^ mutation.

In addition to crystallizing the apelin receptor in complex with the G protein-biased small molecule agonist, CMF-019 (see section X-ray crystallography and cryo-electron microscopy), Williams et al,^28^ identified and characterized naturally occurring human apelin receptor variants to further determine structural and functional differences between peptide and small molecule ligand binding mechanisms. In the study, whole genomes of patients with rare diseases, sequenced by the NIHR BioResource project (a prospective component of the UK Genomics England 100,000 Genomes Project), were screened for sequence variants in the *APLNR* gene that were ultrarare (occurring at a minor allele frequency of <1 in 10,000 in the general population) and predicted to be deleterious.

**Fig. 5:**
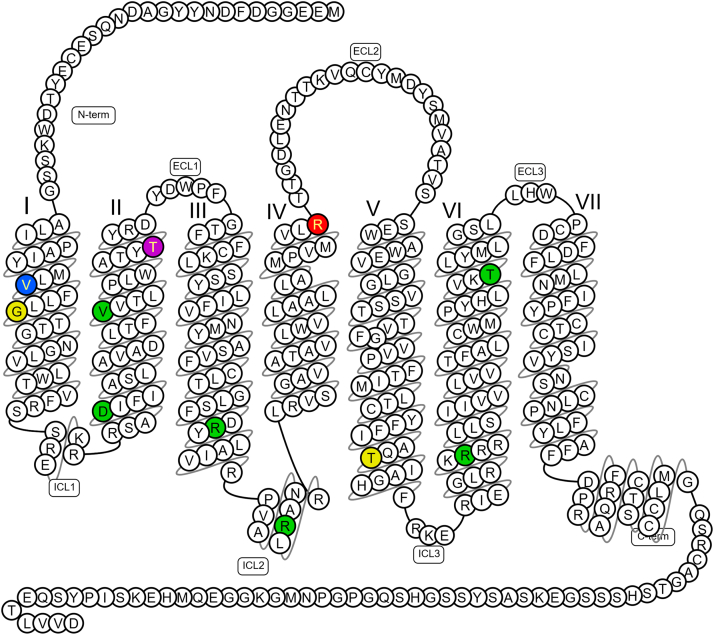
Snake plot showing the location of the naturally occurring variants in the human apelin receptor identified in whole genomes of patients with rare diseases sequenced for the UK Genomics England 100,000 Genomes Project (see section X-Ray Crystallography and Cryo-Electron Microscopy; Williams et al^28^). Key to amino acids: Red: missense mutation R/H168^4.64^ able to bind biased small molecule agonist CMF-019 but not endogenous peptides, apelin or ELA. Purple: missense mutation T/M89^2.64^ able to bind apelin but not ELA. Blue: V/L38^1.42^ able to bind both peptide ligands. Yellow: single nucleotide deletion frameshift mutations (G/X45^1.49^ and T/X227^5.64^) with expected reduced apelin binding. Green: missense mutations expressed on cells surface but did not alter apelin binding D65V^2.40^, V79M^2.54^, R127H^3.50^, R139W^34.55^, R243W^6.30^, T269M^6.56^. Image generated from data in GPCRdb,^109^ with superscript showing the Ballesteros–Weinstein numbering system for 7 transmembrane helices (1-7) and amino acids.^110^ ECL, extracellular loop; ICL, intracellular loop.

This approach yielded ∼50 variants, with 11 tested in preliminary binding assays (Fig. 5). From this, 6 human apelin receptor variants—2 comprising single nucleotide deletion frameshift mutations (G/X45^1.49^ and T/X227^5.64^) and 3 comprising missense mutations (V/L38^1.42^, T/M89^2.64^, and R/H168^4.64^) were characterized using multiplatform in vitro and in silico techniques. High content screening, used in conjunction with fluorescent apelin647 and ELA647 ligands (described in further detail in sections Fluorescently labeled apelin peptide analogs and fluorescently labeled Elabela/Toddler peptide analogs, respectively) showed that, not unexpectedly, the frameshifted variants G/X45^1.49^ and T/X227^5.64^ were expressed at lower levels, and bound ligands to a lesser extent than wild-type receptor. V/L38^1.42^ was able to bind both fluorescent ligands. Meanwhile, R/H168^4.64^ abolished binding of both apelin647 and ELA647, and, intriguingly, T/M89^2.64^ was able to bind apelin647 but not ELA647, with these findings confirmed using radioligand binding assays.

On top of this, AlphaFold2 models provided a structural rationale for the altered binding at T/M89^2.64^ and R/H168^4.64^. Modeling of the T/M89^2.64^ variant suggested that the methionine substitution narrows the binding pocket for ELA peptide, creating a steric clash with F13 of the peptide, but not for apelin, which features a less bulky proline residue at this position. In models of complexes with apelin and ELA, the R168^4.64^ guanidinium group falls within hydrogen bonding distance of the peptide C-terminus, strongly interacting with the last carbonyl and the terminal carboxyl and emphasizing R168^4.64^ as a key determinant of apelin receptor binding to both endogenous peptides. The modeling complemented previous experimental structures to predict the detrimental impact of the variants on endogenous peptide binding, closely aligning with the pharmacological data.

Next, the R/H168^4.64^ variant was assessed in a surface plasmon resonance assay, where it was again shown to tightly regulate binding of peptide ligands, but with less impact on the binding of small molecules. This aligned with findings from the crystal structure showing that R168 makes only a minor indirect interaction with CMF-019. Finally, the authors used CRISPR base editing to introduce the R/H168^4.64^ variant into human embryonic stem cell-derived cardiomyocytes (hESC-CMs), which endogenously express the apelin receptor (see section The apelin receptor in embryonic stem cells). The R/H168^4.64^ variant line exhibited robust expression of the receptor protein but showed reduced binding of fluorescent apelin647 and radiolabeled apelin versus wild-type hESC-CMs. Further, the introduction of the variant induced a detrimental phenotype, reducing differentiation efficiency from stem cells to beating cardiomyocytes, increasing compensatory secretion of apelin peptide, and prolonging time-to-peak voltage, indicating that the R/H168^4.64^ variant line performs more poorly than wild-type hESC-CMs. The authors speculate that, given the ability of the R/H168^4.64^ variant to still bind small molecules such as CMF-019, ligands could be designed that bind and activate the protein to rescue detrimental phenotypes caused by mutations that perturb endogenous signaling.

Although considerably more work is required to better understand the impact of naturally occurring variants of the apelin receptor, it is clear that exploiting genetic data can provide unique insights into key structural determinants of the binding and function of the protein and could potentially guide targeted design of new drugs and medicines.

## III Endogenous agonists

### A Apelin

#### 1 Synthesis and metabolism

About 5 years after the receptor was discovered, apelin-36 was identified from bovine stomach extract as the first endogenous ligand of the human apelin receptor.^2^ It was subsequently demonstrated that the apelin peptides were initially produced as a 77-amino acid preproprotein containing a signal peptide, which was removed by endopeptidases to make the proapelin, apelin-55 and eventually, the mature apelin, apelin-36 (Fig. 1). Although the mechanism of apelin metabolism is not fully known, several enzymes have been suggested to cleave apelin peptides including proprotein convertase subtilisin/kexin-3,^118^ plasma kallikrein,^119^ and angiotensin-converting enzyme 2 (ACE2).^60^^,^^120^, ^121^, ^122^ Consequently, several smaller but functional apelin isoforms have been produced, including apelin-12, apelin-13, [Pyr^1^]apelin-13 and apelin-17.

The apelin isoforms are widely distributed in mammalian systems. For example, [Pyr^1^]apelin-13 was identified as the most predominant isoform in rat plasma and hypothalamus^123^; human heart,^5^ and plasma.^124^ Lower levels of apelin-17 were also identified in rat hypothalamus and plasma.^123^ Interestingly, apelin-55 (proapelin), which was initially predicted as being inactive, was identified using liquid chromatography-mass spectrometry in mature bovine milk as the predominant apelin isoform along with apelin-22.^125^ The authors also identified many other shorter isoforms of apelin-55 (albeit at lower levels compared with apelin-55), suggesting that the proapelin was subsequently cleaved into shorter (and more active) fragments by endopeptidases in milk. Recently, apelin-55 was shown to retain biological activity at the apelin receptor,^126^ further extending the range of functional isoforms.

After infusion of 135 nmol/min [Pyr^1^]apelin-13 into human volunteers, for 120 minutes, a highly sensitive method using liquid chromatography–tandem mass spectrometry determined that the concentration in the plasma at the end of the infusion was 58 ng/mL despite the short half-life of the peptide. Previous reports in rodents had indicated that N-terminal metabolites were most abundant. In contrast in this study in humans the ACE2 C-terminal cleavage product, [Pyr^1^]apelin-13_(1-12)_, was the predominant metabolite detected in plasma (as well as [Pyr^1^]apelin-13_(1-10)_ and [Pyr^1^]apelin-13_(1-6)_), together with neprilysin N-terminal metabolites [Pyr^1^]apelin-13_(4-13)_ and[Pyr^1^]apelin-13_(6-13)_ at lower levels.^127^

#### 2 Radiolabeled apelin peptides and analogs

Apelin-13 and [Pyr^1^]apelin-13 have been radiolabeled with ^125^I and have retained high affinity binding and selectivity for the apelin receptor, as well as modified peptide analogs (Table 1). These compounds have been used for mapping the distribution of apelin receptors and have the advantage over antisera that the density (B_max_) can be measured either in homogenates of tissue or within sections by autoradiography and image analysis. These ligands bound with a single high affinity with no evidence of further subtypes.

Commercially available [^125^I][Glp^65^Nle^75^,Tyr^77^]apelin-13 (Revvity Inc) is labeled via a Tyr substituted for the C-terminus Phe^13^ to permit direct labeling by ^125^I. Norleucine (Nle) is substituted for Met^11^ (Fig. 1) to prevent oxidation during the labeling by the lactoperoxidase method. The radioligand bound with high affinity and is widely used in screening in competition binding assays (see for example, Williams et al^28^).

In human heart (left ventricle), binding using this radioligand was time-dependent and reversible. The median association rate constant (*K*_obs_) was 0.038 min^−1^ and half-time for association (*t*_1/2_) was 18.2 minutes. Binding of the ligand was reversible, with a median dissociation rate constant (*K*_−1_) 0.021 min^−1^, giving a half-time for dissociation (*t*_1/2_) of 33.67 (30.77–68.93) minutes.^128^ Very similar results were obtained by in human heart by Katugampola et al^15^ where [Pyr^1^]apelin-13 was labeled at an alternative site to the C-terminus (Lys^8^, Table 1) suggesting labeling position did not affect binding parameters.

The densities of apelin receptors (B_max_) measured by ligand binding assays in human tissues, are comparatively low compared with other vasoactive peptides such as endothelin. Examples of B_max_ values in human tissues (in fmol/mg): kidney 15.4,^129^ coronary artery 6.1, saphenous vein 5.7^5^; lung 4.24, heart 3.9,^13^ and in the brain, 3.8.^12^

#### 3 Fluorescently labeled apelin peptide analogs

Fluorescently labeled ligands are valuable experimental tools for studying GPCR pharmacology that can be used in a wide range of settings, from flow cytometry to simple in vitro binding and kinetic assays to high-throughput plate-based platforms and high content screening.^130^ Considerable improvements in the brightness and stability of fluorescent dyes, conjugation techniques, and optical equipment and quantification, have meant fluorescent ligands are emerging as safer, easier, and more versatile tools versus other classical techniques such as radioligand binding.^131^^,^^132^ Further, the localization of fluorescent ligands at both the cell and subcellular level can be visualized in real-time, with colocalization of multiple other markers in a single biological system.^133^

Fluorescently labeled apelin peptide analogs have been used with much success. Fluorescent lissamine-apelin 13 was shown to retain its binding and agonist activity after being used to induce and track endocytosis of rat apelin receptor stably expressed in CHO cells and tagged with enhanced green fluorescent protein, overlapping perfectly with the receptor fluorophore in vesicular structures.^106^ Notably, the authors also showed that lissamine-apelin 13 ligand did not induce endocytosis in a W259A apelin receptor variant, confirming the importance of this receptor residue for receptor internalization in findings that were supported by radioligand binding. Members of the same group later developed convenient labeling methods to synthesize and optimize fluorescent apelin ligands with subnanomolar affinities, enabling efficient tracking of the receptor in live cell imaging and time-resolved fluorescence resonance energy transfer imaging-based competition for high-throughput screening and drug discovery.^134^^,^^135^

A further study described the design and comprehensive validation of 4 novel fluorescent apelin receptor ligands, 2 based on a modified [Pyr^1^]apelin-13 analog (apelin488 and apelin647), and 2 based on ELA-14 (ELA488 and ELA647).^14^ See section Fluorescently labeled Elabela/Toddler peptide analogs below for further details on fluorescent ELA ligands. The ligands were tagged at the N-terminal end with bright and highly stable Alexa Fluor fluorophore dyes via a 4-mer polyethylene glycol (PEG4) linker. All 4 fluorescent ligands retained the ability to bind and activate the apelin receptor and, crucially, triggered receptor internalization. Apelin647 visualization was validated in a high-content screening system, using CHO-K1 cells stably expressing the apelin receptor to confirm specific, concentration-dependent binding. The endogenous peptides ([Pyr^1^]apelin-13 and ELA-14), the reported peptide antagonists (MM54 and F13A), and the G protein-biased small molecule agonist (CMF-019), were all shown to compete for binding in a concentration-dependent manner against apelin647 in this set-up, indicating potential tractability as a high-throughput platform for identifying novel apelin receptor ligands in the drug discovery pipeline.

On top of this, a 300 nM concentration of the ligand was subsequently used to visualize receptor internalization in near real time, moving rapidly from the membrane compartment to the cytosol over a 90-minute time course.^14^ Apelin647 was also used to specifically bind and visualize endogenously expressed apelin receptor in hESC-CMs, whilst apelin488 was used similarly in human kidney sections, localizing to receptors expressed in blood vessels and tubules of the renal cortex. These findings provide proof-of-principle for the use of fluorescent ligands as a potential alternative to antibodies that can be readily employed in live cells or frozen tissue.

### B Elabela/Toddler

#### 1 Synthesis and metabolism

*Apela* gene, which is located on chromosome 4, has 3 exons. It is translated into a 54 amino acid peptide, which is further processed by enzymatic removal of the signal peptide to ELA-32, the mature isoform (Fig. 2). ELA-32 has 2 potential furin cleavage sites marked by dibasic residues and was therefore predicted to undergo further processing by furin into ELA-21, ELA-11,^8^^,^^9^ and recently ELA-22.^52^ However, only ELA-11 has been observed endogenously in embryos expressing *apela* mRNA by mass spectrometry.^9^ The C-terminal sequence of ELA peptides, especially ELA-11, is well conserved in all vertebrates, suggesting that this region was critical to biological function.

Since ELA is a recent discovery, the distribution of *Apela* mRNA and the peptide in human and rodent tissues has not yet been thoroughly investigated. However, ELA mRNA is developmentally regulated, with the inner cell mass of the blastocyst showing the highest expression and is downregulated upon differentiation.^136^ Initial studies found that, in addition to its expression during embryonic development, ELA is also present in adult human kidney and prostate tissues.^8^^,^^101^ *APELA* transcripts have been reported in human blood vessels, with the highest levels detectable in arteries compared with veins, and lower levels in the human heart and lung tissue.^60^ At the protein level, ELA is localized to the endothelium of human heart and lungs vessels.^13^

In the rat, Deng et al^24^ suggested that ELA was exclusively expressed in the adult kidney compared with very low levels of apelin and apelin receptor. However, a subsequent study demonstrated that although ELA mRNA was detectable in the adult rat heart (albeit at lower levels compared with apelin), this expression was mainly localized to noncardiomyocytes, especially endothelial cells and fibroblasts.^24^ Thus, ELA may also have extrarenal expression, although the peptide is very likely to be synthesized by renal cells (hence the much higher levels in the kidney).

#### 2 Radiolabeled ELA peptide analogs

ELA-14 (YQRRC-Nle-PLHSRVPFP) modified by the addition of a Tyr at the N-terminus and substitution of Nle for Met^11^ was radiolabeled using the same lactoperoxidase method and purified by reverse phase high-pressure liquid chromatography. ELA-21 also modified with an N-terminus Tyr (YLRKHNCLQRRCMPLHSRVPFP) was also labeled. However, no specific binding was detected with either radioligand using a screen of human surgical samples that expressed high densities of the apelin receptor, such as human kidney, using a range of buffers and binding conditions (Davenport et al, unpublished). To date there have been no reports of ELA peptides being successful radiolabeled. In the absence of radiolabeled ELA, the peptide isoform were tested in competition with [^125^I][Glp^65^Nle^75^,Tyr^77^] apelin-13. In human left ventricle, all isoforms bound with a single high affinity. ELA-32 (pK_i_ = 9.59) had a significantly higher affinity than ELA-21 (pK_i_ = 8.52) but comparable to [Pyr^1^]apelin-13 (pK_i_ = 8.85). Interestingly, ELA-11 (pK_i_ = 7.85) had a significantly lower affinity than the other peptides.^13^

#### 3 Fluorescently labeled ELA peptide analogs

The application of fluorescent labeling has been explored to a lesser extent for the second endogenous apelin receptor ligand, ELA, versus apelin. Nevertheless, 2 fluorescent ligands, ELA488 and ELA647, were characterized alongside apelin488 and apelin647 fluorescent ligands described in section Fluorescently labeled apelin peptide analogs.^14^

ELA488 and ELA647 were designed using the endogenous ELA-14 amino acid sequence and again were tagged at the N-terminal end with Alexa Fluor dyes via a PEG4 linker.^14^ Similarly to apelin488 and apelin647, the ELA488 and ELA647 fluorescent ligands were shown to retain binding and function at the apelin receptor in radioligand competition binding assays and in vitro activation assays. Crucially, ELA488 was shown to bind specifically to apelin receptor expressed in the blood vessels and tubules of the renal cortex of human kidney sections, in a similar manner to apelin488.

As described in section Apelin receptor variants, apelin647 and ELA647 were further used to identify discrepancies in binding at the T/M89^2.64^ naturally occurring human apelin receptor variant, where ELA647 was shown to not bind to the variant, whilst apelin647 binding was retained.^28^ The findings highlight the potential for fluorescent ELA ligands as useful tools that could enable the dissection of key similarities and differences in apelin receptor binding and function in response to its 2 distinct endogenous ligands.

### C Plasma levels of apelin and ELA as biomarkers

Changes in plasma apelin immunoreactivity measured by ELISAs in clinical conditions compared with healthy controls are used as a potential biomarker to identify an association between a particular disease and the apelin signaling pathway. Commercial assays typically employ polyclonal antibodies raised against various synthetic peptide fragments. Circulating immunoreactive apelin may also reflect crossreaction to shorter metabolites (section synthesis and metabolism) but information as to the contribution of these to the signal is not always available. Levels measured in plasma may also be affected by whether or not the various extraction methods were used. Raised plasma levels may not necessarily translate to an increased binding to the apelin receptor.

#### 1 Heart failure

In humans, the first connection between apelin and heart failure (HF) came from Foldes et al,^137^ who demonstrated that both apelin and apelin receptor levels were upregulated in heart tissues from patients with chronic HF caused by coronary artery disease or idiopathic dilated cardiomyopathy. In patients with HF, apelin levels were increased in the initial stages of the disease development and decreased in more advanced disease.^55^^,^^138^ This supports the compensatory role of apelin in restoring cardiac contractility in the early stages of HF, which is lost in more advanced disease. Indeed, in patients with chronic HF, plasma apelin was positively correlated with response to cardiac resynchronization therapy as evidenced by improved left ventricular remodeling and ejection fraction.^139^ It was subsequently showed that apelin decreased blood pressure and peripheral vascular resistance while increasing cardiac output in patients with HF.^57^ Additionally, a randomized controlled study showed that apelin had beneficial cardioprotective effects in patients with chronic HF by decreasing mean arterial pressure, peripheral resistance, and improving cardiac function (fractional shortening and ejection fraction).^59^

A potential biomarker role has also been described for apelin in other cardiovascular diseases. For example, 1 study has shown that circulating levels of apelin are reduced in patients with atrial fibrillation compared with those without atrial fibrillation or healthy subjects.^140^ The authors further demonstrated that circulating apelin and N terminal pro B-type natriuretic peptide were independent predictors of atrial fibrillation, therefore suggesting that plasma apelin could be a potential diagnostic biomarker for atrial fibrillation. Apelin-17 was also suggested to be a potential diagnostic biomarker for idiopathic pulmonary arterial hypertension (PAH),^141^ although circulating levels of apelin-12^142^ and apelin-36^143^ were also reduced in patients with pulmonary hypertension (PH) compared with healthy subjects.

Similar to apelin, circulating levels of ELA has been shown to be significantly reduced in patients with atrial fibrillation and HF compared with controls with this decrease negatively correlating with B-type natriuretic peptide levels.^144^, ^145^, ^146^ Hence, because increased B-type natriuretic peptide levels are predictive of high HF risks, ELA signaling such as, apelin has a protective role in this condition.

#### 2 Kidney disease

The circulating levels of apelin peptides in patients with CKD remain controversial. One study reported a decreased plasma apelin, which was associated with cardiac function in patients with CKD on dialysis.^147^ However, another study did not find any change in levels of apelin-36 in these patients although the levels of apelin-12 were significantly higher compared with healthy controls, and these did not associate with cardiovascular risk.^148^ In kidney allograft patients with coronary artery disease, apelin was found to be significantly decreased and independently associated with markers of endothelial dysfunction or inflammation.^149^ These studies may suggest that changes in circulating apelin in CKD were dependent on the level of endothelial damage since the endothelium is the primary source of apelin.^150^ In support, a recent study investigated the levels of apelin, asymmetric dimethylarginine and oxidized low-density lipoprotein receptor 1 and their associations with markers of renal damage and cardiovascular dysfunction, and found that both plasma apelin and asymmetric dimethylarginine were increased in hemodialysis patients compared with healthy controls.^151^ Interestingly, plasma apelin level was negatively correlated with glomerular filtration rate (used as surrogate marker of renal function). Therefore, exogenously increasing apelin levels in patients with kidney disease may offer renoprotection.

In patients with autosomal dominant polycystic kidney disease, circulating apelin was significantly lower and inversely correlated with vasopressin levels compared with healthy subjects.^152^, ^153^, ^154^ Follow-up evaluation of the patients showed that decreased circulating apelin independently correlated with tubular damage and disease severity.^153^

#### 3 Cancer

As previously indicated in the introduction, the role of apelin signaling is complex. In various forms of cancer, the expression of apelin and apelin receptor are increased, and this has been associated with increased angiogenesis, proliferation, metastasis, induction of cancer stem cells and drug resistance (for a detailed and extensive review see Naldi et al^85^ for information on changes in tissue peptide levels of apelin and its receptor. For changes in plasma in cancer, see the article by Masoumi et al^155^).

Grinstead and Yoon^156^ reviewed apelin as a systematic biomarker in cancer but note that this is limited by lack of accepted method of standardization in methods of measuring apelin levels and how changes are compared in healthy versus patients with cancer. However, there is emerging evidence that comparing levels of apelin gene expression in tumor samples matched to adjacent normal tissues is more informative that in sera or plasma. For example, Feng et al,^157^ have linked apelin mRNA levels to the clinical features and prognosis of gastric cancer. In addition, recent studies have shown using new techniques of single cell and spatial transcriptomics that apelin expression was not only increased in human hepatocellular carcinoma but also that liver cancers characterized by *APLN* expression rapidly progressed beyond stage 2.^158^^,^^159^ Interestingly, in human hepatocellular carcinoma, the apelin activated the phosphoinositide 3-kinase (PI3K)/protein kinase B (Akt) signaling pathway, resulting in enhanced cell proliferation.^160^ The apelin antagonist ML221 inhibited this pathway, supporting this cancer as a potential target for therapeutic intervention. However, *APLN* expression may not be increased in all cancer types as Mao et al^158^ also found that its levels were reduced in renal papillary cell carcinoma, lung adenocarcinoma, and lung squamous cell carcinoma. This suggest that the role of apelin as a potential oncological biomarker may vary depending on the cancer type and single cell and spatial transcriptomics techniques may be more robust and informative than measuring peptide levels.

#### 4 Preeclampsia

Several studies were performed to delineate the role of ELA in preeclampsia, especially in humans by measuring the circulating levels of ELA in pregnant women but produced contrasting findings. Although some studies did not find any correlation or changes in ELA levels in pregnant women with preeclampsia,^161^^,^^162^ others found a decrease^163^^,^^164^ or an increase^165^ in circulating ELA levels in these women. Interestingly, it seems that circulating ELA levels in women with preeclampsia correlate with their body mass index (BMI). For instance, Zhou et al^164^ studied women with BMI <25 kg/m^2^, Panaitescu et al^165^ studied women with BMI ≥28 kg/m^2^, and Pritchard et al^161^ studied women with a BMI between 23 and 31 kg/m^2^, while other studies did not specify the BMI range of women involved in the study. Pregnant women with healthy BMI (<25 kg/m^2^) who would later experience preeclampsia had decreased ELA levels in the first trimester,^166^ but it is unclear whether their BMI had increased significantly at the time they experienced preeclampsia.

Circulating levels of apelin have also been studied in context of preeclampsia. However, a recent systematic review and meta-analysis study examining 122 of these published articles on circulating apelin in pregnant women with preeclampsia did not find any significant difference between the preeclamptic and normotensive women.^167^ However, their subgroup analysis found that circulating apelin levels were significantly lower in preeclamptic pregnant women with higher BMI but apelin did not significantly impact disease severity.^167^ Additionally, although apelin deletion in rodents does not affect prenatal development or cause preeclampsia-like symptoms, the loss of ELA affected prenatal development and caused preeclampsia-like symptoms,^168^ suggesting that in the context preeclampsia, ELA may be the more relevant of the 2 peptides. However, it remains to be determined which downstream signaling pathways mediate these differential effects of ELA and apelin.

## IV Development and genetic modifications

### A Genetically modified mice

**Fig. 6:**
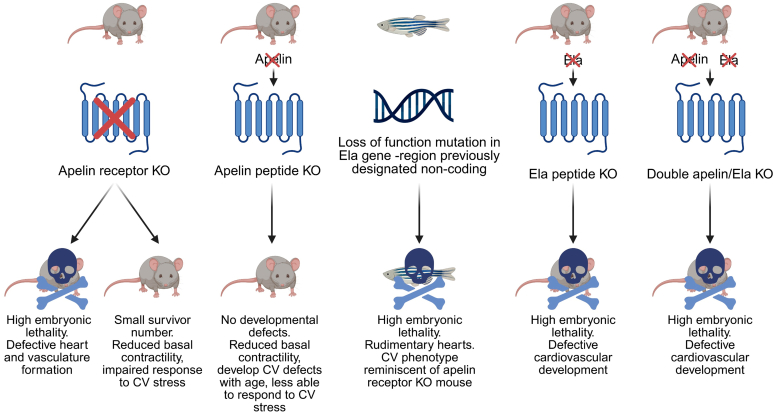
Summary of the distinct phenotypes arising from deletion of genes encoding the apelin receptor (*Aplnr*), apelin (*Apln*), and ELA (*Apela*) summarizing the loss of apelin receptor and its ligands on cardiovascular (CV) development. The result of deleting the gene encoding in the zebrafish is shown for comparison. For further information on the effects of genetic manipulation of apelin peptides and receptor in mice see Zhong et al^82^ (Figure created in BioRender. Davenport, A. (2025) https://BioRender.com/jjyj0a5).

Previous studies have investigated knockout (KO) of the apelin receptor, apelin, and ELA peptide genes, each resulting in distinct phenotypic outcomes (Fig. 6).

A number of studies have previously shown that homozygous knockout of the apelin receptor in mice results in significant embryonic lethality.^169^, ^170^, ^171^ Embryonic lethality occurred mainly between E10.5 and E12.5,^171^ with the embryos displaying a range of cardiovascular defects, including abnormal heart formation and poor vascularization of the yolk sac. Those mice that did survive to neonates also displayed cardiovascular issues, including thinning of the myocardium, ventricular defects, reduced capillary density and significant decreases in vascular smooth muscle recruitment.^171^ There are, however, disparities between studies on the effect of apelin receptor KO in those mice that do survive to adulthood, with one study displaying right atrial enlargement and ventricular septal defects, whereas other studies showed the mice to be relatively normal,^170^^,^^172^ apart from reduced basal cardiac contractility and a significant impairment in ability to respond to cardiovascular stress (eg, exercise).^170^

Conversely, homozygous apelin peptide KO mice are born at expected Mendelian ratio and display normal cardiovascular development.^170^^,^^173^ In adulthood, the mice were found to be generally healthy, although similarly to surviving apelin receptor KO mice, responded poorly to cardiovascular stress and displayed reduced exercise capacity.^170^^,^^174^

The discovery of the second peptide ligand, ELA, resolved the differences in developmental phenotype observed between apelin receptor KO and apelin peptide KO mice. A loss of function mutation in the *apela* gene in zebrafish resulted in severe cardiac dysplasia, rudimentary heart formation, defective vascular formation, and an associated high embryonic lethality rate.^8^^,^^9^ It was noted that this zebrafish mutation strikingly resembled the phenotype of apelin receptor null mice and supported a critical role of the ELA peptide to activate the apelin receptor for cardiovascular development. Subsequently, ELA KO mice were generated and were found again to display prominent levels of cardiovascular defects and a high proportion of embryonic lethality.^168^^,^^175^ Interestingly, apelin does not appear to compensate for loss of ELA in development, with double KO of apelin and ELA found to result in similar phenotype to ELA KO mice.^175^ It is unclear what causes the cardiac abnormalities in ELA null mice, but it has been suggested that aberrant upregulation of erythroid and myeloid markers may be involved.^175^

### B The apelin signaling system in stem cells

There has been growing interest in the expression and role of the apelinergic system in different types of stem cells. A summary of select published literature to date is contained in Table 3.^176^, ^177^, ^178^, ^179^, ^180^, ^181^, ^182^, ^183^, ^184^, ^185^, ^186^, ^187^, ^188^, ^189^, ^190^, ^191^, ^192^, ^193^, ^194^, ^195^, ^196^, ^197^, ^198^, ^199^, ^200^, ^201^, ^202^, ^203^, ^204^, ^205^, ^206^ From these studies, insights into the role of the apelin signaling system in a number of biological processes and potential therapeutic applications can be gleaned.

**Table 3:** Summary of selected publications investigating the role of the apelin receptor in stem cells

Stem cell type	Process	Role of Apelin Signaling System	Reference
Human hemopoietic stem cells	Angiogenesis and formation of fine vascular network	Apelin interacts with apelin receptor to regulate caliber size of blood vessels	Takakura and Kidoya^176^
Human embryonic stem cells	Specification of ESCs toward cardiac lineage/mesoderm patterning	Apelin signaling act downstream of Cripto protein to promote mammalian cardiomyogenesis via mitogen-activated protein kinase/p70S6, governing mesoderm patterning	D’Aniello et al^177^
Mouse embryonic stem cells and human embryonic stem cells	Differentiation of ESCs to cardiac lineage	Apelin treatment in mESCs and hESCs enhances cardiac differentiation to form contractile embryoid bodies displaying expression of cardiac specific markers	Wang et al^178^
Rat BMSCs	Protection of BMSCs from apoptotic death	Apelin-13 has antiapoptotic effects in BMSCs via the MAPK/ERK1/2 and PI3K/Akt signaling pathways, which may have therapeutic significance in promoting survival of BMSCs in transplantation therapy	Zeng et al^179^
Human embryonic stem cells	Differentiation of ESCs to hematopoietic and endothelial cells	Mesodermal precursors derived from hESCs were enriched with *APLNR* gene expression, and apelin peptide promotes differentiation of hESC-derived hematopoietic and endothelial cells	Yu et al^180^
Mouse BMSCs	BMSC proliferation	Hypoxia induces BMSC proliferation via activation of the apelin receptor signaling pathway	Li et al
Human pluripotent stem cells	Differentiation of pluripotent stem cells to hematopoietic stem cells	Upregulation of apelin gene by Lhx2 transcription factor is correlated with hematopoietic commitment, and overexpression of apelin gene increases hematopoietic differentiation of pluripotent stem cells	Chen et al^182^
Human embryonic stem cells	hESC self-renewal	ELA is secreted by hESCs and acts in a paracrine fashion to promote growth, pluripotency and self-renewal	Ho et al^136^
Human mesenchymal stem cells	Differentiation of mesenchymal stem cells into endothelial cells	Apelin in combination with VEGF promotes differentiation of mesenchymal stem cells to endothelial cells	Park et al^183^
Rat endogenous cardiac stem cells	Mobilization of endogenous cardiac stem cells to mediate repair of myocardium	Apelin-13 via apelin receptor enhances mobilization and survival of endogenous cardiac stem cells to promote improved cardiac function post myocardial infarction	Zhang et al^184^
Mouse mesenchymal stem cells	Survival of transplanted mesenchymal stem cells	Apelin increased survival of mesenchymal stem cells in ischemic hindlimbs and promoted improved blood perfusion, thought to be via promoting protective autophagy, activating AMPK and inhibiting mammalian target of rapamycin	Liang et al^185^
Human mesenchymal stem cells	Differentiation of mesenchymal stem cells to cardiomyogenic lineage	In mesenchymal stem cells, apelin upregulates cardiac specific genes to via extracellular signal-regulated kinase 1/2 and 5	Wang et al^186^
Human mesenchymal stem cells	Survival of human mesenchymal stem cells	Apelin in combination with upregulation of VEGF promotes survival of human mesenchymal stem cells under hypoxic-ischemic conditions	Hou et al^187^
Human induced pluripotent stem cells (hiPSCs)	Differentiation of hiPSCs to endothelial cells	Single cell RNA-seq reveals that hiPSCs differentiated to endothelial cells form distinct subpopulations, with one population marked by robust enrichment of apelin receptor gene	Paik et al^188^
Rat mesenchymal stem cells	Expression of apelin in mesenchymal stem cells	Overexpression of apelin in mesenchymal stem cells improved insulin sensitivity and promoted endogenous pancreatic *β* cell proliferation in a rat model of type-2 diabetes	Gao et al^189^
Human BMSCs	Differentiation of human BMSCs to osteoblasts	Apelin enhances osteogenic differentiation of human BMSCs via the Wnt/*β*-catenin signaling pathway, which could be used to promote fracture healing	Hang et al^190^
Human hemopoietic stem cells	Hematopoietic stem cell maintenance	Removal of apelin positive endothelial cells results in decreased survival and differentiation of hematopoietic stem cells	Chen et al^191^
Human embryonic stem cell-derived cardiomyocytes	Contractility of hESC-derived cardiomyocytes	Addition of [Pyr^1^]-apelin-13 to 3D engineered cardiac tissues made from hESC-derived cardiomyocytes had no effect on contractility as measured by BioWire platform	Qu et al^192^
Rat mesenchymal stem cells	Survival of mesenchymal stem cells on exposure to hypoxic insult	Treatment of MSCs under hypoxic insult with ELA improves overall viability and induced antiapoptotic effects through action on the apelin receptor and activation of AKT and ERK	Fu et al^193^
Rat BMSC	Differentiation of BMSCs to osteoblasts	Apelin-13 revives osteogenic function via AMPK in a rat model of osteoporosis, promoting enhanced osteogenesis	Chen et al^194^
Mouse mesenchymal stem cells	Senescence of mesenchymal stem cells	Overexpression of apelin activates AMPK in aged MSCs in a state of senescence, promoting cardioprotection after myocardial infarction in mice	Zhang et al^195^
Mouse embryonic stem cells	Differentiation of mESCs to hematopoietic stem and progenitor cells	Hematopoietic stem and progenitor cells arise exclusively from apelin receptor expressing mesodermal cells, with subsequent downregulation of apelin receptor required to prevent myeloid differentiation	Jackson et al^196^
Human mesenchymal stem cells	Survival of mesenchymal stem cells under hypoxic insult	Treatment with apelin-13 improves survival of mesenchymal stem cells and reduced ROS generation under hypoxic conditions, promoting improved heart function and increased angiogenesis after myocardial infarction in a mouse model	Chen et al^197^
Rat endogenous neural stem cells	Proliferation and differentiation of neural stem cells	Apelin induces proliferation of neural stem cells in vitro, and promotes differentiation of neural stem cells in vivo post spinal cord injury	Liu et al^198^
Mouse embryonic stem cells	Differentiation of mESCs to mesoderm-derived cells	Apelin receptor signaling induces the necessary cell migration for germ layer formation and promotes differentiation to mesoderm via FGF/MAPK pathway	Sisli et al^199^
Human embryonic stem cell-derived cardiomyocytes	Development and contractility of hESC-derived cardiomyocytes	Knockdown of apelin receptor expression in hESCs reduced cardiomyocyte differentiation efficiency, associated with prolonged voltage sensing and asynchronous contraction. Apelin receptor knockdown in 3D engineered heart tissues was associated with reduced cardiac contractility	Macrae et al^200^
Mouse neural stem cells	Activation of neural stem cells for neural repair	Apelin overexpression increases proliferation of neural stem cells and promotes differentiation both in vitro and in vivo after traumatic brain injury, resulting in reduced lesion volume and apoptosis and neuroinflammation	Li et al^201^
Mouse embryonic stem cells	Differentiation of pluripotent stem cells to mesenchymal stem cells	Mesoderm commitment is promoted by apelin receptor signaling via EGFR and TGF-*β* signaling pathways, enhancing cellular migration. Apelin receptor signaling also upregulates MSC characteristic markers such as Wnt, PDGF and FGF. Apelin signaling activity promotes differentiation of mesoderm derived lineages including osteogenic, myogenic, and adipogenic cell lines	Sisli et al^202^
Rat mesenchymal stem cells	Mesenchymal stem cell proliferation and migration	Addition of ELA to MSCs increases proliferation, viability, and migratory activity, potentially via the METTL3/PI3K/AKT signaling pathway	Xu et al^203^
Human mesenchymal stem cells	Secretion of pro survival factors from mesenchymal stem cells	Secretion of apelin from mesenchymal stem cells can promote renal and hepatic function in rat model of diabetic nephropathy and hepatopathy	Karimi et al^204^
Human induced pluripotent stem cells	Neuroinflammation in hiPSC-derived neurons	In hiPSC-derived neurons from patients with Parkinson disease, stress-induced upregulation of apelin receptor expression was observed, along with increased apelin expression	Gerasimova et al^205^
Mouse mesenchymal stem cells	Survival of neonatal cardiomyocytes and cardiac function	Transplantation of exosomes sourced from mesenchymal stem cells pretreated with apelin increased heart function in a mouse model of sepsis-induced myocardial dysfunction, reduced cell death and exerted cardioprotective effects via miR-34a-5p	Li et al^206^

EGFR, estimated glomerular filtration rate; ERK, extracellular signal-regulated kinase; FGF, fibroblast growth factor; METTL3, methyltransferase-like 3; PDGF, platelet-derived growth factor; ROS, reactive oxygen species; VEGF, vascular endothelial growth factor.

#### 1 The apelin receptor in embryonic stem cells

In embryonic stem cells (ESCs), ELA has been shown to be expressed in both mouse ESCs (mESCs) and human ESCs (hESCs), where it has a role in promoting self-renewal.^136^^,^^181^ Additionally, knockdown of ELA in hESCs resulted in a loss of the ability to form teratomas, indicating a role for ELA in maintaining pluripotency.^136^ It was previously suggested that ESCs did not express apelin receptor, with 2 studies reporting no expression at the gene or protein level in 2 lines of hESCs, with upregulation only seen at mesoderm differentiation.^136^^,^^181^ Additionally, in HES3 and H9 hESCs, a lack of binding was seen for a fluorescently conjugated [Pyr^1^]apelin-13 analog.^180^ Conversely more recent studies have shown expression of apelin receptor and its ligands in ESCs. For example, Macrae et al^200^ found expression of the apelin receptor and its endogenous ligands at both the gene and protein level in hESCs by a combination of quantitative polymerase chain reaction, radioligand binding and ELISA.

#### 2 The apelin signaling system in development

The apelin signaling pathway has emerged as a key regulator of development, particularly of the cardiovascular system, with much of the knowledge of apelin signaling in development coming from the study of KO models as described in the previous section.

Apelin signaling plays a critical role in heart field formation, with experiments in zebrafish demonstrating that a critical gradient of apelin is needed for correct migration of mesodermal cells, fated to form the myocardium.^116^^,^^117^ Reduction in expression of apelin receptor gene led to disrupted cardiac structure organization and a decrease in myocardial progenitor cells, whereas exposure to excess apelin peptide was shown to disrupt gastrulation and completely inhibit myocardial cell differentiation. These early studies suggest that a tightly controlled apelin-apelin receptor interaction pathway is needed to mediate myocardial progenitor migration to the correct position for them to receive critical differentiation signals.^116^^,^^117^^,^^207^

The mechanism by which apelin signaling promotes mesoderm formation is still under investigation but it is thought to promote cardiomyogenesis acting downstream of Cripto protein via mitogen-activated protein kinase/p70S6,^177^ as well as inducing necessary cellular migration for germ layer formation through the fibroblast growth factor/mitogen-activated protein kinase (MAPK) pathway.^199^ Mesoderm commitment is also promoted via the apelin receptor through estimated glomerular filtration rate and transforming growth factor-*β* (TGF-*β*) pathways to enhance cellular migration and upregulate key mesenchymal stem cell (MSC) markers such as Wnt and platelet-derived growth factor.^202^

Once differentiated to MSCs, the apelin system has further roles in development. Apelin receptor signaling is thought to upregulate cardiac specific genes by activating extracellular signal-regulated kinase 1/2 and 5.^186^ Furthermore, activation of the apelin receptor by ELA peptide increases proliferation, viability and migratory activity of MSCs,^203^ which also has potential key roles in cardiac development. Additionally, mesodermal cells exposed to apelin peptide are found to differentiate to hematopoietic stem cells and endothelial cells,^180^ suggesting a further role of apelin signaling in vascular development. It has been shown that apelin can work in combination with vascular endothelial growth factor to promote differentiation of MSCs to endothelial cells.^183^

Studies using human hESCs have also demonstrated a role of apelin signaling in cardiomyocyte development. First, in both mESCs and hESCs, treatment with apelin was found to enhance cardiac differentiation to contractile embryoid bodies, complete with expression of cardiac specific markers.^178^ Furthermore, inducible knockdown of apelin receptor in hESCs was found to reduce the differentiation efficiency of cardiomyocytes and resulted in reduced cardiac contractility and prolonged voltage sensing.^200^

In addition, the apelin signaling system is thought to be involved in arterial-venous alignment and thermoregulation, with both apelin null and apelin receptor null mice less able to respond to hot or cold heat stress.^208^

#### 3 The apelin signaling system in neural protection

There have been several studies investigating the role of the apelin receptor in neural protection, through activation of the signaling system in neural stem cells. In a rat model, treatment of neural stem cells with apelin was found to induce cell proliferation and promote neuronal differentiation in vitro. The same study found that transfection of induced pluripotent stem cells as a means to prolong duration of apelin expression reduced neuroinflammation, reduced neuronal damage and promoted motor function, whilst also stimulating differentiation of endogenous neuronal stem cells in an in vivo rat model of spinal cord injury.^198^ Furthermore, another study demonstrated that transplantation of apelin-overexpressing neural stem cells on a nanofiber scaffold reduced the impact of traumatic brain injury. Mice were found to have reduced neuronal apoptosis, enhanced angiogenesis and decreased overall lesion volume,^201^ suggesting neural stem cell apelin therapy could promote an environment that can facilitate repair after neural damage.

#### 4 The apelin signaling system in osteogenesis

In recent years, there has been interest in the role of apelin signaling in osteogenesis via bone marrow-derived mesenchymal stem cells (BMSCs). Both overexpression of apelin and exposure to exogenous apelin resulted in increased osteoblast differentiation of human BMSCs in vitro. This increase in differentiation is thought to be due to activation of the Wnt/*β*-catenin signaling pathway, as inhibitors of this pathway attenuated differentiation.^190^ This effect is potentially useful for promoting fracture healing, as local exogenous apelin injection was found to promote bone healing in a rat model of a tibial fracture.^190^ An additional use case for apelin’s effect on osteogenesis was observed in a rat model of osteoporosis, where apelin-13 treatment was found to induce mitophagy in BMSCs via AMP-activated protein kinase (AMPK), relieving oxidative stress and promoting osteogenic function.^194^

## V Apelin receptor distribution

### A Distribution of apelin receptor mRNA and protein in human tissues

The human apelin receptor is encoded by the *APLNR* gene (Uniprot P35414, previously names include *APJ*, *APJR*, *AGTRL1*, and *HG11*), localized to chromosome 11q12 and comprises 380 amino acids. The gene was originally named angiotensin II receptor-like 1 as it was identified through sequence similarity (54%) in the transmembrane domains to angiotensin II receptor (AT_1_)^1^ but does not bind to the peptide. The receptor shares 89% sequence similarity with rat and 91% with mouse receptors.^7^ To date, no major differences in the pharmacology of endogenous peptides or synthetic receptor ligands have been reported between humans and rodents, supporting the use of these 2 species in translational research. For example, similar dissociation constant (K_D_), maximal density or receptors (B_max_) were measured in human and rat tissues.^15^

Apelin isoforms ranging from apelin-10 to apelin-36 were screened against orphan GPCRs in Family A including those with the highest sequence similarity after AT_1_ (GPR15 and GPR25) but no binding was detected.^209^ No receptor subtypes have been reported in other mammals. It is notable that there are 2 subtypes in nonmammalian species, for example, zebrafish,^210^ *aplnra* and *aplnrb*, with the latter showing the greater sequence similarity with the human receptor.^117^

This section of the review focuses on the localization of the genes encoding the apelin receptor and the 2 endogenous ligands in humans using bulk sequencing in tissues and single-cell RNA sequencing techniques to analyze gene expression at the level of individual cells. Studies are summarized where receptor protein has been localized by radioligand and fluorescent ligand binding as well as by immunocytochemistry for both peptides and receptor. A detailed comparison of the distribution of the expression of the receptor and mRNA in human tissues (as well as apelin and ELA) compared with rat and mouse using different techniques was compiled by Read et al.^53^

Medhurst et al^12^ used sensitive reverse transcription polymerase chain reaction (RT-PCR) to map expression of receptor mRNA in human tissues. In the CNS highest levels localized to the spinal cord, but with expression in all regions of the brain, such as the cortex, subcortical regions, midbrain, hindbrain, pituitary, and spinal cord.^12^ mRNA was widely distributed in the periphery including heart, lung, and kidney with highest levels in spleen and placenta. Northern analysis showed similar results with high levels of receptor mRNA detected in the spinal cord and corpus callosum of the brain as well as expression in the brain medulla, amygdala, hippocampus, substantia nigra, hypothalamus, and thalamus.^211^^,^^212^

Receptor mRNA was analyzed in rat CNS and peripheral tissues by RT-PCR.^10^^,^^12^ A detailed comparison of rat and mouse tissues showed generally a similar distribution by both in situ hybridization and radioligand binding in rats versus mice.^213^

The receptor was first characterized using [^125^I]-apelin binding in human tissue by Katugampola et al.^15^ In the cardiovascular system, receptors were visualized to the myocardium of the heart as well as the arteries and veins. The precise cellular localization of the receptor was confirmed using immunocytochemistry and dual confocal microscopy to human cardiomyocytes, vascular smooth muscle and to secretory vesicles of the constitutive pathway within the cytoplasm of endothelial cells.^214^ This is consistent with subsequent functional studies demonstrating the vasodilatory role of apelin in humans.

**Fig. 7:**
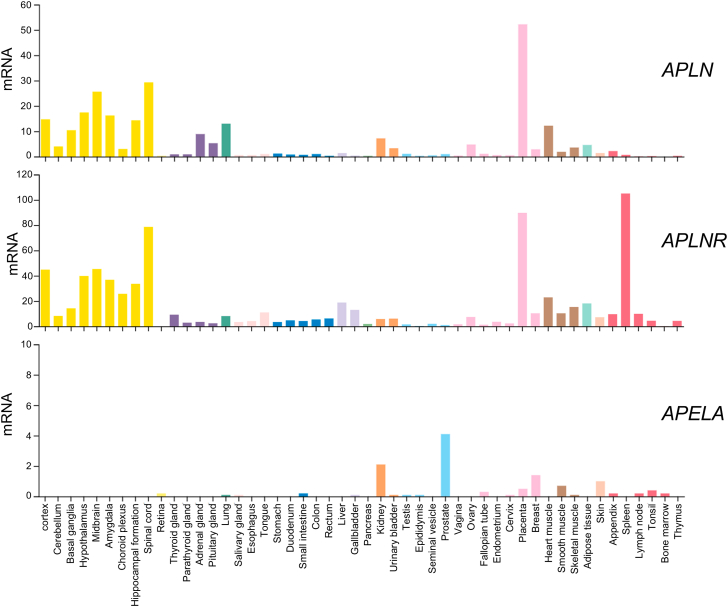
Expression profile of mRNA encoding the apelin (*APLN*), apelin receptor (*ALNR*), and ELA (*APELA*) in 60 normal human tissues and organs using data from the Human Protein Atlas.^215^ The consensus normalized mRNA expression is shown in transcripts per kilobase million (nTPM).

Expression data using bulk RNA-sequencing in the Human Protein Atlas^215^ that generates a large number of reads per sample that can detect low-abundance transcripts, such as those encoding GPCRs, is shown in Fig. 7. This allows a much larger number of regions to be compared, in this case 50 healthy human tissues. The data confirm this widespread distribution, together with the expected enrichment in the spleen and placenta in the periphery and the CNS, particularly the spinal cord. Most enriched is the placenta, with lower levels in heart, skeletal and smooth muscle, and adipose tissue. These data also illustrate the abundance of mRNA for both apelin peptide and receptor in human brain, where the role of apelin has been less extensively explored compared with peripheral tissues.

Combining results from these studies showed that the apelin receptor mRNA and protein was widely distributed in human tissues^53^ and consistent presence of the receptor on the endothelium and the role of apelin acting in an autocrine/paracrine manner to mediate vasodilation (see section *Clinical pharmacology of endogenous apelin agonists*).

### B Distribution of apelin receptor mRNA and protein in single cells

**Fig. 8:**
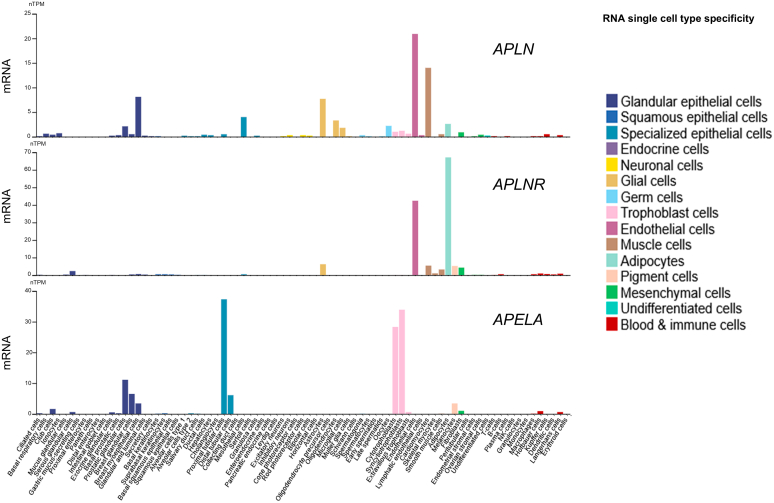
Expression profiles for apelin (*APLN*), apelin receptor (*ALNR*), and ELA (*APELA*) after single-cell mRNA sequencing (scRNA-seq) for 81 cell types from 31 human healthy tissues or organs from the Human Protein Atlas.^215^ Published data sets comprising >3000 cells with a depth of read 20 million counts for each tissue without pre-enrichment of cell types, using 10X Genomics were subjected to meta-analysis. Cell types were identified by a high correlation between the cluster-specific expression and the expected expression pattern of cell type–specific markers. Data are normalized expression in transcripts per kilobase million.^216^

**Fig. 9:**
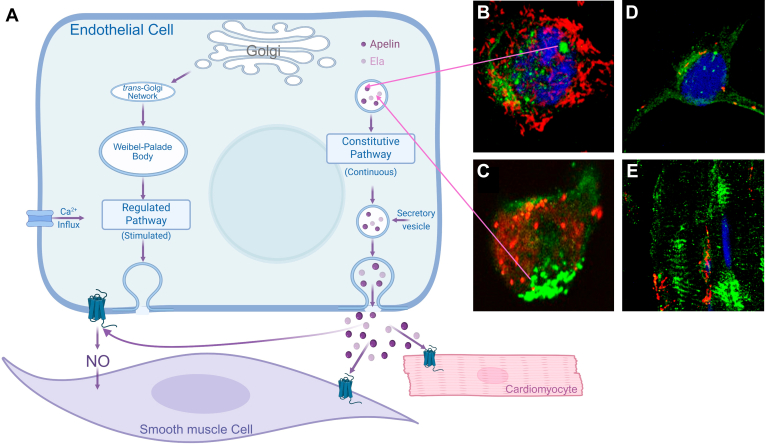
Localization of apelin, ELA and apelin receptor in the human cardiovascular system (A). Immunoreactive apelin (B) and ELA (C) localize to the small secretory vesicles of the constitutive pathway (green fluorescence) within the cytoplasm of endothelial cells. No colocalization was observed with von Willebrand Factor (red fluorescence), a specific marker of the larger Weibel-Palade bodies. Both peptides are thought to be released continuously from the constitutive pathway to bind to receptors present on endothelial cells (D, green fluorescence) in an autocrine or paracrine manner, to cause vasodilatation by the release of endothelium derived mediators such as nitric oxide (NO). Peptides released from endocardial endothelial cells within the heart are thought to mediate an increase in cardiac output, via receptors present on the surface cardiomyocytes (E), visualized (green fluorescence) in characteristic striated pattern. (Figure created in BioRender. Davenport, A. [2025] https://BioRender.com/n8xglgt and images adapted from Yang et al^13^ under a Creative Commons license CC BY 4.0.)

**Fig. 10:**
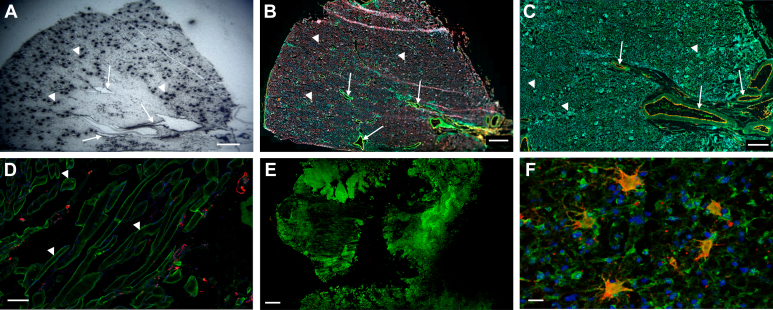
Examples showing the localization of apelin receptors in human tissues in emerging therapeutic targets. The kidney glomerulus has one of the highest densities of receptors in human peripheral tissues visualized by [^125^I]-[Pyr^1^]apelin-13 autoradiography (A, arrow heads) followed by the renal vasculature (arrows). In (B), receptors have been visualized using a green fluorescent labeled [Pyr^1^]apelin-13 analog (apelin488) and in (C) are visualized by an antibody to the receptor (green fluorescence). In (D), apelin receptor immunoreactivity (green fluorescence) localizes to the sarcolemma (arrowheads), the specialized plasma membrane surrounding individual contractile skeletal muscle fibers or myocytes. In (E), intense apelin receptor immunoreactivity localized to areas (palisades) within glioblastoma multiforme, a highly aggressive and most common brain cancer and an emerging target for antagonists. At higher magnification, a proportion of nonmalignant neurons and oligodendrocytes display receptor immunoreactivity (green fluorescence). Glial progenitor cells identified using a CD133 antibody (red fluorescence) colocalize with green receptor immunoreactivity, to give intense orange staining. Glial progenitor cells are a crucial subpopulation of cancer cells that drive tumor initiation and confer therapeutic resistance. (Scale bars: A–C, 2000 *μ*m; D, 250 *μ*m; E, 1000 *μ*m; and F, 10 *μ*m. Renal images adapted from Nyimanu et al,^129^ and glioblastoma from Williams et al,^28^ under a Creative Commons license CC BY 4.0.)

Significantly, receptor mRNA was most enriched in adipocytes, consistent with adipocytes activation improving insulin sensitivity (sections *Clinical pharmacology of endogenous apelin agonists*, *Potential first-in-class apelin receptor agonist as an exerkine mimetic for muscle and metabolic aging*, and *Diabetes and metabolic disorders*). Single cell profile^216^ (Fig. 8) shows the enrichment of receptor mRNA in endothelial cells (together with apelin mRNA) emphasizing the importance of this angiocrine signaling pathway. Enrichment is notable in adipocytes consistent with the role of apelin in adipocyte and metabolic regulation (section *Metabolic interventional studies*). Receptor mRNA was detectable as expected in cardiac muscle consistent with inotropic action on human isolated cardiomyocytes^5^ and increases in cardiac output in volunteers and patients in clinical studies (sections *Cardiovascular and cardiopulmonary interventional studies* and *heart failure*). Receptor mRNA was also localized to vascular smooth muscle cells in agreement with previous ligand binding studies and where removal of the endothelium unmasks a constrictor response in human isolated saphenous veins,^15^ although the vasodilator response predominates in vivo where the endothelium is intact (Fig. 9). Detection within skeletal muscle fibers supports the emerging role to increase glucose uptake mitochondrial biogenesis and nitric oxide production with presence of the protein confirmed by immunocytochemistry (Fig 10D, section *Potential first-in-class apelin receptor agonist as an exerkine mimetic for muscle and metabolic aging*).^217^ Interpretation of single cell data requires caution owing to several limitations including low-abundance transcripts, amplification bias and underrepresentation owing to cell fragility. Therefore, single-cell RNA sequencing can provide evidence supporting expression in a particular cell and potentially protein expression (ideally to be confirmed by immunocytochemistry), but not necessarily excluding expression. This is important for drug development to understand whether a particular type of cells is likely to respond to a therapeutic agent targeting the apelin receptor.

The majority of studies described below have used immunohistochemistry (unless otherwise stated) to localize apelin receptor protein to specific cells. In some cases, apelin peptide has also been localized with the objective of identifying changes in the signaling pathway caused by disease, to identify clinical conditions as potential targets for emerging drugs. These have focused on cardiovascular disease (PAH and HF) where apelin agonists are hypothesized to beneficially increase dilatation and cardiac output. In contrast, in a number of cancers, the receptors are expressed in tumors where antagonists are required to block detrimental actions of apelin.

#### 1 Cardiovascular

Apelin receptor density measured by ligand binding was significantly decreased in hearts of patients with dilated cardiomyopathy or ischemic heart disease, whereas apelin peptide levels measured by radioimmunoassay remained unchanged in normal hearts.^7^

#### 2 Renal

The kidney glomerulus has one of the highest densities of apelin receptors in human peripheral tissues. Nyimanu et al^129^ mapped in detail the precise cellular localization with antisera against the receptor, apelin and ELA in human kidney using multispectral fluorescent high content imaging and whole slide digitization (Fig. 10). The results showed a wide expression suggesting the apelin system could have diverse functional roles in regulating renal physiology especially cross-talk between the apelin system and the RAAS.

All 3 were present in renal blood vessels, colocalizing with the endothelial cell marker von Willebrand factor. Within the cortex, all 3 proteins colocalized to epithelial cells of the proximal convoluted tubules, identified by aquaporin 1. Interestingly, the receptor also colocalizes with sodium–glucose cotransporter 2 (SGLT2). The 3 proteins were present in epithelial cells in the descending thin limb, their presence while in the thick ascending limb, they colocalize with the sodium–potassium–chloride cotransporter. In the distal convoluted tubule, the apelin receptor overlays with the magnesium channel transient receptor potential melastatin 6. All 3 proteins are also found in the collecting duct, where aquaporin 4 marks the principal cells. Most importantly, the apelin receptor colocalizes with renin, in the juxtaglomerular apparatus, where activation of the RAAS occurs when low tubular sodium chloride is detected by the macula densa. Apelin may act within the juxtaglomerular apparatus to regulate RAAS activation with subsequent effects on blood pressure and glomerular filtration. This hypothesis is supported by the colocalization of the apelin receptor with sodium–potassium–chloride cotransporter within the loop of Henlé. The transport of sodium and chloride by this cotransporter at the macula densa acts as the first step in tubuloglomerular feedback in signaling renin response. This study provided the rational for clinical studies using in patients with renal failure (section *Renal and fluid homeostasis*).^66^^,^^218^

#### 3 Skeletal muscle

Dray et al^219^ measured receptor and peptide expression by polymerase chain reaction (PCR) in adipose tissue and skeletal muscle in control subjects and patients with type 2 diabetic during a hyperinsulinemic-euglycemic clamp. Insulin stimulation increased both peptide and receptor in adipose tissue of controls but not in diabetics. In muscle, only receptor mRNA levels increased in both groups. These findings suggest that regulation of the apelin and its receptor varies by tissue type and insulin resistance severity.^219^ Localization of the apelin receptor to the sarcolemma of skeletal muscle is shown in Fig. 10D.

#### 4 Pancreas

The study by Ringstrom et al^220^ demonstrates that apelin is expressed within pancreatic islets, mainly in *β*-cells and *α*-cells, and the receptor is also present in these cells. Apelin-36 had a dose-dependent effect on insulin secretion: low physiological concentrations strongly inhibited insulin release, whereas a high concentration caused a modest increase in glucose-stimulated insulin secretion. Apelin expression was upregulated in *β*-cells of type 2 diabetic animal models but not influenced by glucose in human islets, although glucocorticoids significantly reduce apelin expression. The study suggested that apelin is a novel islet-derived peptide that regulates insulin secretion and acting in an autocrine/paracrine manner within the islet.

#### 5 Liver

In control liver immunocytochemistry demonstrated receptor staining in cholangiocytes (epithelial cells of the bile duct) but minimal staining in hepatocytes. However, a significant increase in receptor gene expression was measured by PCR in cholangiocarcinoma.^221^ Hepatocellular carcinoma primarily involves proliferation of hepatocytes. In patients with tumors with high-expression of the receptor detected by immunocytochemistry had a shorter recurrence-free survival and overall survival than the low-expression group.^222^

#### 6 Central nervous system

In cerebral cortex of normal brain tissue, using the same technique of fluorescent high content imaging, Williams et al,^28^ demonstrated the apelin receptor was expressed in endothelial cells of cerebral vessels (Fig. 7). Importantly, the vasculature of glioblastoma multiforme, the most common human brain tumor the signal was upregulated. The probability of survival in patients is reduced with high levels of apelin receptor mRNA is these tumors (Human Protein Atlas)^215^ and this condition has emerged as a potential target for apelin receptor antagonists such as MM54.^223^ Apelin receptor, together with both peptide ligands were visualized in progenitor glioblastoma stem cell lineages in the microenvironment driving tumor recurrence and therapeutic resistance: neural-progenitor-like cells, oligodendrocyte-progenitor-like cells, and mesenchymal-like cells, although interestingly ELA was not detected in reactive astrocytic cells.^224^

## VI Endogenous peptide distribution

In the single-cell profile^216^ mRNA was most enriched as expected in endothelial cells (Fig. 8) and interestingly in cardiomyocytes, with detectable level in smooth muscle, glial and epithelial cells but below the level for detection in adipocytes.

### A Distribution of apelin mRNA and protein in human tissues

Read et al^53^ summarized results for the distribution of apelin mRNA and concluded there was a similar wide distribution as its receptor in human brain and peripheral tissues. Highest levels of preproapelin mRNA (using RT-PCR), were detected in the in spinal cord followed by all regions of the human brain examined including the corpus callosum, amygdala, substantia nigra, and pituitary gland. In the periphery highest levels were observed in the placenta.^12^ Expression data using bulk RNA-sequencing (Fig. 7) also detected apelin peptide mRNA in 50 healthy human tissues with enrichment in brain and placenta, in agreement with Medhurst et al.^12^ Lower levels were detected adipocytes suggesting the peptide may function in an autocrine or paracrine manner improving insulin sensitivity (see sections *Clinical pharmacology of endogenous apelin agonists*, *Potential first-in-class apelin receptor agonist as an exerkine mimetic for muscle and Metabolic aging*, and *diabetes and metabolic disorders*). There was general concordance between peptide and receptor mRNA, except for the spleen where apelin mRNA was particularly abundant (Fig. 7). mRNA was low or below the level for detection in heart, skeletal and smooth muscle.

### B Distribution of the apelin mRNA and protein in single cells

#### 1 Cardiovascular

The peptide was detected using immunocytochemistry in vascular endothelial cells lining blood vessels (arteries, veins, and small resistance vessels) in the human heart, kidney, and adrenal gland. In the heart, localization to endocardial endothelial cells is consistent with its positive inotropic effects when released on underlying cardiomyocytes.^225^ In agreement, apelin-like immunoreactivity measured by radioimmunoassay was detected in all human cardiac and vascular tissues tested (atria, ventricles, saphenous vein, coronary, and internal mammary arteries). Apelin was restricted to the endothelium of normal coronary artery but localized by immunohistochemistry to smooth muscle cells and infiltrating macrophages within the atherosclerotic plaque.^7^ The signaling pathway measured by PCR was upregulated in human aortic valve stenosis with both receptor and peptide significantly increased. Using immunohistochemistry, apelin localized to the valvular endothelial layer of the diseased valve, endothelial cells in neo vessels, fibroblasts, and macrophages adjacent to vessels in the stromal area.^226^

#### 2 Liver

Immunohistochemical staining showed apelin was mainly localized in the perivenular areas of control liver tissue and lower levels in hepatic stellate cells (primarily regulating liver fibrosis) and hepatocytes. In livers from patient transplanted for disease intense staining was detected in these areas with significant correlations between apelin mRNA level and liver fibrosis and grade of esophageal varices.^227^

#### 3 Breast

Cytoplasmic apelin immunoreactivity was detected in the ductal and lobular epithelial cells and vascular endothelial cells of the normal breast tissue.^228^ The malignant tumor cells of invasive ductal or lobular carcinoma also expressed similar levels of immunoreactive apelin.^228^

#### 4 Gastrointestinal tract

Apelin immunoreactivity increased in epithelial cells of the colon in patients with ulcerative colitis and Crohn disease compared with normal colonic mucosa where only low levels of immunostaining could be detected in the epithelium.^229^

#### 5 Placenta

As previously noted, mRNA encoding peptide and receptor (Fig. 8) is most abundant. In the first trimester of gestation, apelin was localized to cytotrophoblast cells (that contribute to embryo implantation and maternal-fetal exchange) forming the inner proliferative layer of placental villi, the stroma of placental villi, with weak staining in syncytiotrophoblast cells. Interestingly, in the third trimester, levels decreased, whereas there was a strong increase of apelin expression in preeclamptic placentas.

Immunoreactive apelin was also present in the endothelial lining of blood capillaries and placental artery with receptor staining in epithelial cells of vesicles and mesenchymal cells of the Wharton jelly—specialized connective tissue protecting blood vessels of the umbilical cord.^230^

### C Distribution of ELA mRNA and protein in human tissues

Expression data using bulk RNA-sequencing (Fig. 7) suggested that *APELA* mRNA encoding ELA peptide had a more restricted distribution and was barely detectable in only 21 tissues compared with *APLN,* with enrichment in the kidney and prostate. ELA signaling is essential for early cardiovascular development in the embryonic stage^8^^,^^101^ acting as a motogen.^9^ These low levels may reflect the peptide is more important in early development than in adult humans. Alternatively, mRNA in both bulk and single cells may be underestimated.

### D Distribution of ELA mRNA and protein in single cells

In agreement with the bulk data RNA, single cell enrichment was observed in the proximal tubular cells of the kidney and prostatic glandular epithelial cells. Intriguingly mRNA also localized to cytotrophoblasts considered stem cells for other trophoblast cell types including syncytiotrophoblast, a specialized, multinucleated cell layer in the placenta.

#### 1 Cardiovascular

Yang et al,^13^ measured ELA mRNA expression in human cardiovascular tissues and plasma using real-time quantitative polymerase chain reaction combined with dual-labeling immunofluorescent staining, and immunoassays to visualize the peptide. Expression of *APELA* transcript was observed in all human blood vessels investigated (coronary, mammary artery, and pulmonary artery, aorta, umbilical, and saphenous veins) as well as of the heart (left ventricle) and lungs. There was a trend for *APELA* expression to be higher in arteries than veins. ELA immunoreactivity was localized and restricted to endothelial cells in all of these vessels. Levels of ELA measured in healthy human plasma (0.34 nM) were comparable to apelin (0.26 nM). These low levels are consistent with an endothelium derived peptide, locally released from the secretory pathway. Similar to apelin, ELA expression was reduced in cardiopulmonary tissues from patients with PAH and animal models.^13^ Nyimanu et al^231^ noted the ex vivo plasma half-life of ELA was ∼47 minutes. They speculated that the intensely charged nature of ELA-32 peptide was binding to plasma proteins to evade proteolytic degradation.

The in vitro plasma half-life of ELA-32 observed in this study was ∼47 minutes. This was consistent with pilot studies where peptide recovery from samples extracted in the absence of chaotropes was relatively low compared with when guanidine hydrochloride was present. Given the intensely charged nature of ELA-32 peptide, a plausible reason could be that the peptide was binding to plasma proteins to evade proteolytic degradation, but more studies are required to confirm this observation. Measurement of ELA by ELISA was validated by mass spectrometry. However, the endogenous peptide could not be measured directly by mass spectrometry as a result of the unusual physicochemical properties of these peptides, which are highly charged because of multiple arginine and lysine residues.

#### 2 Renal

See section *Renal*.

#### 3 Placenta

In placenta from normal pregnant women both ELA and apelin receptors are highly expressed in syncytiotrophoblasts and moderately expressed in cytotrophoblasts (consistent will the single cell mRNA expression but lower in the placental villi of late-onset preeclampsia.^164^

## VII Synthetic agonists

To explore the therapeutic potential of apelin agonists more fully requires the design or discovery of apelin peptide and small molecule agonists with longer plasma half-lives than the endogenous peptides and improved pharmacokinetics. Strategies to achieve these improvements for peptides include design of analogs that are more resistant to degradation^19^, ^20^, ^21^, achieved through introduction of unnatural amino acids,^19^ cyclization,^16^^,^^26^^,^^27^ lipidation, glycosylation, PEGylation,^25^ and conjugation to antiserum albumin antibodies.^22^ Small-molecule agonists of the apelin receptor were initially identified in high throughput screens of compound libraries^29^^,^^30^ with subsequent structure activity programs to refine pharmacodynamic and pharmacokinetic profiles.^31^, ^32^, ^33^, ^34^, ^35^, ^36^, ^37^, ^38^, ^39^, ^40^, ^41^, ^42^, ^43^^,^^50^ There is also increasing interest in the development of therapeutic antibodies agonists.^23^

Eurofins DiscoverRx offers a comprehensive suite of cell-based assays (catalogued as *AGTRL1*) to measure activity of apelin ligands. These include *β*-arrestin assays designed to study signaling, particularly through G protein-independent pathways as well as receptor internalization. These assays are widely used in combination with a third assay to measure inhibition of cAMP to identify ligands that can preferentially activate G protein versus *β*-arrestin pathways to measure bias. The assays can also be used to identify antagonist activity and measure K_B_ values.^232^

### A Clinical pharmacology of endogenous apelin agonists

#### 1 Safety of apelin in investigational clinical studies

[Pyr^1^]apelin-13 has been used as a challenge compound in a small number of investigational clinical studies (Table 2). The peptide has been administered by intrabrachial infusions in forearm blood flow studies with dose ranges of 0.3–100 nmol/min over 6 minutes.^25^^,^^57^^,^^59^ In systemic studies, higher doses have been tested (30–300 nmol/min administered over 5–10 minutes for each dose)^57^^,^^59^^,^^65^ as well as prolonged infusion over 6 hours (30 nmol/min).^59^ Systemically, maximally effective doses were achieved after 30 or 100 nmol/min apelin with a reduction in response generally observed with the higher dose of 300 nmol/min. For the prolonged infusion the (sub)maximal dose of 30 nmol/min was used with significant effects seen over the whole infusion period compared with placebo control in both healthy volunteers and patients with HF.^59^ These data suggest that the highest dose of apelin, 300 nmol/min resulted in tachyphylaxis within a few minutes of administration likely a consequence of receptor desensitization that was not seen with the continuous infusion of the lower dose, 30 nmol/min, even after 6 hours. Apelin has also been tested in PAH,^62^ type 2 diabetes^63^ and CKD.^66^

Apelin-36 (that has a longer half-life than [Pyr^1^]apelin-13) has also been tested in 2 studies^56^^,^^57^ as well as the principal active metabolite, [Pyr^1^]apelin-13_(1-12)_^60^ and the biased agonist, MM07.^26^ In all studies (Table 2) apelin and related agonists were reported to be well tolerated without adverse side effects, including high dose systemic infusions, where there was little or no effect on heart rate. Similar to many other endogenous peptides, the plasma [Pyr^1^]apelin-13 half-life is only a few minutes and will be removed from the circulation with 15 to 20 minutes. From these studies an optimal dose range has emerged, of between 1 and 30 nmol/min. To date, no clinical studies on ELA peptides have been reported.

#### 2 Cardiovascular and cardiopulmonary interventional studies

The major actions in humans in the forearm or systemically are endothelium-dependent vasodilation in both peripheral and coronary arteries mediated by nitric oxide and is preserved even in patients with chronic HF. Apelin lowers mean arterial pressure and peripheral vascular resistance, making it a potential antihypertensive agent. Systemic infusion of apelin increases cardiac index and left ventricular contractility, while reducing left ventricular end-diastolic pressure, suggesting a beneficial inotropic effect. These results support the development of apelin receptor agonists as a novel class of HF drugs. Unlike current treatments, they may offer a unique mechanism to enhance cardiac output and reduce vascular resistance without adverse effects on heart rate.

Japp and Newby,^56^ reported the first in human studies using apelin and discovered the peptide plays a significant role in vascular homeostasis. Forearm blood flow was measured by venous occlusion plethysmography in healthy volunteers (*n* = 24) following intrabrachial infusions of [Pyr^1^]apelin-13 (0.1–30 nmol/min). Vasodilation was attenuated by a nitric oxide inhibitor but not by cyclooxygenase inhibition, excluding thromboxane mediated vasodilation. Importantly, the vasodilatory response in the forearm to acute intrabrachial infusions of [Pyr^1^]apelin-13 was retained in patients with chronic HF (*n* = 18), despite reduced responses to infused acetylcholine releasing nitric oxide.^57^ Systemic infusions of [Pyr^1^]apelin-13 (30–300 nmol/min) increased cardiac index, lowered mean arterial pressure and peripheral vascular resistance in patients without increased heart rate. An intracoronary bolus of apelin-36 (*n* = 6) increased coronary blood flow, the maximum rate of rise in left ventricular pressure and reduced peak and end-diastolic left ventricular pressures.^57^

Significant interactions between the apelin signaling pathway and the renin-angiotensin system (RAS) have previously been reported. Apelin is a functional antagonist opposing the vasoconstrictor, water and salt retention actions of ANG II, to cause vasodilation, lower blood pressure, promote diuresis to mediate cardioprotection.^84^ A study involving 48 volunteers and 12 patients with HF tested the effects of [Pyr^1^]apelin-13 on cardiovascular function using different methods of local and systemic activation of RAS. This included ANG II infusions to increase local and systemic plasma levels and application of sodium-deplete diet that more than doubled the plasma ANG II concentrations. [Pyr^1^]apelin-13 alone consistently increased cardiac index as a measure of heart performance in both healthy individuals and patients with chronic HF. This suggests it can effectively boost heart function *without increasing heart rate*, which is a key goal in HF therapy. The peptide lowered mean arterial pressure and peripheral vascular resistance, indicating improved blood flow and reduced workload on the heart. This is especially beneficial in HF, where the heart struggles to pump against high resistance. The beneficial effects were maintained even when the RAS was activated (indirectly through sodium depletion, sensed by the macula densa leading to increase renin release or directly through receptor activation by angiotensin II infusion). Importantly, 6-hour infusion in patients with HF led to sustained improvements in cardiac index and left ventricular ejection fraction.^59^

The beneficial vasodilation in patients with chronic HF after infusion of [Pyr^1^]apelin-13 (1, 10, 100 nmol/min for 6 minutes) was replicated in the healthy volunteers (*n* = 12) dose-dependent increase in forearm blood flow. The G protein biased agonist MM07 was more efficacious as hypothesized and produced a significant flow with a maximum dilatation double that as seen with the endogenous peptide. In addition, repeated doses of MM07 (100 nmol/min) produced reproducible increases in forearm blood flow, consistent with no desensitization of the receptor. Although vasoconstriction by [Pyr^1^]apelin-13 in isolated human vessels in vitro has been reported where the endothelium was removed, no vasoconstriction was observed in hand vein ([Pyr^1^]apelin-13, 100 nmol/min and MM07 at 10 and 100 nmol/min), both peptides reversed an established norepinephrine constrictor response and significantly increased venous flow.^26^ This can be explained as the concentration of apelin used was higher than Japp and Newby^56^ who reported no reversal of the norepinephrine constriction in their study. The results suggested a G protein biased agonist at the apelin receptor preferentially stimulates the G-protein pathway, which could translate to improved efficacy in the clinic by selectively stimulating vasodilatation and inotropic actions but avoiding activating detrimental *β*-arrestin-dependent pathways.

In patients with chronic obstructive pulmonary disease with PH (PAH) there was a significant beneficial increase in cardiac index as well as forearm and peripheral blood flow with infusions of [Pyr^1^]apelin-13. The vascular responses were smaller as expected, consistent with a reduced nitric oxide release in these patients.^26^ (Brame and Davenport, unpublished) and supporting the results of Japp and Newby,^56^ blocking apelin vasodilation with nitric oxide inhibitors.

PAH is currently considered one of the most promising therapeutic targets for apelin receptor agonists, based on efficacy in animal models, particularly those that are G protein biased. MM07, was as effective as macitentan (a standard of care endothelin receptor antagonist) in reversing PAH symptoms in a rat model^233^ (sections *Clinical pharmacology of the first G protein-biased apelin agonist, MM07* and *pulmonary hypertension*).

Brash et al,^62^ determined the short-term pulmonary hemodynamic effects of systemic [Pyr^1^]apelin-13 apelin infusion in patients with idiopathic PAH, where up to 40% can have a mutation in variants in bone morphogenic protein type 2 receptor that is associated with a reduction in both apelin and ELA peptides without changes in the receptor.^13^ Nineteen patients with PAH received intravenous peptide over 5 minutes for each dose and matched saline placebo during invasive right heart catheterization. There was a beneficial reduction in pulmonary vascular resistance and increased cardiac output with a maximal effect at 30 nmol/min. Heart rate was not altered. These actions were enhanced in a subgroup on an established treatment with phosphodiesterase-5 inhibitors, consistent with apelin actions being mediated via nitric oxide. The study provides crucial evidence albeit in an acute study for therapeutic strategy coadministering apelin agonists with a widely prescribed standard of care phosphodiesterase-5 inhibitor.

#### 3 Metabolic interventional studies

Preclinical insulin clamping experiments in mice suggested [Pyr^1^]apelin-13 had a beneficial insulin-sensitizing action.^234^ In this technique plasma glucose is kept constant, so that any changes in glucose uptake can be attributed to the effect of the drug being tested, in this case apelin, not to fluctuations in glucose levels. This concept was shown to translate to human by Gourdy et al.^63^ [Pyr^1^]apelin-13 administered systemically through intravenous continuous infusion at doses of 9 and 30 nmol/kg and at a rate of 75 and 250 pmol.kg^−1^.min^−1^, respectively during 2 hours, increased insulin sensitivity by increasing glucose uptake of skeletal muscles and adipose tissue in overweight humans.^63^

The mechanism of action has been investigated with, for example, a role for apelin-induced phosphorylated AMPK, Akt and endothelial nitric oxide synthase (eNOS) linked to muscle glucose uptake established.^99^^,^^234^ In diabetes, where high glucose levels are chronic, endothelial dysfunction can occur, potentially impairing the vasodilation process and contributing to vascular complications. By increasing insulin sensitivity in insulin-resistant subjects, apelin may stimulate microvascular perfusion, increasing blood flow in skeletal muscle and subcutaneous adipose tissue.

Schinzari et al^64^ investigated the effect of [Pyr^1^]apelin-13 in various group of patients (*n* = 48) with central obesity that often exhibit impaired insulin-stimulated vasodilation and increased vasoconstriction because of elevated endothelin-1 (ET-1) activity. These vascular abnormalities contribute to insulin resistance and cardiovascular risk. During hyperinsulinemia, apelin (1.5 nmol/min infused into the forearm for 30 minutes) enhanced both endothelium-dependent and independent vasodilation, suggesting it amplifies the vascular effects of insulin. The peptide also blunted vasoconstriction mediated by ANG II (via angiotensin 1 receptors [AT_1_]) and ET-1 (via endothelin A receptors), but this effect was independent of nitric oxide.

As proof of concept, the cardiovascular system in individuals with increased BMI responded to apelin. [Pyr^1^]apelin-13 infused systemically at a dose 30 nmol/min for 2 hours into participants with increased BMI or type 2 diabetes caused a significant rise in cardiac index and stroke volume index, whilst reducing peripheral vascular resistance compared with saline control. Mean arterial pressure was also reduced with increased BMI.

These studies suggest in addition to improve vascular insulin sensitivity, potentially enhancing glucose uptake in insulin-resistant tissues, apelin reduces vasoconstriction, lowering cardiovascular risk. However, glucagon-like peptide-1 (GLP-1) receptor agonists (eg, semaglutide and tirzepatide) and SGLT2 inhibitors (eg, empagliflozin) acting via complementary mechanisms have similar protective actions on the cardiovascular and renal systems and a role of apelin agonists seems less likely.

#### 4 Renal interventional studies

CKD affects ∼10% of the population and is strongly linked to cardiovascular disease, which worsens patient outcomes. Chapman et al,^66^ (section *Renal and fluid homeostasis*) demonstrated [Pyr^1^]apelin-13 reduced mean arterial pressure, systemic vascular resistance and increased cardiac index as expected in both healthy controls (*n* = 12) and matched patients with CKD. However, in CKD, there was also a reduced glomerular filtration rate (∼10%) and proteinuria was clinically significant (∼25%), a marker of kidney damage and a predictor of disease progression. The study suggests a long acting apelin agonist may reduce blood pressure and vascular resistance while increasing cardiac output, reducing cardiovascular strain, potentially lowering the risk of heart-related complications. Lower proteinuria may indicate reduced glomerular stress and inflammation, potentially slowing CKD progression. Increased renal blood flow and natriuresis suggest better kidney perfusion and sodium handling, which can help manage fluid overload. Combining all of these properties has the potential to be disease modifying.

Hyponatremia (elevated plasma sodium levels) affects up to 30% in hospitalized patients with the most common outcome, syndrome of inappropriate antidiuresis (SIAD). Intravenous [Pyr^1^]apelin-13 is being evaluated in healthy participants with artificially induced SIAD to test whether urinary excretion and therefore sodium excretion is increased (Table 2, NCT06277336).

### B Pharmacology of apelin peptide and antibody agonists

Apelin peptides have a short half-life of ∼5 to 8 minutes in plasma^52^^,^^124^ being cleaved by circulating enzymes that include ACE2. ACE2 was reported to partially inactivate apelin-13 and apelin-17^121^^,^^122^^,^^235^ although a later study found the [Pyr^1^]apelin-13_(1-12)_ cleavage product to be biologically active, expressed in human cardiovascular tissues and to produce dose-dependent decreases in blood pressure in the anesthetized rat and in human forearm blood flow.^60^ Modifications to apelin peptides of varying length, such as termini capping, cyclization, lipidation, glycosylation, or stapling can extend half-life and improve oral bioavailability^236^ (see Table 1).

#### 1 Apelin-12 analogs

Apelin-12 retains the biological activity of the endogenous apelin isoforms. Three cyclic analog of apelin-12, cycloapelin-12_(1-12)_, cyclourea apelin-12 _(1-7)_ and cycloapelin-12_(1-7)_ that retain agonist activity,^16^ have informed the development of more drug-like apelin peptides including MM07,^26^ a G protein-biased, cyclized apelin ligand that was designed to increase peptide half-life by protecting against peptidases (see section *Clinical pharmacology of the first G protein-biased apelin agonist, MM07*). Alternative strategies have involved conjugating a lipid to apelin-12 to form a lipopeptide, Palmitate-VTLPLWATYTYR, which retains high affinity for the apelin receptor but has an extended duration of action and increased intracellular access.^17^

#### 2 Apelin-13 analogs

There has been extensive structure activity exploration around the apelin-13 sequence (see also Davenport et al^47^). Substitution of the C terminal phenylalanine of apelin-13 or [Pyr^1^]apelin-13 by alanine results in the modified peptide F13A. F13A was initially reported to lack agonist activity at the artificially expressed rat apelin receptor in vitro^237^ and the effects of apelin-13 on blood pressure in vivo were antagonized by coadministration of F13A.^238^ However, F13A was shown to have comparable activity to apelin-13 and [Pyr^1^]apelin-13 in alanine scanning of these peptides in binding, signaling (Ca^2+^ and cAMP) and receptor internalization assays.^11^^,^^12^ More recently, F13A was shown to compete for [Glp^65^, Nle^75^, Tyr^77^][^125^I]apelin-13 with subnanomolar affinity in human heart, inhibit forskolin stimulated cAMP and contract human saphenous vein with comparable potency to [Pyr^1^]apelin-13.^13^ However, despite these reports F13A is widely used as an apelin receptor antagonist and although frequently described as a competitive antagonist, Schild analysis and antagonist affinity values for F13A are not available. Therefore, it may be that the peptide is a partial agonist whose pharmacological effects (either agonist or antagonist) are assay and context dependent. For future studies it is advised that F13A be rigorously characterized in appropriate control experiments and ideally studies repeated using a second apelin antagonist to confirm results.

The impact of cyclisation of apelin-13 analogs on stability and pharmacological profile has also been explored.^239^ With the aim of increasing resistance to ACE2 cleavage and thus improved pharmacokinetics as well as increased receptor affinity and signaling, incorporation of unnatural amino acids substituting for Pro12 and/or Phe13 in the C-terminus resulted in lead compounds **47** and **53**, which are 2 of the most potent apelin analogs to date with dissociation affinity constants of 80 pM.^18^ The addition of an antiserum albumin domain antibody (AlbudAb) via a [PEG4] linker to a modified apelin peptide incorporating a methionine to norleucine substitution at position 11 and an phenylalanine to 3,4,5-trifluorophenylalanine at position 13 resulted in identification of AlbudAb-MM202.^22^ This peptide binds to human apelin receptor with similar affinity to the endogenous peptides [Pyr^1^]apelin-13 and apelin-17; is active in G protein and *β*-arrestin assays; binds with high affinity to immobilized human serum albumin; and induces hemodynamic and cardiovascular effects in rats providing proof-of-concept for conjugation of AlbudAb to peptide ligands as a potential novel therapeutic strategy.

#### 3 Apelin-17 analogs

Metabolically stable apelin-17 analogs have also been designed including LIT01-196 that contains a fluorocarbon chain attached to the N-terminus of apelin-17.^240^ This modification does not detrimentally affect apelin receptor affinity or in vitro biological activity, including nitric oxide-dependent vasorelaxation, of LIT01-196 compared with the parent molecule. However, it does markedly extend the plasma half-life to over 24 hours compared with <5 minutes for apelin-17. Intracerebroventricular administration of LIT01-196 significantly reduced systemic vasopressin release in response to water deprivation in mice with 2 orders of magnitude more potency that apelin-17. Intravenous LIT01-196 increased urine output and decreased arterial blood pressure in normotensive rats, which was sustained for >100 minutes. LIT01-196 has subsequently been shown to decrease vasopressin antidiuretic effects and improve hyponatremia in a rat model,^19^ normalize arterial blood pressure in conscious hypertensive deoxycorticosterone acetate-salt rats over a 7-hour window.^20^ and have beneficial effects in a model of HF after myocardial infarction (MI).^241^

Apelin-17 is now known to be a target of the human plasma kallikrein enzyme, which cleaves the 3 N-terminal (KFR) amino acids of the peptide, exposing an R amino acid at the N-terminus and resulting in significantly reduced biological activity and abolished blood pressure lowering effects.^119^ Addition of palmitic acid or polyethylene glycol (PEG) to the N-terminus of apelin-17 protects against kallikrein cleavage whilst retaining good pharmacological activity at the apelin receptor, extends plasma half-life, and restores the blood pressure lowering effects of the peptide. A subsequent study also showed that the incorporation of nonnatural cyclohexylalanine and homoarginine amino acids extended the plasma half-life of [Pyr^1^]apelin-13 and apelin-17 by 40-fold and 340-fold, respectively, with the modified apelin-17 peptide showing pronounced blood pressure lowering effects.^21^ Reducing peptidase metabolism of apelin-17 to extend the plasma half-life may therefore be a novel therapeutic strategy to treat diseases characterized by inappropriate antidiuresis.

#### 4 Apelin-36 analogs

The conjugation of a 40-kDa PEG moiety to the N-terminus of apelin-36 was shown to enhance the duration of action on increased cardiac ejection fraction versus unmodified apelin-36 after intravenous infusion in rats.^242^ PEG moiety to the N-terminus of apelin-36 showed comparable binding affinity and G protein activation to apelin-36 in in vitro assays. After development of a series of apelin-36 variants, modified apelin-36(L28A) incorporating an L28A substitution, and apelin-36-[L28C(30kDa-PEG)] incorporating an L28C substitution and conjugation of a 30-kDa PEG linker to the Cys side-chain, were shown to significantly lower blood glucose and improve glucose tolerance in obese rats but had no effect on blood pressure.^243^ The authors concluded that the peptides function independently of the apelin receptor. Further assessment, however, revealed that both apelin-36(L28A) and apelin-36-[L28C(30 kDa-PEG)] bind to rat and human apelin receptor with similar affinities to apelin-36, and are active in G protein assays, but show less potency in *β*-arrestin assays.^244^ The peptides therefore show some G protein versus *β*-arrestin bias, at least in in vitro assays.

#### 5 Pharmacology of single domain antibodies

There is growing interest in the generation of antibodies or nanobodies with agonist activity at GPCRs that possess improved selectivity and pharmacokinetic profiles compared with peptide and small molecule drugs. Existing high throughput technologies such as phage display can be used to isolate receptor-specific antibodies, however, there are challenges associated with discovery of functional antibodies targeting GPCRs owing to their conformational flexibility, complex signaling pathways and the inherent low immunogenicity of these integral membrane proteins. Camelid-derived single domain antibodies for the apelin receptor have been generated using a nanodisc/proteoliposome system and shown to have competitive, orthosteric antagonistic but not agonistic properties.^23^ Cocrystallization of one of these single-domain antibodies, JN241 (K_d_ 83pM), revealed in cryo-electron microscopic studies binding of the molecule to both the second extracellular loop and importantly deep insertion of the CDR3 into the orthosteric binding pocket of the apelin receptor, with R128^4.64^ and Y264^6.51^ important for interactions with residue E104 of JN241. Having determined those residues to be critical for the antagonist activity of JN241, modification of the CDR3 by insertion of a tyrosine was shown to produce an agonistic single-domain antibody, JN241-9.^23^ Looking forward, a novel function-based high-throughput screen has been validated using the apelin receptor as a GPCR target, which additionally identified apelin receptor agonistic antibodies (JN300) as well as the neutral binders and antagonists isolated using phage display.^245^ This may herald a new phase for discovery of agonistic antibody drugs.

### C Pathway biased apelin agonists: peptides

#### 1 Rationale for G protein-biased agonist to optimize therapeutic action

A major limitation of many current medicines that act as agonists at GPCRs is that after stimulating G protein pathways, to elicit a physiological action, the *β*-arrestin pathway is also activated resulting in receptor internalization and silencing, limiting the response.^246^^,^^247^ Activation by apelin of the G_*α*i_-protein pathway elicits a beneficial inotropic action and increase in cardiac output in the heart. However, in cardiovascular disease such as PAH (eg, Yang et al^13^; sections *Clinical pharmacology of the first G protein-biased apelin agonist, MM07* and *Pulmonary hypertension*), plasma levels of the endogenous peptides are lower. Under these conditions stretch signals, sensed by the apelin receptor, result in signaling via *β*-arrestins, causing detrimental cardiac hypertrophy.^172^

The therapeutic rationale for apelin agonists biased to the G protein pathway that have reduced or no effect on *β*-arrestin recruitment is that receptor desensitization and tolerance to the drug does not develop through loss of the receptor at the cell surface in response to repeated drug administration. Secondly, where endogenous peptide levels are reduced, beneficial G protein-mediated actions are maintained avoiding deleterious *β*-arrestins stimulating cardiac hypertrophy and therefore reducing “on-target” side effects. Structural biology has now shown that ligands can stabilize receptor conformations such that just one of these pathways may be preferentially activated. As proof of concept, oliceridine, a G protein biased *μ* opioid receptor agonist has been approved by the US Food and Drug Administration for the management of acute pain with reduced respiratory depression and gastrointestinal side effects that currently limit unbiased agonists.^248^

Quantifying ligand bias in vitro through the calculation of ratios of transduction coefficients (*ΔΔ*log(τ/KA)) is preferred to comparisons of relative potency of test ligands determined in different pathway assays using a reference ligand. For the studies discussed in this section the reference agonist is generally [Pyr^1^]apelin-13. This has the advantage of being the predominant physiological agonist and is a relatively balanced agonist in G protein and *β*-arrestin assays allowing simultaneous estimations of physiological and pathway bias for test ligands. In practice pathway bias can be determined by comparison with the reference ligand, [Pyr^1^]apelin-13, to generate bias factors for the test ligand from calculated *ΔΔ*log_10_(τ/K_A_) values across G protein compared with *β*-arrestin signaling assays (see Davenport et al,^236^ for further details of this analysis).

#### 2 Identification of G protein-biased apelin analogs

K16P (apelin-17(1-16)), a modified analog of apelin-17 lacking the C-terminal F, is described as G protein biased (bias factors calculated as 10^ΔΔlog10(τ/KA)^ with apelin-17 as the reference ligand was 8.9 for G*α*i vs *β*-arrestin-2) but did not decrease blood pressure unlike apelin-17.^249^ The authors conclude that apelin receptor mediated reductions in blood pressure are *β*-arrestin dependent, although this conflicts with the subsequent findings observed for other G protein biased apelin peptides (see below). One explanation might be that K16P and apelin-17 vasoactivity was investigated in rat glomerular afferent arterioles preconstricted with ANG II at a single concentration (300 nM), a dose-response study is required to confirm that K16P, a reported G protein biased agonist, lacks vasodilator action.

Generation of a novel series of macrocyclic apelin-13 analog led to the identification of 4 G*α*_i1/oA_ protein biased peptides with nanomolar affinity for the apelin receptor.^47^^,^^250^ This description is qualitative as bias factors were not calculated for these compounds compared with apelin-13 from the available assay data. Macrocyclic apelin-13 analogs with reduced molecular weight were subsequently designed and profiled pharmacologically including compound **39** that had comparable potency in G*α*_i1_ and *β*-arrestin 2 assays but little or no effect in a G*α*_12_ assay. Whereas compound **40** showed improved G*α*_12_ potency compared with apelin-13 with similar effects in the other assays. In this study bias factors (10^ΔΔlog10(τ/KA)^) were calculated to confirm signaling bias. Both of these analogs had long half-lives in rat plasma in vitro (5.8 and >24 hours, respectively compared with 0.4 hour for apelin-13) and prolonged beneficial cardiovascular actions in rats in vivo.^27^ Structure-activity relationships (SAR) around the C terminus of [Pyr^1^]apelin-13 with substitutions at the N*α*-amid bond between Pro^12^ and Phe^13^ by alkyl groups and/or introduction of positively charged unnatural amino acids has been performed.^251^ Plasma half-life was enhanced with bulky additions such as *N*-(3′PhO)Bn (compound **40**; in vitro t_1/2_ >24 hours in rat plasma) whilst retaining nanomolar affinity for the receptor.

As part of a study that used naturally occurring apelin receptor variants to compare binding modes of small molecule versus peptides at the apelin receptor^28^ (see section X-ray crystallography and cryo-electron microscopy) a series of apelin peptide agonists was reported (structures not disclosed) that included balanced and completely G protein biased agonists. Lead compounds were tested in vivo with both NXE’515 (balanced agonist) and NXE’065 (G protein biased as lacked any activity in *β*-arrestin assays up to 10 *μ*M) exhibiting similar cardiovascular profiles to [Pyr^1^]apelin-13 when administered to anesthetized rats; decreasing left ventricular systolic pressure, increasing cardiac output and decreasing peripheral arterial pressure indicating that the beneficial cardiovascular actions of apelin agonists are likely via G protein signaling pathways.^28^

#### 3 CLR325

CLR325 is a synthetic cyclic peptide agonist analog of apelin-13 (structure not disclosed) developed by Novartis Pharmaceuticals. To date, no preclinical data for CLR325 have been published. The safety and tolerability of CLR325 was tested in a phase 2 trial after intravenous infusion into 26 patients with chronic stable HF, divided into 3 study groups. Three doses of CLR325 were tested; 0.25, 2.5, and 8 mg/kg/min. The plasma half-life was comparatively short ranging from 1.9 to 3 hours and the volume of distribution at steady state was 28 L, consistent with the peptide confined to the plasma compartment.^67^

### D Clinical pharmacology of the first G protein-biased apelin agonist, MM07

MM07 is a cyclized apelin ligand, designed to increase peptide half-life by protecting against peptidases, which also shows bias toward the G protein pathway. MM07 displayed a similar potency to [Pyr^1^]apelin-13 in G protein assays but a potency 2 orders of magnitude less than [Pyr^1^]apelin-13 in *β*-arrestin and internalization assays (eg, calculated bias factor of 1374 in vasoconstrictor vs *β*-arrestin recruitment assay).^26^ In a range of in vitro and in vivo studies MM07 is more effective than [Pyr^1^]apelin-13. MM07 produced a larger dose-dependent increase in rat cardiac inotropy after systemic infusion of the peptides^26^ and inhibited TGF*β*1/Smad signaling and TGF*β*1-induced overexpression of *α*-smooth muscle actin and collagen a1 in human fibroblasts in vitro and bleomycin-induced dermal fibrosis in mice^252^ to a greater extent than [Pyr^1^]apelin-13. Consistent with these beneficial cardiovascular and antifibrotic actions, MM07 prevented the development of raised right ventricular systolic pressure, right ventricular hypertrophy, cardiac dysfunction, and pulmonary vascular remodeling in a monocrotaline rat model of PAH.^253^ Significantly, after 4 weeks of treatment, MM07 (10 mg/kg i.p. daily) reversed established cardiopulmonary remodeling and hemodynamic changes in the Sugen/hypoxia rat model of PAH. MM07 was as at least as effective as the mixed endothelin receptor antagonist macitentan (30 mg/kg o.g. daily) that is used clinically in patients with PAH, but which failed to significantly reverse pulmonary vessel muscularization in this study.^233^ The mechanisms underpinning the superior actions of MM07 in this model have not been delineated but in addition to opposing signaling through TGF*β*, MM07 has previously been shown to be proproliferative and antiapoptotic in human pulmonary artery endothelial and smooth muscle cells^253^^,^^254^ (see section *Pulmonary hypertension*).

Additionally, MM07 was shown to be safe and biologically active in healthy human volunteers.^26^ MM07 was the first G protein-biased agonist to be tested in first in human, proof of principle studies.^26^ MM07 was shown to be biologically active, safe and well tolerated. The compound mimicked apelin in modulating peripheral arterial tone. MM07 caused a dose dependent vasodilation in the forearm of 12 healthy volunteers after infusion of MM07 (1, 10, and 100 nmol/min for 6 minutes). MM07 was more efficacious, as hypothesized, and produced a significant increase in forearm blood flow with a maximum dilatation double that seen with the endogenous peptide, [Pyr^1^]apelin-13, tested at the same concentration. Crucially MM07 reproducibly increased forearm blood flow after repeated doses (100 nmol/min), indicative of reduced receptor desensitization compared with [Pyr^1^]apelin-13, supporting the potential clinical advantage of a G protein-biased agonist. MM07 was also effective in reversing an established norepinephrine constriction and significantly increased flow in the venous bed with no adverse events observed (such as vasoconstriction).

### E Unbiased agonists: Small molecules

#### 1 E339-3D6

There has been a concerted effort to develop potent small molecule apelin agonists as tool compounds for structure-activity studies and as potential investigational drugs. E339-3D6 was the first reported nonpeptide apelin agonist with nanomolar affinity in apelin receptor binding assays. Described as peptidomimetic owing to it relatively high molecular weight (∼1750 Da), E339-3D6 is a partial agonist exhibiting variable efficacy compared with apelin in cAMP, receptor internalization and preconstructed rat aorta vasorelaxation assays.^29^ E339-3D6 was subsequently determined to be a mixture of polymethylated species with the structure of the major component (affinity for the human apelin receptor 0.69 *μ*M) used as a basis for a series of derivatives with plasma half-lives of over 10 hours and varying affinities for the apelin receptor.^255^

#### 2 ML233

The apelin receptor is structurally most closely related to the AT_1_ receptor. There have been several apelin receptor small molecule agonists identified that show some selectivity over the AT_1_ receptor. These include ML233, a small molecule identified in the Molecular Libraries Small Molecule Repository high-throughput screen of ∼330,600 compounds that had >20 fold selectivity compared with the AT_1_ receptor^30^ and a series of pyrazole-based compounds with improved affinities.^256^^,^^257^ ML233 has poor aqueous solubility and liver microsomal stability whereas modification of the novel pyrazole-based agonist scaffold has resulted in compound **13**^31^ with a Ki of 90 nM for the apelin receptor, a half-life in mice of 2.7 hours, lacked human Ether-a-go-go related gene or Ames test activity, was brain penetrant with only weak off-target reactivity. In a mouse model of diet-induced obesity compound **13** (5 and 15 mg/kg i.p., twice daily for 28 days) significantly reduced bodyweight, food consumption, fat pad weight, plasma fructosamine and fasting glucose compared with vehicle control. However, compound **13** lacks significant oral bioavailability in rats and therefore optimization was undertaken resulting in the design of the orally active, potent apelin agonist, compound **47**^32^ with a half-life in rats of 4.1 hours and oral availability of 95%. Compound **47** was less potent in *β*-arrestin recruitments assays than in G protein-dependent assays, but whether this represents G protein bias has not been determined, although a lower propensity to receptor desensitization has been hypothesized.

#### 3 BMS-986224

An important milestone is the design of compounds with improved drug-like properties; particularly, good oral bioavailability. Optimization of aryl hydroxy pyrimidinones discovered in a high through-put screen^33^ led to the discovery of BMS-986224, an orally active, full agonist with subnanomolar affinity for the apelin receptor.^34^ Preclinical evaluation of this molecule confirmed an in vitro pharmacological profile comparable to [Pyr^1^]apelin-13 in competition binding and cell based signaling assays, including G_i_/G_11/12_, *β*-arrestin-1 and -2 recruitment and receptor internalization activity. In a cardiovascular safety screen, BMS-986224 (30 *μ*M) had no appreciable activity for 18 other GPCRs. In instrumented, anesthetized rats, acute administration (short iv infusion) of either [Pyr^1^]apelin-13 (0.15–12 g/kg per minute for 10 minutes) or BMS-988224 (1–100 g/kg per minute for 15 minutes) produced largely dose-dependent increases in stroke volume, cardiac output, cardiac contractility with modest effects on mean arterial pressure and minimal impact on heart rate as previously reported for [Pyr^1^]apelin-13 and analog in healthy human studies.^26^^,^^57^^,^^66^ A randomized, double-blinded, placebo-controlled, single and multiple ascending dose study to evaluate the safety, tolerability, pharmacokinetics, and pharmacodynamics of BMS-986224 in healthy subjects and chronic HF patients with reduced ejection fraction was undertaken (https://clinicaltrials.gov/study/NCT03281122) but terminated with reason stated as a change in business objectives. A second study, a phase 1, open-label study to evaluate the pharmacokinetics, safety and tolerability of BMS-986224 in volunteers with varying degrees of renal function (https://clinicaltrials.gov/study/NCT03634969) was completed but data from these studies have not been published.

Subsequent SAR exploration of the BMS-986224 C3 oxadiazole pyridinone structure led to the identification of a chemically different series of compounds including the pyrimidinone **14a**, which retains the pharmacological profile of BMS-986224.^35^ Further optimization improving chemical and metabolic chemical stability resulted in a clinical candidate, hydroxypyridinone **14**^36^ that increased cardiac output and cardiac contractility after a short infusion in anesthetized rats. In the cynomolgus monkey 3 mg/kg oral dosing with this compound resulted in a t_1/2_ of 3.6 hours and oral bioavailability of 56%. Compound **21**, a hydroxypyrimidinone,^37^ exhibited enhanced pan-species metabolic stability in liver microsomes (>92%) and dose-dependently (1, 10, and 100 *μ*g/kg per minute) increased cardiac output and cardiac contractility with only a transient, modest reduction in mean arterial pressure and negligible effect on heart rate in the anesthetized rat. In a separate high throughput screen, a biphenyl hit showed selectivity for the apelin receptor compared with the AT_1_ receptor. SAR resulted in the design of compound **15a** that had comparable binding affinity as [Pyr^1^]apelin-13 and potency in a cAMP assay and produced the expected hemodynamic profile of an apelin agonist.^38^

#### 4 AM-8123

AM-8123 and AMG 986 [see section *Clinical pharmacology of unbiased small molecule agonist AMG 986 (Azelaprag)*] are triazole compounds developed from a high throughput screen and SAR program using cAMP, GTP*γ*S, *β*-arrestin recruitment and receptor internalization assays to confirm apelin receptor full agonist activity, comparable to [Pyr^1^]apelin-13, with selectivity over *β*_2_ and AT_1_ receptors.^39^ Molecular modeling showed that AM-8123 interacted with 3 regions of the apelin receptor orthosteric site essentially mimicking the C-terminus of apelin-13. In contrast AM-8123 inserts more deeply into a subpocket not involved in apelin-13 binding that may explain why this small molecule retains high affinity compared with the endogenous peptide. Although some mutation of the apelin receptor reduced binding affinity of AM-8123 these did not include R168A as would be predicted from the unaffected binding of CMF-019 to the naturally occurring R/H168^4.64^ variant that shows reduced binding for apelin. In vivo, AM-8123 had a half-life of 3.2 and 2.2 hours in rat and dog and oral bioavailability of 60% and 40%, respectively, in these species. Acute intravenous administration of AM-8123, in a rat model of MI with reduced ejection fraction, 6 to 8 weeks after left anterior descending coronary artery ligation, improved cardiac hemodynamics as expected for an apelin agonist. Increased contractility was also measured in anesthetized beagles with HF. These beneficial effects were maintained after chronic oral treatment with AM-8123 with a reduction in cardiac compliance detected that might be a result of the lower collagen deposition measured after treatment. Disappointingly, the combination of losartan and AM-8123 conferred no additional benefit to the individual drug suggesting use of AM-8123 and other apelin agonists might be limited to those patients not tolerating inhibition of the RAS.

### F Clinical pharmacology of small molecule agonist AMG 986 (azelaprag)

AMG 986 (azelaprag, BGE-105) was the first-in-class orally active small molecule apelin agonist to be tested in the clinic and the results reported in 5 peer reviewed studies (Table 2).^68^, ^69^, ^70^, ^71^, ^72^ Amgen licensed the compound to BioAge Labs in 2021^73^^,^^74^ and renamed azelaprag (BGE-105) for all indications. Safety was evaluated in healthy subjects, or patients with renal impairment and HF with all stating that AMG 986 was well tolerated. A further study was terminated for lack of expected efficacy rather than safety concerns.^68^

In a phase 1 study in healthy volunteers (*n* = 15) a single dose of 10 mg AMG 986 resulted in favorable pharmacokinetic parameters: area under the curve (AUC) = 9460 ng.h/mL, t_max_ = 2 hours C_max_ = 554 ng/mL, with a plasma half-life of 18.2 hours. Plasma concentration versus time displayed exponential decay, consistent with first order kinetics. Strong inhibitors of CYP3A4/P-glycoprotein, a major metabolic pathway for the compound, significantly increase AMG 986 exposure. Many HF drugs (the principal target for the compound) are metabolized by CYP3A4, and some are also influenced by permeability glycoprotein, a transport protein that can affect drug absorption and excretion.^71^ Severe renal impairment did not alter the calculated amount of drug over time in the body (AUC) or C_max_, compared with participants with normal renal function. Many older patients will have comorbidities of renal and HF.

AMG 986 was tested for safety and tolerability in 182 participants in a phase 1 study. This included healthy volunteers (*n* = 116) and a limited sample size of patients with HF (*n* = 20) in a randomized, placebo-controlled (*n* = 8), dose-escalation study with single and multiple intravenous and oral doses over 21 days.^68^ The study was negative, with the authors reporting no clinically meaningful dose dependent effects in patients with HF. AMG 986 was well tolerated at all doses (up to 650 mg/day) in both groups with the most common adverse event observed being headache (5%), the highest versus 2% placebo controls. The half-life following a single oral dose ranged from 13.2 hours (5 mg dose) to 21.0 hours (650 mg), within the optimum range for a once-a-day drug and showing a significant advance on the half-life of apelin. Absorption was rapid with estimates of t_max_ that ranged from 1.0 to 2.0 hours. However, there was no clear dose-related trend. Mean bioavailability decreased from 78% to 42% with increasing doses from 5 to 650 mg.

The pharmacokinetic data are difficult to interpret as there was a nonlinear increase in drug exposure with higher doses and minimal accumulation with repeated dosing, indicating zero order kinetics and suggesting saturation of metabolic pathways. In patients with HF, there was no statistically significant change in left ventricular ejection fraction stroke volume by Doppler. The authors cautioned that the number of participants was too small to deduce any information about efficacy. Blood pressure and cardiac output was not measured in volunteers, which would have aided understanding of target engagement.

### G Potential first-in-class apelin receptor agonist as an exerkine mimetic for muscle and metabolic aging

The scientific rationale for a beneficial role of apelin in healthy aging has been established in over 50 papers and reviews published to date, particularly the pivotal study by Vinel et al^217^ demonstrating apelin reverses age-associated sarcopenia. The results showed that apelin induced by muscle contraction was reduced with age in humans and mice and was positively associated with the beneficial effects of exercise in older persons. These results combined with other studies suggested loss of apelin signaling associated with immobilization driver of muscle atrophy. This has led to the hypothesis that restoring apelin signaling would target muscle regeneration during periods of immobility or aging. In addition, the established beneficial action on improving the cardiovascular system, homeostasis, and insulin sensitivity.

Importantly these authors also measured plasma apelin immunoreactivity in elderly people at baseline before undertaking a 1-year program of regular physical exercise and found after 6 months, those most improved on a test measuring functional decline, frailty, and disability risk had increased plasma levels. These results provided evidence a positive regulatory loop between muscle contraction, apelin and muscle physiology, which may be altered during the aging process. Contraction-induced muscle apelin acts on both muscle fiber and satellite cell differentiation, showing a beneficial effect of physical exercise with increasing age. The authors also suggested that measuring plasma apelin immunoreactivity after acute physical exercise in the elderly could represent a potential diagnostic tool (or biomarker) to predict the benefit of physical exercise. These data further support the evaluation of apelin agonists in age-associated sarcopenia.^217^

Analysis by machine learning of proprietary human aging cohort data, supported the concept that apelin levels declined with age and are strongly associated with longevity and preservation of muscle strength.^258^ These data provided the rationale for a phase 1 study that evaluated the pharmacokinetics and safety of azelaprag (as BGE-105) in healthy older adults (*n* = 16) in a single-dose, open-label, randomized crossover and multiple-dose study (Table 2, NCT06141889). A subsequent phase 1B trial was reported by the company to significantly prevent muscle atrophy in volunteers aged ≥65 during 10 days of bed rest. Muscle size, quality, and protein synthesis were all improved without major side effects.^259^

Positive results led to subsequent phase 2 trial combining azelaprag with a glucagon-like peptide-1 and glucose-dependent insulinotropic polypeptide (GLP-1/glucose-dependent insulinotropic polypeptide [GIP]) receptor agonist, tirzepatide, versus tirzepatide or azelaprag monotherapy, in older participants aged ≥55 with obesity.^73^ The rationale was that GLP-1/GIP are 2 incretin hormones that play a crucial role in regulating blood sugar but agonist targeting of these receptors result in unwanted muscle loss as well as fat. This combination of drugs was effective in increasing weight loss but preserving lean tissue in obese mice.^259^

Unfortunately, the trial was discontinued owing to increases in liver enzymes, alanine transaminase and aspartate transaminase, released into the bloodstream when the liver is stressed. This was most likely an off-target action related to the molecular structure of azelaprag rather than a direct action on the apelin receptor.^74^ As noted above azelaprag is removed by P-glycoprotein (multidrug resistant protein-1) transmembrane transporter expressed in the liver that expels drugs from cells. Preclinical studies suggest apelin peptides are beneficial in lowering of aspartate aminotransferase as well as other biomarkers.^260^ Apelin receptors remain a viable target but with modified small molecule agonists to minimize off-target actions.

### H Biased agonists: Small molecules

CMF-019 is based on a benzimidazole scaffold structure from Sanofi^261^ and was the first reported G protein-biased, small molecule apelin agonist.^41^ Predicted from computational modeling to interact with a highly conserved region of the apelin receptor, CMF-019 was shown to bind to expressed human, rat, and mouse apelin receptors with nanomolar affinity and demonstrated strong bias toward G protein signaling over *β*-arrestin (bias factor 398) and internalization pathways (bias factor 5828).^41^ A crystal structure of the apelin receptor in complex with CMF-019 was recently reported^28^ highlighting differences between small molecule and peptide binding modes; notably, R168^4.64^ is important for peptide binding but not small molecules such as CMF-019 (see section X-ray crystallography and cryo-electron microscopy). CMF-019 is a useful tool compound to explore the contribution of apelin G protein signaling in disease; for example, in PAH, as the compound protects against pulmonary artery endothelial cell apoptosis in vitro, is a vasodilator and increases cardiac output, contractility and stroke volume in normotensive rats in vivo.^41^^,^^254^ Despite having an acceptable physicochemical profile for an oral drug, the use of CMF-019 potassium salt in vivo may be limited by its relatively low solubility in saline.^254^ However, CMF-019 is relatively stable in plasma with significant compound detected 10 minutes after bolus administration in vivo compared with the very short half-life of [Pyr^1^]apelin-13.^41^ Interestingly, in the spontaneously hypertensive rat apelin mediated, endothelium-dependent relaxation was absent in isolated coronary arteries rather apelin was found to attenuate acetylcholine-induced endothelium-dependent relaxation in vitro. In contrast relaxation was obtained with CMF-019 possibly via increased Akt and eNOS phosphorylation not seen with apelin in the animal model compared with normotensive control.^262^ In cell-based models of hypertrophy and fibrosis, whilst CMF-019 exhibited some protective effects on cardiomyocytes, improving cell viability and mitochondrial function, there was no significant reduction in other prohypertrophic markers and no reversal of profibrotic signaling genes in fibroblasts treated with conditioned media from macrophages exposed to ATP and lipopolysaccharide. The authors suggested that what might be required, at least for clinical benefit in patients with HFpEF is an apelin agonist that is completely G protein-biased, lacking any effect via *β*-arrestin signaling.^43^

Using cryo-EM to interrogate CMF-019 bound to an apelin receptor-G_i_ signaling complex, mutagenesis and functional studies determined residues that to contribute to either G protein or *β*-arrestin signaling in this system.^43^ Although no single residue was critical for switching CMF-019 from a partially G protein-biased compound to 1 that lacked any *β*-arrestin activity, comparison with other small molecule apelin receptor-G_i_ complexes indicated these molecules stabilize different structures from each other and from peptide ligands, with CMF-019 inserting deeper within site 1 of the binding pocket than others (see section X-ray crystallography and cryo-electron microscopy). Using molecular dynamic simulation and a Split-and-Mix virtual screening strategy a compound AP-7 was identified that was described as fully G protein-biased as it lacked any appreciable effect in a *β*-arrestin recruitment assay at 100 *μ*M. A more conventional medicinal chemistry approach was then used to identify a second G protein-biased agonist AP-16, which also lacked activity at the *β*-arrestin pathway. These compounds had an improved profile in the in vitro hypertrophic cardiomyocytes and the fibrosis assays preserving cell viability, enhancing mitochondrial function, reducing hallmarks of both cardiac hypertrophy and fibrosis. These benefits were recapitulated in a mouse models of high fat diet induced HFpEF, with AP-7 not only effective in reducing cardiac hypertrophy and fibrosis and increasing capillary density but also in reducing obesity, improving glucose tolerance and exercise performance and reducing blood pressure.^43^

### I Clinical pharmacology of G protein biased agonist ANPA-0073

ANPA-0073 (Table 1) is the first small molecule orally active G protein-biased apelin receptor agonist to be tested in the clinic. In vitro, the compound was reported to display a modest ∼25 fold bias for cAMP inhibition versus *β*-arrestin recruitment and with reduced potency in a receptor internalization assay. Plasma half-life in the rat was 2.6 hours.^263^

Preclinically the compound was tested by oral gavage (15 mg/kg) for 21 days after monocrotaline induced PAH in the rat (reversal model) resulting in increased cardiac output and stroke volume consistent with expected apelin agonist response but with no change in heart rate. Pressure in pulmonary arteries and right ventricular hypertrophy were reduced and remodeling of pulmonary arterioles was prevented.^75^^,^^263^ ANPA-0073 also reduced lung fibrosis in a rat model. The results are in agreement with a previous study in the sugen-hypoxia reversal model using a peptide G protein biased agonist, (Williams et al^233^; section *Clinical pharmacology of the first G protein-biased apelin agonist, MM07*), particularly the reduction in vascular remodeling. These studies highlight the potential of G protein-biased apelin agonists to be disease modifying by reversing a key pathophysiological feature of PAH that are not generally modulated by current standard of care drugs.

ANPA-0073 was tested in the clinic (Table 2) to evaluate the safety, tolerability, and pharmacokinetics.^50^ Healthy volunteers (*n* = 96) were given single (2–600 mg) or multiple (75–500 mg for 7 days) ascending doses in a randomized, placebo-controlled trial. There was dose-proportional exposure from 2 to 200 mg with minimal accumulation. Food reduced peak concentration (C_max_) of the compound by ∼32% but did not affect overall drug exposure, measured as AUC. Plasma half-life was variable across the dose range, ranging from 2.4 to 9.56 hours. A longer t_1/2_ was observed in cohorts receiving 100 mg. The drug was excreted unchanged in the urine. ANPA-0073 was generally well tolerated, with no severe adverse events. Common mild side effects included headache, lethargy, nausea, and vomiting that were similar to placebo.

The pharmacokinetic profile showed dose-proportional exposure with minimal accumulation after daily Interestingly, oral ANPA-0073, at daily doses up to 500 mg for 5–6 days, did not affect echocardiographic parameters although an apelin agonist would be expected to increase cardiac output.

### J ELA agonists: Peptide analogs

Initial studies exploring binding of ELA peptides to the apelin receptor employed an alkaline phosphatase tagged ELA-32 analog (AP-apela) in CHO cells stably expressing the apelin receptor in which ELA-32 and ELA-21 inhibited AP-apela binding with comparable affinity. C-terminal alanine substitutions of ELA-21 resulted in progressively reduced binding affinities (Table 1).^24^ ELA peptides have a short half-life when incubated with rat plasma of <2 minutes for ELA-32 and structure activity relationship studies show that ELA(19-32) is the shortest ELA analog that retains comparable affinity and potency to the predicted full length endogenous peptide ELA-32.^52^ ELA(23-32) (or ELA-14) is the shortest ELA peptide that binds the apelin receptor with nanomolar affinity (K_i_ 16M) and this could be improved by substituting the Met23 by norleucine. Further exploration of the C-terminal sequence of ELA-32 resulted in a series of synthetic ELA peptides exhibiting varying degrees of selectivity in G_i1_, G_12_, and *β*-arrestin assays.^114^ PEG and palmitic acid (pal) modifications produced analogs including Pal-E11 (K_i_ = 0.12 nM; t_1/2_ = 22.58 minutes) and PEG6-E11 (K_i_ = 0.16 nM), with improved receptor affinity and in vivo plasma stability compared with ELA-11 (K_i_ = 2.05 nM; t_1/2_ = 0.35 minutes) and renoprotective actions in a mouse model of renal ischemia/reperfusion injury.^24^

## VIII Antagonists

### A Peptides

#### 1 Apelin-13 F13A

For discussion of the pharmacology of apelin-13 F13A (see section *Apelin-13 analogs*).

#### 2 ALX40-4C and protamine: Polycationic peptide antagonists

The apelin receptor is a coreceptor for HIV, at least in vitro. ALX40-4C, which contains 9 arginine residues, was originally designed as a structural mimetic of the HIV Tat protein to disrupt the interaction of HIV to its RNA target trans-activation response element. ALX40-4C was subsequently shown to selectively inhibit the X4-tropic HIV coreceptor CXCR4, with no effect at the CCR5 receptor.^44^ Despite the limited sequence similarity between these 2 class A GPCRs, CXCR4 has been used as a template in homology modeling of the apelin receptor.^46^ CXCR4, such as the apelin receptor, has a positively charged endogenous agonist CXCL12 and both receptors have negatively charged surfaces on their extracellular loop that may allow interaction with ALX40-4C with both receptors. This compound competitively inhibited apelin binding in the low micromolar range and prevented apelin-induced receptor internalization.^44^ Protamines are small, intrinsically disordered proteins, typically comprising 30–50 amino that are also rich in arginine residues and so strongly positively charged at physiological pH. Clinically, protamine is used to reverse the effect of heparin overdose or after surgery. Similar to ALX40-4C, protamine is a polycationic peptide that binds to the apelin receptor with an affinity of ∼400 nM and is an antagonist of apelin and ELA-mediated *β*-arrestin recruitment and receptor internalization. Protamine lacked affinity at other GPCRs except for CXCR4, inhibiting CXCL12 activity by 88% at 3 *μ*M. Protamine blocked apelin-induced angiogenesis in a cell-based assay and reduced tumor growth in a mouse model, prevented the hypotensive action of apelin measured by telemetry and the reversed the beneficial effect of apelin on glucose tolerance.^45^

### B Clinical pharmacology of MM54

MM54, a cyclic peptide, was designed using a bivalent ligand approach where 2 segments containing the RPRL motif of apelin were joined by a dipeptide linker.^46^ MM54 was the first competitive apelin receptor antagonist reported with nanomolar affinity for cloned human apelin receptor with no affinity for the AT_1_ receptor and was initially shown to antagonize apelin responses in cAMP and *β*-arrestin assays.

After intraperitoneal administration into healthy mice MM54 could be detected in plasma and brain. In glioblastoma multiforme the apelin system is implicated as an important regulator of tumor expansion and maintenance. In mice with intracranial xenografts from human glioblastoma patients, MM54 reduced vascularization and growth of tumors and increased survival compared with nontreated animals. The drug did not exhibit overt toxicity or detrimental effects on metabolic and cardiovascular parameters. Moreover, at least in vitro, MM54 and the current best therapy temozolomide were synergistic. It was shown that MM54 likely inactivates apelin signaling in glioblastoma stem cells by phosphorylation of GSK3*β*.^223^

In a subsequent study, MM54 was reported to bind to human heart with an affinity of ∼300 nM, but MM54 induced a biased signaling profile at the apelin receptor by activating the beneficial G_i_ protein pathway (pD_2_ = 5.9) while acting as an antagonist, blocking harmful *β*-arrestin signaling (pK_B_ = 6.93) and apelin-mediated receptor internalization (pK_B_ = 5.89). Importantly, this dual action translated, when infused into the forearm of human volunteers, into a significant 76% increase in blood flow at a dose of 100 nmol/min and a 54% reversal of noradrenaline-induced venoconstriction in the human hand vein.^76^ Both observations indicated G protein mediated vasodilatation via the apelin receptor. MM54 is therefore a biased compound that likely stabilizes the apelin receptor in a conformation distinct from other G protein-biased agonists that still retain some agonist activity through the G-arrestin pathway.

As part of the same program, linear peptide apelin receptor antagonists have been developed including MM193 and MM315 (WO2019193355A1)^47^ that show efficacy in in vitro and in vivo glioblastoma cancer models,^223^^,^^264^ with MM315 significantly prolonging the survival of athymic mice implanted orthotopically with a human-derived glioblastoma cell-line. Compared with saline control, mice receiving MM315 1.5 mg/kg 3 times per week intraperitoneally survived for 30 weeks longer. The mechanism of action of apelin antagonist in glioblastoma is not fully understood and, in this study, did not appear to involve inhibition of neovascularization.

### C Small molecules

#### 1 ML221

ML221 is a small-molecule antagonist discovered from the same high-throughput screening program that discovered ML233. ML221 inhibited G protein and *β*-arrestin responses with submicromolar or micromolar potency; however, its utility as a tool compound is limited by its poor solubility and rapid metabolism because of its poor hepatic microsomal stability.^48^ Despite this, ML221 has been used to implicate apelin and ELA signaling in a range of animal models including cancer, neuropathic pain, and ocular and reproductive disorders.

#### 2 Aminoquinolines

Aminoquinoline compounds were identified in a high throughput screen as apelin antagonists using an apelin-induced reduction in forskolin stimulated cAMP assay.^49^ One of these shared a 4-chloroaminoquinoline moiety with 2 known antimalarials, amodiaquine and glafenine (use limited by serious side effects). Together with 3 structurally related commercially available compounds, these 4-chloroaminoquinolines were more fully characterized as noncompetitive apelin receptor agonists of apelin responses in the cAMP assay but did not block apelin in a *β*-arrestin assay (indicative of G protein bias) or compete for binding of iodinated apelin in membranes expressing the human apelin receptor. Amodiaquine was found to inhibit apelin stimulated tube formation of human retinal microvascular endothelial cells, shown to express the apelin receptor, to the same extent or better than ML221, and reduced pathological neovascularization in mice in vivo confirming the potential of apelin antagonists as antiangiogenic in conditions such as diabetic retinopathy.^49^

## IX Apelin and ELA physiology and pathophysiology

The expression of the apelin receptor and endogenous ligands in human tissues and cells described in sections *Apelin receptor distribution* and *endogenous peptide distribution* above is indicative of the emerging physiological role of the apelin receptor in heart, kidney, liver, gastrointestinal tract, skeletal muscle, reproductive tissues, blood vessels, brain and as an adipokine and regulator of fluid homeostasis. It is therefore unsurprising that dysregulation of the apelin system may contribute to a wide range of pathophysiological conditions^77^^,^^78^ stimulating interest in the therapeutic potential of drugs targeting the apelin system.^79^^,^^81^^,^^82^ This section focuses on studies in PAH^23^^,^^24^^,^^62^^,^^142^^,^^218^^,^^253^ and HF,^7^^,^^34^^,^^56^^,^^57^^,^^59^^,^^68^^,^^218^^,^^235^^,^^241^ diabetes and metabolic disorders,^63^, ^64^, ^65^^,^^73^^,^^74^^,^^77^^,^^78^^,^^99^^,^^100^^,^^219^^,^^234^ renal disease,^24^^,^^66^^,^^129^ and disorders of fluid homeostasis.^13^^,^^80^ The effects of the endogenous ligands has been investigated in clinical studies in healthy volunteers and relevant patient groups with synthetic agonists used to show proof-of-concept benefit in animal models.^79^

### A Pulmonary hypertension

Apelin, ELA, and their shared APJ receptor have emerged as pivotal regulators in PAH, consistent with their vasodilatory properties and positive inotropic cardiac effects,^265^^,^^266^ with growing evidence supporting their antiremodeling capabilities. PAH represents group 1 PH in the World Health Organization classification system, characterized by distinct precapillary pulmonary vascular pathology. Extensive studies demonstrate consistent apelin downregulation in patients with group 1 PAH, including idiopathic PAH and PAH associated with drugs/toxins or autoimmune diseases,^142^^,^^143^ particularly evident in pulmonary arterial endothelial cells from patients with PAH versus controls.^267^^,^^268^ Recent work extends these observations to chronic obstructive pulmonary disease-associated PH^269^ and cirrhosis,^270^ while showing preserved levels in systemic sclerosis-PAH^271^ and elevated levels in other PH forms.^141^^,^^272^ These discrepancies likely reflect PH class/subtype heterogeneity, individual variations, and methodological differences in assays measuring distinct apelin fragments. Large multicenter studies are needed to evaluate circulating apelin/ELA or specific peptide isoforms as diagnostic or prognostic biomarkers in PH. Single-cell RNA sequencing reveals expanded apelin-expressing proangiogenic endothelial subsets in systemic sclerosis-PAH lungs,^273^ possibly suggesting localized compensation. Although apelin/APJ transcripts mark specialized capillary endothelial cell subsets in single-cell studies,^274^ their protein expression can be found in larger (though still small) pulmonary vessels,^60^^,^^150^^,^^214^^,^^225^ highlighting important considerations for interpreting such data.

Apelin deficiency has been consistently observed in the right ventricle of both monocrotaline-exposed rats^275^ and Sugen5416/hypoxia rat models of PAH,^276^^,^^277^ with apelin levels demonstrating significant correlation with right ventricular contractile and diastolic function.^278^ Similar to apelin, ELA shows marked downregulation in cardiopulmonary tissues from both patients with PAH and experimental models.^13^ Genetic ablation of apelin exacerbates hypoxic PH, resulting in elevated right ventricular systolic pressure, enhanced muscularization of alveolar wall arteries, and pronounced pulmonary microvascular rarefaction compared with wild-type controls.^142^ At the cellular level, apelin promotes survival and inhibits apoptosis in pulmonary arterial endothelial cells while suppressing proliferation and inducing apoptosis in pulmonary arterial smooth muscle cells.^253^^,^^267^^,^^268^ The apelin system is regulated through multiple interconnected pathways in PAH. Bone morphogenetic protein receptor type 2 signaling, frequently affected by mutations in heritable PAH, serves as a key upstream regulator of pulmonary endothelial apelin expression.^279^ Notably, a protective estrogen receptor *α*/bone morphogenetic protein receptor type 2/apelin axis has been identified in right ventricular remodeling, with estrogen-mediated restoration of this axis potentially explaining sex differences in PAH.^280^ Additional regulation occurs through PAH-relevant transcription factors including peroxisome proliferator-activated receptor *γ* and p53,^269^^,^^281^ as well as microRNA networks (miR-130/301 and miR-335-3p) that modulate apelin and its receptor.^282^^,^^283^ These complex regulatory mechanisms position the apelin system as a critical integrator of hormonal, inflammatory, and metabolic signals in the stressed cardiopulmonary system. Cumulatively, these findings strongly support the inhibitory role of apelin signaling in pulmonary vascular remodeling, the central pathological process driving PAH progression.

Notably, despite significant downregulation of endogenous apelin in PAH, the APJ receptor remains functionally expressed (though possibly reduced) in diseased tissues^268^^,^^284^ presenting a valuable therapeutic opportunity for exogenous ligand replacement. This strategy is supported by the observation that circulating apelin and ELA concentrations in healthy individuals, although relatively low,^13^ fall within the effective range of EC_50_ values measured in human tissue assays, suggesting that therapeutic supplementation could restore physiological signaling.^53^ Multiple therapeutic approaches targeting this pathway have demonstrated efficacy: peptide replacement therapy through daily administration of either apelin^268^^,^^275^ or ELA^13^ has shown benefits in rodent models; gene therapy using adeno-associated virus-delivered ELA-32 significantly improves hemodynamics and vascular remodeling in monocrotaline-induced PAH^285^; apelin-overexpressing MSCs exhibit sustained pulmonary engraftment with therapeutic effects^286^; and modulators of apelin expression or signaling, including tacrolimus, elafin, and nutlin-3, have shown promise in preclinical studies or small trials.^281^^,^^287^^,^^288^ These diverse but complementary approaches collectively validate the apelin-APJ system as a robust therapeutic target in PAH. Pharmacological activation of the apelin pathway has shown significant therapeutic potential, with the peptide agonist MM07 demonstrating efficacy comparable to macitentan in reducing right ventricular hypertrophy, systolic pressure elevation, and pulmonary arteriole muscularization in rat models.^233^^,^^253^ It remains to be determined whether beneficial effects can be achieved by small molecule apelin receptor agonists, which also have antiapoptotic effects on pulmonary arterial endothelial cells.^254^ Clinical validation comes from a double-blind randomized crossover trial where [Pyr^1^]apelin-13 infusion significantly improved hemodynamics in patients with PAH, particularly when combined with phosphodiesterase type 5 inhibitors.^62^ Similarly, earlier in vitro work demonstrated apelin’s ability to counteract endothelin-1 vasoconstriction.^5^ These findings collectively suggest that apelin-based therapies may complement existing PAH treatment regimens.

The compelling preclinical and clinical evidence positions the apelin-apelin receptor system as a multifaceted therapeutic target in PH. The preserved receptor expression despite ligand downregulation creates a unique opportunity for replacement strategies, and the demonstrated efficacy of diverse approaches, from peptide administration to gene therapy and small molecule agonists, underscores the pathway’s versatility. Although clinical evidence remains preliminary, the observed synergy with current therapies^62^ and potential for pleiotropic benefits (including antifibrotic and cardioprotective effects) warrant further investigation. Future studies should focus on optimizing long-term safety and efficacy of next generation apelin receptor agonists, and expanding therapeutic applications to PH associated with left heart or lung diseases.

### B Heart failure

In HF, multiple studies have demonstrated decreased plasma apelin levels in human patients^137^^,^^289^ and parallel reductions in cardiac tissue apelin content across various animal models.^290^^,^^291^ Apelin knockout mice developed impaired contractility under pressure overload,^174^ whereas apelin receptor knockout exacerbated cardiac dysfunction in doxorubicin-induced cardiotoxicity.^292^ Importantly, mechanical unloading with left ventricular assist devices restores apelin signaling,^138^^,^^139^ suggesting a dynamic response to hemodynamic stress. The apelin receptor also exhibits disease-stage-dependent regulation, with most studies reporting downregulation in chronic HF,^290^, ^291^, ^292^ though exceptions suggest compensatory mechanisms in early disease or model-specific effects.^292^

On of the most common causes of HF is coronary artery disease leading to myocardial ischemia. Importantly, Oudit’s group^293^ have shown that patients with ischemic HF have reduced levels of apelin. In a mouse model, loss of apelin worsened outcomes, including higher mortality, larger infarcts, increased inflammation, impaired prosurvival signaling, and more severe HF. Apelin deficiency also reduced vascular sprouting, impaired endothelial progenitor cell function, and weakened myocardial angiogenesis. An apelin analog resistant to proteolytic cleavage of the C-terminal phenylalanine by ACE2 (NleAibBrF pyr-1-apelin-13) mimicked the protective effect of apelin in reducing myocardial ischemia-reperfusion injury by enhancing survival pathways and promoting angiogenesis. The study provided evidence for the potential of synthetic agonists targeting this pathway.

Recent studies refined our understanding of apelin system regulation in HF. Although early work reported elevated apelin in early-stage HF followed by decline,^138^ systematic analysis across the New York Heart Association functional classes revealed peak apelin levels in class II patients before subsequent decline,^294^ indicating a transient compensatory response. The related peptide ELA shows an inverse correlation with HF severity, with progressively lower levels associated with advancing New York Heart Association class and worsening left ventricular (LV) function.^145^ Clinically, apelin enhances risk prediction when combined with existing biomarkers, improving 1-year survival forecasts in advanced HF,^295^ whereas the apelin/N terminal pro B-type natriuretic peptide ratio more effectively identifies HFpEF in patients with diabetes.^296^ These findings align with the “obesity paradox,” where higher apelin levels in patients with overweight correlate with better outcomes.^297^

Therapeutic administration of apelin demonstrates consistent benefits across diverse HF and related models, spanning small and large animals. In rat models of isoproterenol-induced cardiomyopathy,^298^ salt-sensitive hypertension,^291^ and coronary artery ligation^299^ apelin administration improves cardiac function and attenuates adverse remodeling. Recent pharmacological advancements have significantly improved the therapeutic potential of apelin pathway modulation.

Abdominal aortic aneurysm (AAA) is a common vascular disease where the vessel wall weakens and can rupture, causing internal bleeding. There is no approved drug treatment. A study from Oudit’s group^300^ uncovered a protective role for apelin in preventing AAA formation. *Apln* knockout mice that were infused with Ang II for 4 weeks, developed more severe aneurysms, experienced more aortic ruptures, and had lower survival. The worsening disease was driven by increased smooth muscle cell apoptosis and oxidative stress, both in *Apln*-deficient mouse aortas and in cultured human and mouse smooth muscle cells. In human AAA tissue, neutral endopeptidase that also metabolizes and inactivates apelin-17 peptide. was found to be upregulated and was the rationale for the design of APLN-NMeLeu9-A2, an analog resistant to neutral endopeptidase cleavage. This reduced Ang II-mediated adverse aortic remodeling and AAA formation suggest these compounds could be a therapy for vascular disease.

Structural modifications have yielded apelin analogs with superior therapeutic profiles. The chemically modified [MeArg^1^,NLe^10^]-apelin12 produces more pronounced improvements in left ventricular fractional shortening and ejection fraction compared with native apelin-12 in doxorubicin-induced cardiomyopathy.^301^ Similarly, LIT01-196, a metabolically stabilized apelin-17 analog, preserves cardiac function and reduces fibrotic biomarkers after MI without inducing hypotension.^241^ Liposomal encapsulation of [Pyr^1^]apelin-13 prolongs its hemodynamic effects and reduces fibrosis in pressure-overloaded mice,^302^ whereas fatty acid conjugation enhances peptide stability in MI models.^303^ Tang et al,^304^ developed an innovative apelin-13-releasing patch that maintains therapeutic delivery for 28 days, significantly reducing scar formation in chronic MI. Mechanistic studies have elucidated multiple cardioprotective pathways of apelin signaling. Apelin exerts potent antifibrotic effects by inhibiting TGF-*β*/mothers against decapentaplegic homolog 2 signaling^305^ and preventing mothers against decapentaplegic homolog 2/3 activation in cardiac fibroblasts.^304^ It also regulates metabolism through Sirt3-dependent mechanisms, enhancing glucose utilization^306^ and preserving mitochondrial function.^307^ The system’s regulation extends to epigenetic control, with miR-204 modulating apelin receptor endocytosis during mechanical stress^308^ and long noncoding RNA neuron navigator 2-antisense RNA 2 stabilizing apelin mRNA by sequestering miR-31.^309^ These multifaceted actions position the apelin system as a promising therapeutic target for diverse HF phenotypes.

ELA has emerged as an equally promising therapeutic target. Sustained ELA delivery via osmotic pump improves both cardiac function (LV ejection fraction) and renal parameters (blood urea nitrogen and serum creatinine) post-MI.^310^ Sainsily et al^311^ conducted a direct comparison between ELA-32 and [Pyr^1^]apelin-13 in spontaneously hypertensive rats fed a high-salt diet, demonstrating ELA’s superior efficacy in reducing blood pressure, cardiovascular-renal dysfunction, fibrosis, and hypertrophy. These beneficial effects were mediated through modulation of the ACE/ACE2/neprilysin axis in both cardiac and renal tissues. Pharmacokinetic limitations of the ELA peptide can be addressed by creating an IgG Fc-ELA-21 fusion protein, which significantly increased angiogenesis and reduced apoptosis near infarct areas when administered daily for 4 weeks in MI rats, effects not observed with native ELA-21 peptide.^312^ At the cellular level, ELA-11 protects against doxorubicin-induced cardiac injury by regulating oxidative stress-mediated apoptosis through activation of both PI3K/AKT and extracellular signal-regulated kinase/MAPK signaling pathways.^313^ Additionally, ELA has been shown to inhibit ANG II-induced ferroptosis in cardiac microvascular endothelial cells via the interleukin 6/activator of transcription 3/glutathione peroxidase 4 signaling axis.^314^

The apelin system demonstrates clinically significant interactions with existing HF therapies, as revealed by recent studies. SGLT2 inhibitors exhibit notable interplay with this pathway: canagliflozin improves diastolic function in salt-sensitive HFpEF through apelin upregulation,^315^ whereas dapagliflozin treatment increases apelin levels in patients with type 2 diabetes-HF.^316^ Proteomic analysis from the TOPCAT trial identified apelin liver signaling as a top pathway associated with spironolactone response in patients with HFpEF, though its functional significance requires further investigation.^317^ The system may also modulate responses to angiotensin receptor-neprilysin inhibition, as the apelin signaling pathway shows significant enrichment in sacubitril/valsartan low-responders.^318^ Furthermore, mechanistic studies reveal that montelukast, a cysteinyl leukotriene receptor antagonist, depends on intact apelin receptor signaling for its antifibrotic effects in cardiac fibroblasts, with receptor silencing abolishing these benefits.^319^

Despite these advances, important questions remain regarding the apelin system’s complex biology in advanced HF. Paradoxically, upregulation of apelin pathway genes has been observed in end-stage dilated cardiomyopathy,^320^ whereas miR-185-5p elevated in patients with cardiomyopathy, targets the apelin receptor to inhibit apelin’s antifibrotic effects.^321^ The clinical translation of apelin therapeutics has recently achieved key milestones. Winkle et al,^68^ conducted the first-in-human study of apelin receptor agonist AMG 986 in patients with HF, demonstrating dose-dependent improvements in stroke volume and LV ejection fraction without significant safety concerns. AM-8123, a C-terminal apelin-13 mimetic, improves systolic function and reduces vascular resistance in rodent and canine HF models, with chronic oral administration achieving comparable antifibrotic effects to losartan.^39^ The advent of biased agonists such as WN561,^42^ which selectively activates G-protein over *β*-arrestin signaling, may further optimize therapeutic efficacy while minimizing the adverse effects of cardiac hypertrophy. These developments underscore the apelin system’s growing potential as both a therapeutic target and biomarker, particularly for HF phenotypes characterized by fibrosis, metabolic dysfunction, or maladaptive remodeling.

### C Diabetes and metabolic disorders

The apelin-apelin receptor system has emerged as a crucial regulator of metabolic homeostasis, with accumulating evidence from both foundational and recent studies illuminating its complex pathophysiology and therapeutic potential in diabetes mellitus and related metabolic disorders. Apelin has been highlighted for its role in inhibiting insulin secretion while decreasing glucose levels and increasing insulin sensitivity.^53^ In pancreatic islets, apelin suppresses both glucose- and GLP-1-stimulated insulin secretion,^322^^,^^323^ with genetic ablation of the apelin receptor in *β*-cells leading to reduced islet mass and impaired insulin output.^324^^,^^325^ These effects complement apelin’s insulin-sensitizing actions through skeletal muscle glucose uptake^234^ and mitochondrial biogenesis.^326^ Recent studies reveal additional mechanisms, including apelin-13-mediated improvement of insulin resistance and adipose tissue inflammation via nitric oxide-dependent and ACE2/angiotensin-(1–7) pathways.^260^

Recent epidemiological studies have strengthened the clinical relevance of apelin, with circulating levels predicting the risk of developing type 2 diabetes.^327^ Notably, apelin-12 outperforms traditional adipokines such as leptin and adiponectin in predicting metabolic syndrome in children with obese.^328^ In gestational diabetes, apelin-13 protects pancreatic function via PI3K/AKT pathways,^329^ while inversely correlated with ELA levels.^330^ Therapeutically, SGLT2 inhibitors such as dapagliflozin modulate apelin levels in patients with diabetic HF.^316^ Additional modulators include nutritional and dietary factors, with dietary glycemic index affecting adipose apelin expression^331^ and L-carnitine reducing adipose apelin receptor expression.^332^ Epigenetic mechanisms, such as long noncoding RNA nuclear enriched abundant transcript 1, destabilize apelin mRNA under diabetic conditions.^333^ Even exercise capacity in apelin knockout mice proves modifiable through intermittent hypoxia,^334^ demonstrating responsiveness of the apelin system to diverse metabolic and environmental stimuli.

The apelin system’s involvement in diabetic complications reveals both therapeutic promise and mechanistic paradoxes. In diabetic nephropathy, apelin-13 exhibits multifaceted renoprotective effects through upregulation of renal apelin receptor and eNOS with concurrent *α*-smooth muscle actin suppression,^335^ attenuation of glomerular basement membrane thickening via sirtuin 3-Kruppel-like factor 15,^336^ improvement of renal perfusion through PI3K/AKT/glycogen synthase kinase-3*β*/nuclear factor erythroid 2-related factor 2 signaling,^337^ and antifibrotic effects via Runt-related transcription factor 3/sirtuin 1/Forkhead box class O pathways.^338^ However, this protective role contrasts with observations where elevated serum apelin-13 correlates with TGF-*β*1 levels and proteinuria,^339^ and evidence suggesting that persistent high levels of apelin may worsen podocyte dysfunction.^340^ In contrast, ELA demonstrates more consistent renal benefits, preserving kidney function through activating autophagy pathways^341^ and suppressing AMPK/NOD-like receptor protein 3-mediated signaling.^342^

The role of apelin in diabetic retinopathy exemplifies its paradoxical nature, exhibiting both pathogenic and protective effects. Although apelin promotes pathological neovascularization through vascular endothelial growth factor coexpression,^343^ it simultaneously maintains vascular integrity by preserving pericyte function and tight junctions.^344^ Clinically, serum apelin levels correlate with retinopathy severity,^345^ suggesting its potential as a diagnostic biomarker. At the cellular level, apelin contributes to disease progression by activating Janus kinase 2/activator of transcription 3 signaling in Müller cells, leading to retinal fibrosis.^346^

Apelin demonstrates significant therapeutic potential for cardiovascular complications in diabetes, targeting both vascular and myocardial pathologies. In peripheral vascular disease, apelin-13 enhances limb reperfusion through Akt/AMPK/eNOS and Ras homolog gene family member A/Rho-associated coiled-coil kinase signaling pathways,^347^ while simultaneously inhibiting high glucose-induced aortic smooth muscle calcification via reactive oxygen species suppression and MAPK/PI3K/AKT modulation.^348^ For diabetic cardiomyopathy, apelin exerts cardioprotection through suppression of peroxisome proliferator-activated receptor *α*-mediated lipotoxicity,^349^ reduction of ischemia-reperfusion injury via PI3K/p38MAPK,^350^ and improvement of endothelial function via nuclear factor *κ*B pathways.^351^ The regulatory network extends to microRNA-mediated modulation of apelin signaling.^352^ In addition to apelin, ELA protects against diabetic cardiac fibrosis through sirtuin 3-mediated Forkhead box class O 3a deacetylation and antioxidant effects.^353^

Emerging research has elucidated apelin’s role in nonclassical diabetic complications, revealing novel therapeutic opportunities. In cognitive impairment, apelin-13 mitigates diabetes-induced decline by enhancing hippocampal mitochondrial function via sirtuin 3/Forkhead box class O 3a activation,^354^ with clinical correlations showing serum apelin levels predict cognitive dysfunction in patients with diabetes.^355^ Reproductive system manifestations demonstrate that pathologically elevated apelin levels impair testosterone production and spermatogenesis via blood-testis barrier disruption, effects reversible through apelin receptor blockade.^356^^,^^357^ Gestational diabetes models reveal transgenerational benefits as maternal exercise and insulin therapy reduce apelin-13 while improving offspring testicular apelin receptor expression and spermatogenesis.^358^ Therapeutically, ELA accelerates diabetic wound healing by suppressing TNF receptor-associated factor 1/nuclear factor *κ*B-mediated oxidative damage,^359^ suggesting potential synergy with existing ulcer treatments through endothelial protection.

Recent advances in apelin-based therapeutics have focused on 2 key strategies: stable peptide analogs and innovative delivery systems. [pGlu][Lys8GluPAL]apelin-13 amide demonstrates *β*-cell protective effects by preserving *β*-cell identity, inhibiting *β*-to-*α*-cell transdifferentiation, and stimulating *β*-cell proliferation in diabetic models.^360^^,^^361^ Apelin-36-[L28A] and apelin-36-[L28C(30 kDa-PEG)], which are modified apelin-36 peptides, prevented diet-induced obesity by improving glucose tolerance and reducing blood glucose, cholesterol, and low-density lipoprotein without affecting blood pressure.^243^ Initially considered to be apelin receptor-independent, they were later identified as G protein-biased apelin receptor ligands.^244^ Small extracellular vesicles derived from apelin-overexpressing MSCs enhance Akt and AMPK pathway activity in adipocytes, improving glycemic control and pancreatic *β*-cell function in diabetic models.^362^ Similarly, conditioned medium from Wharton jelly MSCs modulates apelin expression in diabetic liver and kidney tissues, reducing TGF-*β* while enhancing apelin production.^204^ These innovations may collectively address both the pharmacokinetic challenges and therapeutic potential of apelin modulation, while their efficacy in clinical studies and the potential utility of small molecule apelin receptor agonists remain to be tested.

Despite these advances, important knowledge gaps persist regarding tissue-specific apelin receptor signaling and the long-term safety of therapeutic modulation. The apelin system’s pleiotropic effects, exemplified by its capacity to both ameliorate and exacerbate diabetic complications depending on context, demand sophisticated targeting strategies. Future research directions may explore tissue-specific apelin receptor modulation, combination therapies with conventional antidiabetic agents, and personalized approaches based on apelin biomarker profiling.

### D Renal and fluid homeostasis

#### 1 Physiology

##### a Renal hemodynamics

**Fig. 11:**
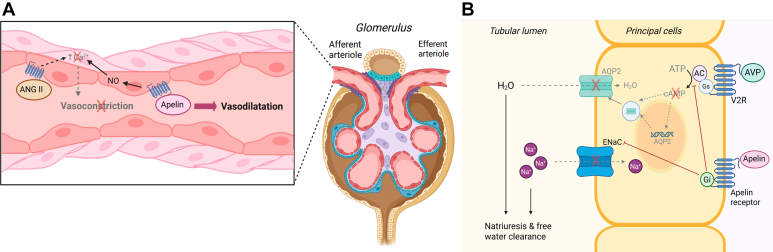
(A) Apelin regulates glomerular hemodynamics. In the afferent and efferent arteriole, apelin opposes ANG II-mediated vasoconstriction via the production of nitric oxide (NO) and subsequent inhibition in the increase in intracellular calcium. Figure created in BioRender. https://BioRender.com/avjs170. (B) The renal actions of apelin influence salt and water balance. In the kidney, apelin directly inhibits the AVP-mediated insertion of aquaporin 2 (AQP2) channels in the apical membrane of the principal cells of the collecting duct, promoting aquaresis. Apelin also inhibits sodium resorption via inhibition of the epithelial sodium channel, which is activated by aldosterone and AVP. Figure created in BioRender. https://BioRender.com/ixdcds4

Renal blood flow is tightly regulated to maintain glomerular filtration rate (GFR) irrespective of changes in systemic hemodynamics, a process known as autoregulation. ANG II is key in autoregulation, increasing intracellular calcium to cause vasoconstriction of afferent and efferent glomerular arterioles, with a preferential effect on the efferent arteriole. Apelin contributes to the regulation of glomerular hemodynamics (Fig. 11). In rats, apelin reverses ANG II-induced vasoconstriction of glomerular arterioles by rapidly reducing intracellular calcium levels, effects mediated by endothelial nitric oxide production.^363^ Recent studies in healthy humans support these findings, with a 30-minute infusion of [Pyr^1^]apelin-13 increasing renal blood flow by ∼15%. This effect was independent of changes in systemic hemodynamics suggesting a direct action of the peptide on the kidney. Given the lack of effect on iohexol-measured GFR, a preferential vasodilatory effect of apelin on the efferent arteriole is likely.^66^ At present, there are no studies examining the effects of ELA on renal hemodynamics.

##### b Salt and water balance

The apelin system acts in opposition to arginine-vasopressin (AVP) to maintain salt and water balance (Fig. 11). There is crosstalk between apelin and AVP in the brain and kidney. In the brain, there is colocalization of apelin, the apelin receptor and AVP within magnocellular neurons with reciprocal regulation of the peptides.^123^^,^^246^^,^^364^ In the kidney, AVP activates V2 receptors on principal cells leading to aquaporin 2 channel insertion in the cell apical membrane and subsequent water resorption. In vitro studies show that apelin inhibits this, promoting aquaresis.^365^^,^^366^ High concentrations of AVP also increase activity of the epithelial sodium channel in the collecting duct to increase sodium reabsorption. In mouse cortical collecting duct cells, apelin reduces epithelial sodium channel-associated transepithelial sodium current.^367^

In preclinical studies, central (intracerebroventricular) and intravenous administration of apelin and ELA promote diuresis with no significant effect on sodium and potassium excretion.^24^^,^^52^^,^^123^^,^^246^^,^^363^^,^^366^ Interestingly, in rats ELA-32 had a 5-fold more potent effect than apelin.^24^ Animal studies have also established that the apelin receptor agonist LIT01-196 promotes aquaresis via central and peripheral actions.^19^^,^^240^ In humans, apelin induces diuresis. Intravenous infusions of both 1 and 30 nmol/min [Pyr^1^]apelin-13 increased free water clearance by ∼10%, with an associated ∼30% rise in natriuresis. These effects were independent of changes in systemic hemodynamics (which influence salt and water excretion), suggesting a direct tubular effect.^66^ Additionally, modulating plasma osmolality in healthy humans resulted in parallel reciprocal changes in circulating apelin and AVP, supporting a role for apelin in salt and water balance.^368^

#### 2 Acute kidney injury

Kidney injury alters expression of the apelin system,^369^, ^370^, ^371^, ^372^ and most studies suggest that treatment with apelin or ELA is protective.^310^^,^^369^^,^^370^^,^^373^, ^374^, ^375^ For example, in ischemia-reperfusion injury (IRI), treatment with apelin-13 prevents downregulation of apelin mRNA and upregulation of hypoxia-inducible factor 1*α* protein and transforming growth factor *β* (TGF*β*); inflammation is also reduced.^369^ Apelin also reduces podocyte and tubular injury, and preserves kidney function.^369^^,^^375^ Interestingly, apelin also offered protection when given before injury, suggesting potential therapeutic application where acute kidney injury might be likely (eg, before aortic aneurysm repair).^374^ However, recent data are conflicting, with apelin-13 treatment appearing to promote tubular injury in mice after IRI.^372^ It is noteworthy that the dose of apelin administered in this study was significantly higher, raising the possibility of a dose-dependent biphasic effect.

ELA also protects. In a mouse model of IRI, ELA-11 and ELA-32 have anti-inflammatory and antifibrotic effects and preserves kidney function, with greater benefit seen with ELA-11.^370^ Notably, these effects did not appear to be mediated via the apelin receptor and synergistic protection was suggested in vitro, where combination treatment with ELA-11 or ELA-32 and apelin-13 improved cell viability.^370^ An ELA–IgG-based chimeric fusion protein, Fc-ELA-21, has been developed to overcome the short half-life of ELA peptides. As with apelin, pretreatment with Fc-ELA-21 protects against IRI and these effects appeared to be mediated via the Akt signaling pathway.^376^

#### 3 Chronic kidney disease

##### a Nondiabetic chronic kidney disease

CKD describes permanent and often progressive kidney injury. CKD may be due to a range of causes, and it is predicted to be the fifth leading cause of life-years lost by 2040.^377^ CKD is independently associated with cardiovascular disease, which is also its commonest complication. Current evidence-based guidelines recommend treatment with a RAS inhibitor and a sodium-glucose cotransporter 2 (SGLT2) inhibitor to reduce proteinuria and slow GFR decline, with other agents emerging.^378^ However, despite current therapies, patients with CKD have significant residual renal and cardiovascular risk.^379^ Thus, the apelin system is an appealing therapeutic target.^380^ In humans, plasma apelin concentrations associate with kidney damage, increasing alongside declining GFR and increasing albuminuria.^129^ Interestingly, circulating apelin concentration associates independently with 5-year GFR decline suggesting its potential as a clinical biomarker. Recent studies suggest that apelin offers cardiorenal protection in patients with nondiabetic CKD.^66^ In a randomized, double-blind, placebo-controlled crossover study, a 30-minute infusion of both a low and a high dose of [Pyr^1^]apelin-13 increased renal blood flow by ∼20%, reduced GFR by ∼10% and proteinuria by ∼25%. These effects are analogous to those of a RAS inhibitor and it is noteworthy that patients in this study were already established on these agents, suggesting add-on benefits. Both doses of apelin also increased natriuresis and free water clearance. These effects are important given CKD is associated with salt and water retention, often necessitating diuretic treatment. Finally, apelin improved cardiovascular function in these patients with reductions in blood pressure and systemic vascular resistance of ∼5% and ∼10%, respectively, and an increase in cardiac output of ∼15%. Overall, if these short-term effects were maintained longer-term, 1 would anticipate improved patient outcomes. Long-acting oral analogs are now needed for future studies.

##### b Diabetic kidney disease

Diabetes mellitus is the commonest cause of CKD. The role of the apelin system in diabetic nephropathy is controversial. Preclinical models of type 1 (T1DM) and type 2 (T2DM) diabetes mellitus show that renal expression of the apelin system is altered, with evidence of both upregulation and downregulation of apelin and apelin receptor mRNA and protein.^336^^,^^338^^,^^340^^,^^381^, ^382^, ^383^, ^384^, ^385^ Studies of ELA find reduced expression of mRNA and protein.^341^^,^^342^ In human kidney tissue from patients with T2DM and nephropathy, apelin receptor protein was found to be increased.^340^

Data are conflicting on the effects of apelin receptor agonism in diabetic nephropathy. In both high-glucose-treated mesangial cells and podocytes, apelin reverses the upregulation of profibrotic genes.^338^^,^^384^ In models of T1DM, apelin improved glomerular structural changes, reducing inflammation and proteinuria.^336^^,^^337^^,^^381^^,^^382^ These effects appear to be mediated through endothelial regulation of kidney blood flow. In the streptozotocin mouse model of T1DM and nephropathy, ELA knockdown results in more kidney injury compared with wild type animals.^342^ In this model, 6 months of ELA treatment improved blood glucose control, increased plasma insulin level, and reduced serum creatinine and albuminuria by preserving podocyte structure and reducing inflammation and fibrosis.^385^

Models of T2DM show less consistent benefits. In the KK-Ay mouse model, apelin increased albuminuria and progressed nephropathy through effects on renal hemodynamics and podocyte apoptosis and autophagy.^340^^,^^386^, ^387^, ^388^ However, in 2 studies of the mouse model of type 2 diabetes model, ELA preserved glomerular architecture, and reduced albuminuria and fibrosis.^341^^,^^342^ In vitro studies demonstrated that in endothelial cells the effects of ELA were mediated via the apelin receptor and regulation of the AMPK/NOD-like receptor protein 3 pathway.^342^ In this model, ELA upregulated tubular autophagy.^341^

##### c Kidney fibrosis

Any kidney injury, left unchecked, leads to irreversible glomerular and tubulointerstitial fibrosis with progression to kidney failure and the need for dialysis or a kidney transplant. Multiple mechanisms underlie fibrosis development. Epithelial-to-mesenchymal transition (EMT), predominantly driven by TGF*β*, is a key contributor. ANG II also promotes fibrosis by activation of the ANG II-type 1 (AT_1_) receptor, and this can be mitigated by treatment with RAS blockers. The apelin system inhibits fibrosis, and, within the kidney, this appears to be mediated by inhibiting EMT and through crosstalk with the RAS.

In vitro, apelin suppresses TGF*β*-induced EMT in human proximal tubular epithelial cells^389^^,^^390^ and EMT in vascular endothelial cells.^391^ In the unilateral ureteric obstruction mouse model, apelin decreases transcription of the extracellular matrix proteins fibronectin, collagen I and collagen III, and attenuates injury-induced fibrosis. Here, apelin also prevents upregulation of TGF*β* and *α*-smooth muscle actin (a marker of myofibroblasts) and downregulation of E-cadherin (an epithelial cell adhesion protein) and laminin (a component of the tubular basement membrane).^389^

The intrarenal RAS regulates blood pressure, salt and water balance and reciprocally regulates expression of the apelin system. In vitro, treatment of collecting duct cells with ELA-32 reduces expression of (pro)renin receptor and renin protein.^392^ Knockout of (pro)renin receptor in mouse collecting duct or nephron increases apelin and ELA mRNA although the effects on apelin receptor protein expression depend on the location of the knockout.^392^ In the mouse unilateral ureteric obstruction model, treatment with the AT_1_ receptor blocker losartan enhances expression of apelin mRNA, increases activation of the Akt/endothelial nitric oxide synthase pathway, and reduces myofibroblast accumulation and kidney fibrosis.^393^ Cotreatment with the apelin receptor antagonist F13A or L-N^*G*^-monomethylarginine (a nitric oxide synthase inhibitor) abrogates these effects, suggesting that the antifibrotic effect of losartan may partly be mediated by crosstalk with the apelin system.

ELA also reduces kidney fibrosis. In the Dahl salt-sensitive rat, ELA overexpression or ELA-32 infusion suppresses salt-induced hypertension, kidney inflammation, and fibrosis.^392^^,^^394^ These effects appear to be mediated via suppressing the intrarenal RAS.^392^ ELA infusion was also more effective than apelin in lowering blood pressure, protecting against cardiorenal dysfunction and reducing fibrosis in salt-fed spontaneously hypertensive rats by altering the balance of ACE/ACE2/neprilysin and thus reducing RAS activation.^311^ The renoprotective and antifibrotic effects of ELA also appear to be mediated by inhibiting NADPH oxidase/reactive oxygen species/NOD-like receptor protein 3 inflammasome, although interestingly these effects appear to be independent of the apelin receptor.^395^

#### 4 Disorders of fluid homeostasis

##### a Syndrome of inappropriate antidiuresis

Syndrome of inappropriate antidiuresis (SIAD) is a common cause of hyponatremia due to an inappropriately high plasma AVP relative to osmolality, and thus inappropriate water reabsorption. It may be due to CNS disorders, drugs, lung disease, or malignancy, and the resulting hyponatremia can be mild and asymptomatic or, at worst, life-threatening with seizures and coma. Plasma copeptin is used as a surrogate measure of AVP because of its increased stability. Clinical studies find that in comparison to health, patients with SIAD have modestly elevated plasma apelin and the apelin:copeptin ratio is lower than expected.^396^ Whether apelin could be used therapeutically to restore salt and water balance in SIAD is an important question. A rat model of SIAD examined the effect of the apelin receptor agonist, LIT01-196, and established that treatment significantly increased urine output, reduced urine osmolality and increased plasma sodium concentration.^19^ The results of a clinical study examining the effect of apelin in artificially induced SIAD are awaited with interest (NCT06277336).

##### b Polyuria/polydipsia syndromes

Polyuria/polydipsia syndromes are characterized by an increased urine output of >50 mL/kg/day coupled with excessive (>3 L/day) fluid intake. These occur either because of a AVP disorder (AVP deficiency or resistance [AVP-D or AVP-R, respectively], previously known as cranial or nephrogenic diabetes insipidus) or primary polydipsia. A disrupted apelin–AVP balance occurs in these conditions, with parallel changes in circulating peptide concentrations.^397^ Compared with health, circulating apelin is lower in primary polydipsia and complete AVP-D, and is elevated in AVP-R. The apelin:copeptin ratio is unchanged in primary polydipsia compared with health (supporting normal water homeostasis) but is significantly decreased in AVP-D and increased in AVP-R. Accurate discrimination between primary polydipsia and AVP-D is critical and currently is based on copeptin level. At present plasma apelin does not appear to offer additional discriminatory value.^398^

## X Conclusions and perspectives

The apelin receptor is activated by 2 endogenous peptides, apelin and ELA. Acute investigational clinical studies (forearm and systemic infusions) using [Pyr^1^]apelin-13 as a challenge compound, have established a pivotal role for this peptide in a range of physiological processes, including cardiovascular, metabolic, and renal regulation. The apelin receptor has emerged as a promising pharmacological target owing to the maintenance of these beneficial actions by [Pyr^1^]apelin-13 in patients with PAH, HF (through enhanced cardiac contractility and reduction in peripheral resistance), chronic renal failure (increasing glomerular filtration rate while reducing proteinuria), and T2DM (improves insulin sensitivity and lipid metabolism) where there is still an unmet need for new therapies. In these conditions, plasma apelin levels (and frequently tissue levels where these have been measured) are reduced, providing the therapeutic rationale for replacing the missing endogenous peptide with a synthetic agonist. Apelin levels decline with age. In patients with peripheral artery disease the decline correlated with clinical severity and can be reversed in aged mice with apelin treatment.^399^ Muscle contraction was reduced with age in humans and in mice apelin reverses age-associated sarcopenia.^217^ Importantly, a small molecule apelin agonist, in an innovative clinical development, has expanded these indications; the prevention of muscle atrophy in hospitalized patients confined to bed rest.^259^ Inducing loss of weight and fat but preserving muscle was the rationale for combining a GLP/GIP agonist with an apelin agonist. This combination has been shown to be effective in obese mice but tantalizingly remains to be established in clinical trials.

New therapeutic strategies continue to emerge. Extracellular vesicles (naturally secreted nanoparticles) have been engineered to display apelin with the C-terminus exposed and functional for receptor binding. In addition, the vesicles were engineered to display a PAH-targeting peptide CARSKNKDC, to bind to heparan sulfate overexpressed on PAH endothelial cells. Sugen-hypoxia PAH mouse model and reversed vascular remodeling and improved cardiac function.^400^

X-ray crystallography and cryo-EM, combined with molecular modeling, have demonstrated apelin and ELA can bind to the apelin receptor through different amino acids, with signaling via distinct pathways. Intriguingly, a patient with a rare genetic disease, who possessed a single amino acid variant, was able to bind apelin but not ELA, suggesting a potential link with a pathophysiological condition. The relative importance of ELA signaling to apelin in humans remains to be established, particularly as current receptor antagonists block both ligands. The development of selective ligands that distinguish between the action of the 2 peptides will be needed to resolve this question and whether there is merit in the further development of ELA selective agents.

Recent advances in structural biology and biased agonism are reshaping the future of apelin receptor pharmacology. One of the most transformative developments is the discovery of G protein-biased agonists, one of which, MM07, has been tested in the clinic, that preferentially activate beneficial signaling pathways, while reducing desensitization of the receptor in humans. Desensitization is a major factor in the development of drug tolerance, especially for chronic treatments using agonists targeting Family A GPCRs such as the opioid receptors. As proof of this concept, the first ground-breaking agonist specifically selected to be G protein-biased (oliceridine, targeting the *μ* opiate receptor)^401^ was approved by the US Food and Drug Administration in 2020 for acute pain. Recent compounds such as CMF-019, ANPA-0073, and NXE’065 exemplify this new generation of biased agonists.

In common with angiotensin AT_1_ receptors, the apelin receptor can also be activated by mechanical forces, such as stretch on the cell membrane, when levels of the endogenous peptide are reduced. G protein-biased apelin agonists are predicted to avoid adverse effects associated with *β*-arrestin signaling linked to cardiac hypertrophy, which may limit the utility of balanced agonists. Testing this hypothesis will require head-to-head comparison in translational animal models and in clinical studies to understand the optimum strategy to maintain efficacy during chronic dosing.

Further clinical trials are urgently required with existing peptide and small molecule agonists in order to exploit therapeutic targets identified in acute studies with [Pyr^1^]apelin-13. Structural insights have revealed key residues responsible for signaling bias, enabling the rational design of ligands that engage specific receptor conformations for minimizing off-target effects and improving clinical outcomes.

## Conflict of interest

The authors declare no conflicts of interest.

## References

[1] O'Dowd B.F., Heiber M., Chan A. (Dec 22 1993). A human gene that shows identity with the gene encoding the angiotensin receptor is located on chromosome 11. Gene.

[2] Tatemoto K., Hosoya M., Habata Y. (Oct 20 1998). Isolation and characterization of a novel endogenous peptide ligand for the human APJ receptor. Biochem Biophys Res Commun.

[3] Masri B., Knibiehler B., Audigier Y. (2005). Apelin signalling: a promising pathway from cloning to pharmacology. Cell Signal.

[4] Tatemoto K., Takayama K., Zou M.X. (2001). The novel peptide apelin lowers blood pressure via a nitric oxide-dependent mechanism. Regul Pept.

[5] Maguire J.J., Kleinz M.J., Pitkin S.L., Davenport A.P. (2009). [Pyr1]apelin-13 identified as the predominant apelin isoform in the human heart: vasoactive mechanisms and inotropic action in disease. Hypertension.

[6] Davenport A.P., Alexander S.P.H., Sharman J.L. (2013). International union of basic and Clinical Pharmacology. LXXXVIII. G protein-coupled receptor list: recommendations for new pairings with cognate ligands. Pharmacol Rev.

[7] Pitkin S.L., Maguire J.J., Bonner T.I., Davenport A.P. (2010). International Union of Basic and Clinical Pharmacology. LXXIV. Apelin receptor nomenclature, distribution, pharmacology, and function. Pharmacol Rev.

[8] Chng S.C., Ho L., Tian J., Reversade B. (2013). ELABELA: a hormone essential for heart development signals via the apelin receptor. Dev Cell.

[9] Pauli A., Norris M.L., Valen E. (2014). Toddler: an embryonic signal that promotes cell movement via Apelin receptors. Science.

[10] Hosoya M., Kawamata Y., Fukusumi S. (2000). Molecular and functional characteristics of APJ. Tissue distribution of mRNA and interaction with the endogenous ligand apelin. J Biol Chem.

[11] Fan X., Zhou N., Zhang X. (2003). Structural and functional study of the apelin-13 peptide, an endogenous ligand of the HIV-1 coreceptor, APJ. Biochemistry.

[12] Medhurst A.D., Jennings C.A., Robbins M.J. (2003). Pharmacological and immunohistochemical characterization of the APJ receptor and its endogenous ligand apelin. J Neurochem.

[13] Yang P., Read C., Kuc R.E. (2017). Elabela/Toddler is an endogenous agonist of the apelin APJ receptor in the adult cardiovascular system, and exogenous administration of the peptide compensates for the downregulation of its expression in pulmonary arterial hypertension. Circulation.

[14] Williams T.L., Macrae R.G.C., Kuc R.E., Brown A.J.H., Maguire J.J., Davenport A.P. (2023). Expanding the apelin receptor pharmacological toolbox using novel fluorescent ligands. Front Endocrinol.

[15] Katugampola S.D., Maguire J.J., Matthewson S.R., Davenport A.P. (2001). [(125)I]-(Pyr(1))Apelin-13 is a novel radioligand for localizing the APJ orphan receptor in human and rat tissues with evidence for a vasoconstrictor role in man. Br J Pharmacol.

[16] Hamada J., Kimura J., Ishida J. (2008). Evaluation of novel cyclic analogues of apelin. Int J Mol Med.

[17] McKeown S.C., Zecri F.J., Fortier E. (2014). The design and implementation of a generic lipopeptide scanning platform to enable the identification of “locally acting” agonists for the apelin receptor. Bioorg Med Chem Lett.

[18] Trân K., Van Den Hauwe R., Sainsily X. (2021). Constraining the side chain of C-terminal amino acids in Apelin-13 greatly increases affinity, modulates signaling, and improves the pharmacokinetic profile. J Med Chem.

[19] Flahault A., Keck M., Girault-Sotias P.E. (2021). LIT01-196, a metabolically stable Apelin-17 analog, normalizes blood pressure in hypertensive DOCA-salt rats via a NO synthase-dependent mechanism. Front Pharmacol.

[20] Flahault A., Girault-Sotias P.E., Keck M. (2021). A metabolically stable apelin-17 analog decreases AVP-induced antidiuresis and improves hyponatremia. Nat Commun.

[21] Fernandez K.X., Fischer C., Vu J. (2021). Metabolically stable apelin-analogues, incorporating cyclohexylalanine and homoarginine, as potent apelin receptor activators. RSC Med Chem.

[22] Read C., Yang P., Kuc R.E. (2020). Apelin peptides linked to anti-serum albumin domain antibodies retain affinity in vitro and are efficacious receptor agonists in vivo. Basic Clin Pharmacol Toxicol.

[23] Ma Y., Ding Y., Song X. (2020). Structure-guided discovery of a single-domain antibody agonist against human apelin receptor. Sci Adv.

[24] Deng C., Chen H., Yang N., Feng Y., Hsueh A.J.W. (2015). Apela regulates fluid homeostasis by binding to the APJ receptor to activate Gi signaling. J Biol Chem.

[25] Wang C., Xiong M., Yang C. (2020). PEGylated and acylated elabela analogues show enhanced receptor binding, prolonged stability, and remedy of acute kidney injury. J Med Chem.

[26] Brame A.L., Maguire J.J., Yang P. (2015). Design, characterization, and first-in-human study of the vascular actions of a novel biased apelin receptor agonist. Hypertension.

[27] Tran K., Sainsily X., Côté J. (2022). Size-reduced macrocyclic analogues of [Pyr1]-apelin-13 showing negative Gα12 bias still produce prolonged cardiac effects. J Med Chem.

[28] Williams T.L., Verdon G., Kuc R.E. (2024). Structural and functional determination of peptide versus small molecule ligand binding at the apelin receptor. Nat Commun.

[29] Iturrioz X., Alvear-Perez R., De Mota N. (2010). Identification and pharmacological properties of E339-3D6, the first nonpeptidic apelin receptor agonist. FASEB J.

[30] Khan P., Maloney P.R., Hedrick M. (2011). Functional agonists of the apelin (APJ) receptor. Probe Reports from the NIH Molecular Libraries Program, dataset. https://www.ncbi.nlm.nih.gov/books/NBK98921/.

[31] Narayanan S., Wang S., Vasukuttan V. (2021). Pyrazole agonist of the apelin receptor improves symptoms of metabolic syndrome in mice. J Med Chem.

[32] Narayanan S., Dai D., Vyas Devambatla R.K. (2022). Synthesis and characterization of an orally bioavailable small molecule agonist of the apelin receptor. Bioorg Med Chem.

[33] Myers M.C., Bilder D.M., Cavallaro C.L. (2020). Discovery and SAR of aryl hydroxy pyrimidinones as potent small molecule agonists of the GPCR APJ. Bioorg Med Chem Lett.

[34] Gargalovic P., Wong P., Onorato J. (2021). In vitro and in vivo evaluation of a small-molecule APJ (apelin receptor) agonist, BMS-986224, as a potential treatment for heart failure. Circ Heart Fail.

[35] Pi Z., Johnson J.A., Meng W. (2021). Identification of 6-hydroxypyrimidin-4(1H)-one-3-carboxamides as potent and orally active APJ receptor agonists. ACS Med Chem Lett.

[36] Johnson J.A., Kim S.H., Jiang J. (2021). Discovery of a hydroxypyridinone APJ receptor agonist as a clinical candidate. J Med Chem.

[37] Meng W., Pi Z., Brigance R. (2021). Identification of a hydroxypyrimidinone compound (21) as a potent APJ receptor agonist for the potential treatment of heart failure. J Med Chem.

[38] Su S., Clarke A., Han Y. (2019). Biphenyl acid derivatives as APJ receptor agonists. J Med Chem.

[39] Ason B., Chen Y., Guo Q. (2020). Cardiovascular response to small-molecule APJ activation. JCI Insight.

[40] Yue Y., Liu L., Wu L.J. (2022). Structural insight into apelin receptor-G protein stoichiometry. Nat Struct Mol Biol.

[41] Read C., Fitzpatrick C.M., Yang P. (2016). Cardiac action of the first G protein biased small molecule apelin agonist. Biochem Pharmacol.

[42] Wang W.W., Ji S.Y., Zhang W. (2024). Structure-based design of non-hypertrophic apelin receptor modulator. Cell.

[43] Sun Q., Tian X., Tan L. (2025). Multiscale biased chemical space remodeling for developing APLNR agonists with anti-HFpEF efficacy. Proc Natl Acad Sci U S A.

[44] Zhou N., Fang J., Acheampong E., Mukhtar M., Pomerantz R.J. (2003). Binding of ALX40-4C to APJ, a CNS-based receptor, inhibits its utilization as a co-receptor by HIV-1. Virology.

[45] Le Gonidec S., Chaves-Almagro C., Bai Y. (2017). Protamine is an antagonist of apelin receptor, and its activity is reversed by heparin. FASEB J.

[46] Macaluso N.J.M., Pitkin S.L., Maguire J.J., Davenport A.P., Glen R.C. (2011). Discovery of a competitive apelin receptor (APJ) antagonist. ChemMedChem.

[47] Davenport A.P., Glen R.C., Maguire J.J. Compounds for use as apelin receptor antagonists. International patent WO2019193355A1; 2019. https://patents.google.com/patent/US20210155659A1/en1.

[48] Maloney P.R., Khan P., Hedrick M. (2012). Discovery of 4-oxo-6-((pyrimidin-2-ylthio)methyl)-4H-pyran-3-yl 4-nitrobenzoate (ML221) as a functional antagonist of the apelin (APJ) receptor. Bioorg Med Chem Lett.

[49] McAnally D., Siddiquee K., Gomaa A. (2018). Repurposing antimalarial aminoquinolines and related compounds for treatment of retinal neovascularization. PLoS One.

[50] Shi S., Guan C., Zhang F. (2023). Discovery of G-protein biased APJ agonist small molecule for pulmonary diseases [abstract]. Am J Respir Crit Care Med.

[51] Xie F., Lv D., Chen L. (2014). ELABELA: a novel hormone in cardiac development acting as a new endogenous ligand for the APJ receptor. Acta Biochim Biophys Sin.

[52] Murza A., Sainsily X., Coquerel D. (2016). Discovery and structure-activity relationship of a bioactive fragment of ELABELA that modulates vascular and cardiac functions. J Med Chem.

[53] Read C., Nyimanu D., Williams T.L. (2019). International Union of Basic and Clinical Pharmacology. CVII. Structure and pharmacology of the apelin receptor with a recommendation that Elabela/Toddler is a second endogenous peptide ligand. Pharmacol Rev.

[54] Japp A.G., Cruden N.L., Amer D.A.B. (2008). Vascular effects of apelin in vivo in man. J Am Coll Cardiol.

[55] Barnes G., Japp A.G., Newby D.E. (2010). Translational promise of the apelin—APJ system. Heart.

[56] Japp A.G., Newby D.E. (2008). The apelin-APJ system in heart failure: pathophysiologic relevance and therapeutic potential. Biochem Pharmacol.

[57] Japp A.G., Cruden N.L., Barnes G. (2010). Acute cardiovascular effects of apelin in humans: potential role in patients with chronic heart failure. Circulation.

[58] Japp A.G., Newby D.E. (2016). Unlocking the therapeutic potential of apelin. Hypertension.

[59] Barnes G.D., Alam S., Carter G. (2013). Sustained cardiovascular actions of APJ agonism during renin-angiotensin system activation and in patients with heart failure. Circ Heart Fail.

[60] Yang P., Kuc R.E., Brame A.L. (2017). [Pyr1]apelin-13(1–12) is a biologically active ACE2 metabolite of the endogenous cardiovascular peptide [Pyr1]Apelin-13. Front Neurosci.

[61] Brame A.L. (2015).

[62] Brash L., Barnes G.D., Brewis M.J. (2018). Short-term hemodynamic effects of apelin in patients with pulmonary arterial hypertension. JACC Basic Transl Sci.

[63] Gourdy P., Cazals L., Thalamas C. (2018). Apelin administration improves insulin sensitivity in overweight men during hyperinsulinaemic-euglycaemic clamp. Diabetes Obes Metab.

[64] Schinzari F., Veneziani A., Mores N. (2017). Beneficial effects of apelin on vascular function in patients with central obesity. Hypertension.

[65] Sulentic P. (2022).

[66] Chapman F.A., Melville V., Godden E. (2024). Cardiovascular and renal effects of apelin in chronic kidney disease: a randomised, double-blind, placebo-controlled, crossover study. Nat Commun.

[67] Novartis (2019). A randomised, subject and investigator-blind, placebo-controlled study of CLR325 in chronic stable heart failure patients. https://www.novctrd.com/ctrdweb/trialresult/trialresults/pdf?trialResultId=17555.

[68] Winkle P., Goldsmith S., Koren M.J. (2023). A first-in-human study of AMG 986, a Novel Apelin receptor agonist, in healthy subjects and heart failure patients. Cardiovasc Drugs Ther.

[69] Trivedi A., Kiang Y.H., Saw R.E. (2022). Evaluation of the pharmacokinetics and safety of AMG 986 tablet and capsule formulations in healthy adult subjects: a phase I, open-label, randomized study. Drugs R D.

[70] Trivedi A., Mather O., Vega S., Hutton S., Hellawell J., Lee E. (2022). A Phase I, open-label, single-dose study to evaluate the pharmacokinetics, safety, and tolerability of AMG 986 in healthy Japanese subjects. Drugs R D.

[71] Trivedi A., Mather O., Vega S., Hutton S., Hellawell J., Lee E. (2022). A phase 1, open-label study to evaluate the effect of food and concomitant itraconazole administration on the pharmacokinetics of AMG 986 in healthy subjects. Clin Pharmacol Drug Dev.

[72] Trivedi A., Mather O., Vega S., Simiens M.A., Hellawell J., Lee E. (2022). Effect of severe renal impairment on the safety, tolerability, and pharmacokinetics of AMG 986. Drugs R D.

[73] Bioage BioAge announces first patient dosed in the STRIDES phase 2 clinical trial evaluating azelaprag as a novel treatment for obesity in combination with tirzepatide. https://ir.bioagelabs.com/news-releases/news-release-details/bioage-announces-first-patient-dosed-strides-phase-2-clinical.

[74] Bioage BioAge labs announces discontinuation of STRIDES phase 2 clinical trial evaluating azelaprag in combination with tirzepatide for the treatment of obesity. https://ir.bioagelabs.com/news-releases/news-release-details/bioage-labs-announces-discontinuation-strides-phase-2-clinical.

[75] Bach M., Shi S., Zhang J. (2023). A First-in-human single/multiple ascending dose study of ANPA-0073, a novel small molecule G-protein biased apelin receptor agonist, in healthy volunteers [abstract]. Am J Respir Crit Care Med.

[76] Davenport A.P., Brame A.L., Kuc R.E. (2018). First in human study of a novel biased apelin receptor ligand, MM54, A G-alpha (i) agonist/beta-arrestin antagonist. Circ Res.

[77] Marsault E., Llorens-Cortes C., Iturrioz X. (2019). The apelinergic system: a perspective on challenges and opportunities in cardiovascular and metabolic disorders. Ann N Y Acad Sci.

[78] de Oliveira A.A., Vergara A., Wang X., Vederas J.C., Oudit G.Y. (2022). Apelin pathway in cardiovascular, kidney, and metabolic diseases: therapeutic role of apelin analogs and apelin receptor agonists. Peptides.

[79] Wagenaar G.T.M., Moll G.N. (2025). Advances in the therapeutic potentials of ligands of the apelin receptor APJ. Eur J Pharmacol.

[80] Couvineau P., Llorens-Cortes C. (2025). Metabolically stable apelin analogs: development and functional role in water balance and cardiovascular function. Clin Sci.

[81] Gao S., Chen H. (2023). Therapeutic potential of apelin and Elabela in cardiovascular disease. Biomed Pharmacother.

[82] Zhong J.C., Zhang Z.Z., Wang W., McKinnie S.M.K., Vederas J.C., Oudit G.Y. (2017). Targeting the apelin pathway as a novel therapeutic approach for cardiovascular diseases. Biochim Biophys Acta Mol Basis Dis.

[83] Chatterjee P., Gheblawi M., Wang K., Vu J., Kondaiah P., Oudit G.Y. (2020). Interaction between the apelinergic system and ACE2 in the cardiovascular system: therapeutic implications. Clin Sci.

[84] Wagenaar G.T.M., Moll G.N. (2025). Clinical significance of intervention in the renin-angiotensin-aldosterone-apelinergic system. Eur J Pharmacol.

[85] Naldi L., Peri A., Fibbi B. (2025). Apelin/APJ: another player in the cancer biology network. Int J Mol Sci.

[86] Chen J., Li Z., Zhao Q., Chen L. (2022). Roles of apelin/APJ system in cancer: biomarker, predictor, and emerging therapeutic target. J Cell Physiol.

[87] Jafarzadeh A., Naseri B., Khorramdelazad H. (2024). Reciprocal interactions between apelin and noncoding RNAs in cancer progression. Cell Biochem Funct.

[88] Tian Y., Chen R., Jiang Y., Bai B., Yang T., Liu H. (2020). The protective effects and mechanisms of Apelin/APJ system on ischemic stroke: a promising therapeutic target. Front Neurol.

[89] Huang M., Zhang Y., Shen Y., Xu Y., Liu X. (2025). Apelin-13 can regulate adipose-derived mesenchymal stem cells to improve traumatic brain injury. Mol Cell Neurosci.

[90] Huang Z., Liu Q., Guo Q., Gao J., Zhang L., Li L. (2025). Effects and mechanisms of Apelin in treating central nervous system diseases. Neuroscience.

[91] Zhang Y., Jiang W., Sun W. (2023). Neuroprotective roles of Apelin-13 in neurological diseases. Neurochem Res.

[92] Li A., Zhao Q., Chen L., Li Z. (2023). Apelin/APJ system: an emerging therapeutic target for neurological diseases. Mol Biol Rep.

[93] Li J., Chen Z., Chen J., Yu Y. (2022). The beneficial roles of apelin-13/APJ system in cerebral ischemia: pathogenesis and therapeutic strategies. Front Pharmacol.

[94] Wan T., Fu M., Jiang Y., Jiang W., Li P., Zhou S. (2022). Research progress on mechanism of neuroprotective roles of Apelin-13 in prevention and treatment of Alzheimer’s disease. Neurochem Res.

[95] Kamińska K., Borzuta H., Buczma K., Cudnoch-Jędrzejewska A. (2024). Neuroprotective effect of apelin-13 and other apelin forms-a review. Pharmacol Rep.

[96] Behrouzifar S., Esmaily H. (2024). The biological efficacy of Apelin against focal transient cerebral ischemia-reperfusion injury. A systematic review and meta-analysis of animal studies. Brain Res.

[97] Luo H., Gu X., Tong G., Han L. (2022). Research progress of apelin in acute ischemic brain injury. Am J Transl Res.

[98] Li C., Cheng H., Adhikari B.K. (2022). The role of apelin-APJ system in diabetes and obesity. Front Endocrinol.

[99] Castan-Laurell I., Dray C., Valet P. (2021). The therapeutic potentials of apelin in obesity-associated diseases. Mol Cell Endocrinol.

[100] Wen R., Huang R., Xu K., Cheng Y., Yi X. (2023). Beneficial effects of Apelin-13 on metabolic diseases and exercise. Front Endocrinol.

[101] Wang Z., Yu D., Wang M. (2015). Elabela-apelin receptor signaling pathway is functional in mammalian systems. Sci Rep.

[102] Zhang Y., Wang Y., Lou Y. (2018). Elabela, a newly discovered APJ ligand: similarities and differences with Apelin. Peptides.

[103] Seo K., Parikh V.N., Ashley E.A. (2020). Stretch-induced biased signaling in angiotensin II Type 1 and apelin receptors for the mediation of cardiac contractility and hypertrophy. Front Physiol.

[104] Dagamajalu S., Rex D.A.B., Suchitha G.P., Rai A.B., Rainey J.K., Prasad T.S.K. (2022). The network map of Elabela signaling pathway in physiological and pathological conditions. J Cell Commun Signal.

[105] Murali S., Aradhyam G.K. (2023). Structure-function relationship and physiological role of apelin and its G protein coupled receptor. Biophys Rev.

[106] Iturrioz X., Gerbier R., Leroux V., Alvear-Perez R., Maigret B., Llorens-Cortes C. (2010). By interacting with the C-terminal Phe of apelin, Phe255 and Trp259 in helix VI of the apelin receptor are critical for internalization. J Biol Chem.

[107] Gerbier R., Leroux V., Couvineau P. (2015). New structural insights into the apelin receptor: identification of key residues for apelin binding. FASEB J.

[108] Ma Y., Yue Y., Ma Y. (2017). Structural basis for apelin control of the human apelin receptor. Structure.

[109] Herrera L.P.T., Andreassen S.N., Caroli J. (2025). GPCRdb in 2025: adding odorant receptors, data mapper, structure similarity search and models of physiological ligand complexes. Nucleic Acids Res.

[110] Ballesteros J.A., Weinstein H. (1995). Integrated methods for the construction of three-dimensional models and computational probing of structure-function relations in G protein-coupled receptors. Methods Neurosci.

[111] Bonde M.M., Hansen J.T., Sanni S.J. (2010). Biased signaling of the angiotensin II type 1 receptor can be mediated through distinct mechanisms. PLoS One.

[112] Shao Z., Shen Q., Yao B. (2022). Identification and mechanism of G protein-biased ligands for chemokine receptor CCR1. Nat Chem Biol.

[113] García-Nafría J., Tate C.G. (2020). Cryo-electron microscopy: moving beyond X-ray crystal structures for drug receptors and drug development. Annu Rev Pharmacol Toxicol.

[114] Trân K., Murza A., Sainsily X. (2021). Structure-activity relationship and bioactivity of short analogues of ELABELA as agonists of the apelin receptor. J Med Chem.

[115] Schöneberg T., Liebscher I. (2021). Mutations in G protein-coupled receptors: mechanisms, pathophysiology and potential therapeutic approaches. Pharmacol Rev.

[116] Zeng X.X., Wilm T.P., Sepich D.S., Solnica-Krezel L. (2007). Apelin and its receptor control heart field formation during zebrafish gastrulation. Dev Cell.

[117] Scott I.C., Masri B., D’Amico L.A. (2007). The g protein-coupled receptor agtrl1b regulates early development of myocardial progenitors. Dev Cell.

[118] Shin K., Pandey A., Liu X.Q., Anini Y., Rainey J.K. (2013). Preferential apelin-13 production by the proprotein convertase PCSK3 is implicated in obesity. FEBS Open Bio.

[119] Fischer C., Lamer T., Wang W. (2019). Plasma kallikrein cleaves and inactivates apelin-17: palmitoyl- and PEG-extended apelin-17 analogs as metabolically stable blood pressure-lowering agents. Eur J Med Chem.

[120] Murza A., Belleville K., Longpré J.M., Sarret P. (2014). Marsault É. Stability and degradation patterns of chemically modified analogs of apelin-13 in plasma and cerebrospinal fluid. Biopolymers.

[121] Vickers C., Hales P., Kaushik V. (2002). Hydrolysis of biological peptides by human angiotensin-converting enzyme-related carboxypeptidase. J Biol Chem.

[122] Wang W., McKinnie S.M.K., Farhan M. (2016). Angiotensin-converting enzyme 2 metabolizes and partially inactivates Pyr-Apelin-13 and Apelin-17: physiological effects in the cardiovascular system. Hypertension.

[123] De Mota N., Reaux-Le Goazigo A., El Messari S. (2004). Apelin, a potent diuretic neuropeptide counteracting vasopressin actions through inhibition of vasopressin neuron activity and vasopressin release. Proc Natl Acad Sci U S A.

[124] Zhen E.Y., Higgs R.E., Gutierrez J.A. (2013). Pyroglutamyl apelin-13 identified as the major apelin isoform in human plasma. Anal Biochem.

[125] Mesmin C., Fenaille F., Becher F., Tabet J.C., Ezan E. (2011). Identification and characterization of apelin peptides in bovine colostrum and milk by liquid chromatography-mass spectrometry. J Proteome Res.

[126] Shin K., Chapman N.A., Sarker M. (2017). Bioactivity of the putative apelin proprotein expands the repertoire of apelin receptor ligands. Biochim Biophys Acta Gen Subj.

[127] Nyimanu D., Kay R.G., Sulentic P. (2019). Development and validation of an LC-MS/MS method for detection and quantification of in vivo derived metabolites of [Pyr1]apelin-13 in humans. Sci Rep.

[128] Pitkin S.L., Maguire J.J., Kuc R.E., Davenport A.P. (2010). Modulation of the apelin/APJ system in heart failure and atherosclerosis in man. Br J Pharmacol.

[129] Nyimanu D., Chapman F.A., Gallacher P.J. (2022). Apelin is expressed throughout the human kidney, is elevated in chronic kidney disease & associates independently with decline in kidney function. Br J Clin Pharmacol.

[130] Soave M., Briddon S.J., Hill S.J., Stoddart L.A. (2020). Fluorescent ligands: bringing light to emerging GPCR paradigms. Br J Pharmacol.

[131] Stoddart L.A., White C.W., Nguyen K., Hill S.J., Pfleger K.D.G. (2016). Fluorescence- and bioluminescence-based approaches to study GPCR ligand binding. Br J Pharmacol.

[132] Vernall A.J., Hill S.J., Kellam B. (2014). The evolving small-molecule fluorescent-conjugate toolbox for Class A GPCRs. Br J Pharmacol.

[133] Sridharan R., Zuber J., Connelly S.M., Mathew E., Dumont M.E. (2014). Fluorescent approaches for understanding interactions of ligands with G protein coupled receptors. Biochim Biophys Acta.

[134] Margathe J.F., Iturrioz X., Regenass P. (2016). Convenient access to fluorescent probes by chemoselective acylation of Hydrazinopeptides: application to the synthesis of the first Far-red ligand for apelin receptor imaging. Chemistry.

[135] Maujean T., Wagner P., Valencia C. (2023). Rapid and highly selective fluorescent labeling of peptides via a Thia-Diels-Alder cycloaddition: application to apelin. Bioconjug Chem.

[136] Ho L., Tan S.Y.X., Wee S. (2015). ELABELA is an endogenous growth factor that sustains hESC self-renewal via the PI3K/AKT pathway. Cell Stem Cell.

[137] Földes G., Horkay F., Szokodi I. (2003). Circulating and cardiac levels of apelin, the novel ligand of the orphan receptor APJ, in patients with heart failure. Biochem Biophys Res Commun.

[138] Chen M.M., Ashley E.A., Deng D.X. (2003). Novel role for the potent endogenous inotrope apelin in human cardiac dysfunction. Circulation.

[139] Francia P., Salvati A., Balla C. (2007). Cardiac resynchronization therapy increases plasma levels of the endogenous inotrope apelin. Eur J Heart Fail.

[140] Bohm A., Snopek P., Tothova L. (2021). Association between apelin and atrial fibrillation in patients with high risk of ischemic stroke. Front Cardiovasc Med.

[141] Foris V., Kovacs G., Avian A. (2022). Apelin-17 to diagnose idiopathic pulmonary arterial hypertension: a biomarker study. Front Physiol.

[142] Chandra S.M., Razavi H., Kim J. (2011). Disruption of the apelin-APJ system worsens hypoxia-induced pulmonary hypertension. Arterioscler Thromb Vasc Biol.

[143] Goetze J.P., Rehfeld J.F., Carlsen J. (2006). Apelin: a new plasma marker of cardiopulmonary disease. Regul Pept.

[144] Cui C., Zhou H., Xu J. (2021). ELABELA acts as a protective biomarker in patients with atrial fibrillation. J Thorac Dis.

[145] Liu C., Xiong J., Yi X. (2024). Decreased plasma ELABELA level as a novel screening indicator for heart failure: a cohort and observational study. Sci Rep.

[146] Ma Z., Zhao L., Zhang Y.P., Zhong J.C., Yang X.C. (2021). Declined ELABELA plasma levels in hypertension patients with atrial fibrillation: a case control study. BMC Cardiovasc Disord.

[147] Małyszko J., Małyszko J.S., Koźminski P., Myśliwiec M. (2006). Apelin and cardiac function in hemodialyzed patients: possible relations?. Am J Nephrol.

[148] Leal V.O., Lobo J.C., Stockler-Pinto M.B. (2012). Apelin: a peptide involved in cardiovascular risk in hemodialysis patients?. Ren Fail.

[149] Malyszko J., Malyszko J.S., Pawlak K., Wolczynski S., Mysliwiec M. (2008). Apelin, a novel adipocytokine, in relation to endothelial function and inflammation in kidney allograft recipients. Transplant Proc.

[150] Kleinz M.J., Davenport A.P. (2005). Emerging roles of apelin in biology and medicine. Pharmacol Ther.

[151] Dogan I., Dogan T., Yetim M. (2018). Relation of serum ADMA, Apelin-13 and LOX-1 levels with inflammatory and echocardiographic parameters in hemodialysis patients. Ther Apher Dial.

[152] Kocer D., Karakukcu C., Ozturk F., Eroglu E., Kocyigit I. (2016). Evaluation of fibrosis markers: apelin and transforming growth factor-β1 in autosomal dominant polycystic kidney disease patients. Ther Apher Dial.

[153] Lacquaniti A., Chirico V., Lupica R. (2013). Apelin and copeptin: two opposite biomarkers associated with kidney function decline and cyst growth in autosomal dominant polycystic kidney disease. Peptides.

[154] Leierer J., Perco P., Hofer B. (2021). Coregulation analysis of mechanistic biomarkers in autosomal dominant polycystic kidney disease. Int J Mol Sci.

[155] Masoumi J., Jafarzadeh A., Khorramdelazad H., Abbasloui M., Abdolalizadeh J., Jamali N. (2020). Role of Apelin/APJ axis in cancer development and progression. Adv Med Sci.

[156] Grinstead C., Yoon S. (2022). Apelin, a circulating biomarker in cancer evaluation: a systematic review. Cancers.

[157] Feng M., Yao G., Yu H., Qing Y., Wang K. (2016). Tumor apelin, not serum apelin, is associated with the clinical features and prognosis of gastric cancer. BMC Cancer.

[158] Mao X., Zhu X., Pan T. (2024). Apelin (APLN) is a biomarker contributing to the diagnosis and prognosis of hepatocellular carcinoma. Sci Rep.

[159] Zhu Y., Zhang P., Huo X. (2024). Single-cell and spatial transcriptomics reveal apelin/APJ pathway’s role in microvessel formation and tumour progression in hepatocellular carcinoma. J Cell Mol Med.

[160] Chen H., Wong C.C., Liu D. (2019). APLN promotes hepatocellular carcinoma through activating PI3K/Akt pathway and is a druggable target. Theranostics.

[161] Pritchard N., Kaitu’u-Lino T.J., Gong S. (2018). ELABELA/APELA levels are not decreased in the maternal circulation or placenta among women with preeclampsia. Am J Pathol.

[162] Villie P., Lefevre G., Arrestier R., Rousseau A., Berkane N., Hertig A. (2019). ELABELA concentration is not decreased in maternal plasma before the onset of preeclampsia. Am J Obstet Gynecol.

[163] Deniz R., Baykus Y., Ustebay S., Ugur K., Yavuzkir Ş., Aydin S. (2019). Evaluation of elabela, apelin and nitric oxide findings in maternal blood of normal pregnant women, pregnant women with pre-eclampsia, severe pre-eclampsia and umbilical arteries and venules of newborns. J Obstet Gynaecol.

[164] Zhou L., Sun H., Cheng R., Fan X., Lai S., Deng C. (2019). ELABELA, as a potential diagnostic biomarker of preeclampsia, regulates abnormally shallow placentation via APJ. Am J Physiol Endocrinol Metab.

[165] Panaitescu B., Romero R., Gomez-Lopez N. (2020). ELABELA plasma concentrations are increased in women with late-onset preeclampsia. J Matern Fetal Neonatal Med.

[166] Georgiadou D., Boussata S., Ranzijn W.H.M. (2019). Peptide hormone ELABELA enhances extravillous trophoblast differentiation, but placenta is not the major source of circulating ELABELA in pregnancy. Sci Rep.

[167] Fuentes-Carrasco M., Ruíz-Román R., Savirón-Cornudella R., Pérez-Roncero G., López-Baena M.T., Pérez-López F.R. (2022). Systematic review and meta-analysis regarding maternal apelin in pregnant women with and without preeclampsia. Gynecol Endocrinol.

[168] Ho L., van Dijk M., Chye S.T.J. (2017). ELABELA deficiency promotes preeclampsia and cardiovascular malformations in mice. Science.

[169] Ishida J., Hashimoto T., Hashimoto Y. (2004). Regulatory roles for APJ, a seven-transmembrane receptor related to angiotensin-type 1 receptor in blood pressure in vivo. J Biol Chem.

[170] Charo D.N., Ho M., Fajardo G. (2009). Endogenous regulation of cardiovascular function by apelin-APJ. Am J Physiol Heart Circ Physiol.

[171] Kang Y., Kim J., Anderson J.P. (2013). Apelin-APJ signaling is a critical regulator of endothelial MEF2 activation in cardiovascular development. Circ Res.

[172] Scimia M.C., Hurtado C., Ray S. (2012). APJ acts as a dual receptor in cardiac hypertrophy. Nature.

[173] Kidoya H., Ueno M., Yamada Y. (2008). Spatial and temporal role of the apelin/APJ system in the caliber size regulation of blood vessels during angiogenesis. EMBO J.

[174] Kuba K., Zhang L., Imai Y. (2007). Impaired heart contractility in Apelin gene-deficient mice associated with aging and pressure overload. Circ Res.

[175] Freyer L., Hsu C.W., Nowotschin S. (2017). Loss of Apela peptide in mice causes low penetrance embryonic lethality and defects in early mesodermal derivatives. Cell Rep.

[176] Takakura N., Kidoya H. (2009). Maturation of blood vessels by haematopoietic stem cells and progenitor cells: involvement of apelin/APJ and angiopoietin/Tie2 interactions in vessel caliber size regulation. Thromb Haemost.

[177] D’Aniello C., Lonardo E., Iaconis S. (2009). G protein-coupled receptor APJ and its ligand apelin act downstream of Cripto to specify embryonic stem cells toward the cardiac lineage through extracellular signal-regulated kinase/p70S6 kinase signaling pathway. Circ Res.

[178] Wang I.N., Wang X., Ge X. (2012). Apelin enhances directed cardiac differentiation of mouse and human embryonic stem cells. PLoS One.

[179] Zeng X., Yu S.P., Taylor T., Ogle M., Wei L. (2012). Protective effect of apelin on cultured rat bone marrow mesenchymal stem cells against apoptosis. Stem Cell Res.

[180] Yu Q.C., Hirst C.E., Costa M. (2012). APELIN promotes hematopoiesis from human embryonic stem cells. Blood.

[181] Li M., Gou H., Tripathi B.K. (2015). An Apela RNA-containing negative feedback loop regulates p53-mediated apoptosis in embryonic stem cells. Cell Stem Cell.

[182] Chen X., Zhao Q., Li C. (2015). OP9-Lhx2 stromal cells facilitate derivation of hematopoietic progenitors both in vitro and in vivo. Stem Cell Res.

[183] Park J.S., Yang H.N., Yi S.W., Kim J.H., Park K.H. (2016). Neoangiogenesis of human mesenchymal stem cells transfected with peptide-loaded and gene-coated PLGA nanoparticles. Biomaterials.

[184] Zhang N.K., Cao Y., Zhu Z.M. (2016). Activation of endogenous cardiac stem cells by Apelin-13 in infarcted rat heart. Cell Transplant.

[185] Liang D., Han D., Fan W. (2016). Therapeutic efficacy of apelin on transplanted mesenchymal stem cells in hindlimb ischemic mice via regulation of autophagy. Sci Rep.

[186] Wang L., Zhu Z.M., Zhang N.K. (2016). Apelin: an endogenous peptide essential for cardiomyogenic differentiation of mesenchymal stem cells via activating extracellular signal-regulated kinase 1/2 and 5. Cell Biol Int.

[187] Hou J., Zhong T., Guo T. (2017). Apelin promotes mesenchymal stem cells survival and vascularization under hypoxic-ischemic condition in vitro involving the upregulation of vascular endothelial growth factor. Exp Mol Pathol.

[188] Paik D.T., Tian L., Lee J. (2018). Large-scale single-cell RNA-Seq reveals molecular signatures of heterogeneous populations of human induced pluripotent stem cell-derived endothelial cells. Circ Res.

[189] Gao L.R., Zhang N.K., Zhang Y. (2018). Overexpression of apelin in Wharton’ jelly mesenchymal stem cell reverses insulin resistance and promotes pancreatic beta cell proliferation in type 2 diabetic rats. Stem Cell Res Ther.

[190] Hang K., Ye C., Xu J. (2019). Apelin enhances the osteogenic differentiation of human bone marrow mesenchymal stem cells partly through Wnt/beta-catenin signaling pathway. Stem Cell Res Ther.

[191] Chen Q., Liu Y., Jeong H.W. (2019). Apelin+ endothelial niche cells control hematopoiesis and mediate vascular regeneration after myeloablative injury. Cell Stem Cell.

[192] Qu Y., Feric N., Pallotta I., Singh R., Sobbi R., Vargas H.M. (2020). Inotropic assessment in engineered 3D cardiac tissues using human induced pluripotent stem cell-derived cardiomyocytes in the Biowire(TM) II platform. J Pharmacol Toxicol Methods.

[193] Fu J., Chen X., Liu X. (2020). ELABELA ameliorates hypoxic/ischemic-induced bone mesenchymal stem cell apoptosis via alleviation of mitochondrial dysfunction and activation of PI3K/AKT and ERK1/2 pathways. Stem Cell Res Ther.

[194] Chen L., Shi X., Xie J. (2021). Apelin-13 induces mitophagy in bone marrow mesenchymal stem cells to suppress intracellular oxidative stress and ameliorate osteoporosis by activation of AMPK signaling pathway. Free Radic Biol Med.

[195] Zhang H., Zhao C., Jiang G. (2021). Apelin rejuvenates aged human mesenchymal stem cells by regulating autophagy and improves cardiac protection after infarction. Front Cell Dev Biol.

[196] Jackson M., Fidanza A., Taylor A.H. (2021). Modulation of APLNR signaling is required during the development and maintenance of the hematopoietic system. Stem Cell Rep.

[197] Chen G., Liang X., Han Q. (2022). Apelin-13 pretreatment promotes the cardioprotective effect of mesenchymal stem cells against myocardial infarction by improving their survival. Stem Cells Int.

[198] Liu Q., Zhou S., Wang X. (2022). Apelin alleviated neuroinflammation and promoted endogenous neural stem cell proliferation and differentiation after spinal cord injury in rats. J Neuroinflammation.

[199] Şişli H.B., Şenkal S., Hayal T.B., Bulut E., Doğan A. (2023). Regulatory role of apelin receptor signaling in migration and differentiation of mouse embryonic stem cell-derived mesoderm cells and mesenchymal stem/stromal cells. Hum Cell.

[200] Macrae R.G.C., Colzani M.T., Williams T.L. (2023). Inducible apelin receptor knockdown reduces differentiation efficiency and contractility of hESC-derived cardiomyocytes. Cardiovasc Res.

[201] Li T., Tang Q., Xu J. (2023). Apelin-overexpressing neural stem cells in conjunction with a silk fibroin nanofiber scaffold for the treatment of traumatic brain injury. Stem Cells Dev.

[202] Şişli H.B., Şenkal Turhan S., Bulut E., Şahin F., Doğan A. (2024). The role of Aplnr signaling in the developmental regulation of mesenchymal stem cell differentiation from human pluripotent stem cells. Adv Biol.

[203] Xu D., Fu J., Liu X. (2024). ELABELA-APJ axis enhances mesenchymal stem cell proliferation and migration via the METTL3/PI3K/AKT pathway. Acta Nat.

[204] Karimi Z., Daryabor G., Masjedi F. (2024). Effects of conditioned media derived from human Wharton’s jelly mesenchymal stem cells on diabetic nephropathy and hepatopathy via modulating TGF-beta and apelin signaling pathways in male rats. BMC Endocr Disord.

[205] Gerasimova T., Poberezhniy D., Nenasheva V. (2024). Inflammatory intracellular signaling in neurons is influenced by glial soluble factors in iPSC-based cell model of PARK2-associated Parkinson’s disease. Int J Mol Sci.

[206] Li T., Zhao Y., Cao Z. (2025). Exosomes derived from apelin-pretreated mesenchymal stem cells ameliorate sepsis-induced myocardial dysfunction by alleviating cardiomyocyte pyroptosis via delivery of miR-34a-5p. Int J Nanomedicine.

[207] Quertermous T. (2007). Apelin and its G protein-coupled receptor regulate cardiac development as well as cardiac function. Dev Cell.

[208] Kidoya H., Naito H., Muramatsu F. (2015). APJ regulates parallel alignment of arteries and veins in the skin. Dev Cell.

[209] Southern C., Cook J.M., Neetoo-Isseljee Z. (2013). Screening beta-arrestin recruitment for the identification of natural ligands for orphan G-protein-coupled receptors. J Biomol Screen.

[210] Deshwar A.R., Chng S.C., Ho L., Reversade B., Scott I.C. (2016). The apelin receptor enhances Nodal/TGFbeta signaling to ensure proper cardiac development. eLife.

[211] Matsumoto M., Hidaka K., Akiho H., Tada S., Okada M., Yamaguchi T. (1996). Low stringency hybridization study of the dopamine D4 receptor revealed D4-like mRNA distribution of the orphan seven-transmembrane receptor, APJ, in human brain. Neurosci Lett.

[212] Edinger A.L., Hoffman T.L., Sharron M. (1998). An orphan seven-transmembrane domain receptor expressed widely in the brain functions as a coreceptor for human immunodeficiency virus type 1 and simian immunodeficiency virus. J Virol.

[213] Pope G.R., Roberts E.M., Lolait S.J., O’Carroll A.M. (2012). Central and peripheral apelin receptor distribution in the mouse: species differences with rat. Peptides.

[214] Kleinz M.J., Skepper J.N., Davenport A.P. (2005). Immunocytochemical localisation of the apelin receptor, APJ, to human cardiomyocytes, vascular smooth muscle and endothelial cells. Regul Pept.

[215] Uhlén M., Fagerberg L., Hallström B.M. (2015). Proteomics. Tissue-based map of the human proteome. Science.

[216] Karlsson M., Zhang C., Méar L. (2021). A single-cell type transcriptomics map of human tissues. Sci Adv.

[217] Vinel C., Lukjanenko L., Batut A. (2018). The exerkine apelin reverses age-associated sarcopenia. Nat Med.

[218] Chapman F.A., Maguire J.J., Newby D.E., Davenport A.P., Dhaun N. (2023). Targeting the apelin system for the treatment of cardiovascular diseases. Cardiovasc Res.

[219] Dray C., Debard C., Jager J. (2010). Apelin and APJ regulation in adipose tissue and skeletal muscle of type 2 diabetic mice and humans. Am J Physiol Endocrinol Metab.

[220] Ringström C., Nitert M.D., Bennet H. (2010). Apelin is a novel islet peptide. Regul Pept.

[221] Hall C., Ehrlich L., Venter J. (2017). Inhibition of the apelin/apelin receptor axis decreases cholangiocarcinoma growth. Cancer Lett.

[222] Lee T., Park C.K., Ha S.Y. (2019). Prognostic role of apelin receptor expression in hepatocellular carcinoma treated with curative surgical resection. Anticancer Res.

[223] Harford-Wright E., Andre-Gregoire G., Jacobs K.A. (2017). Pharmacological targeting of apelin impairs glioblastoma growth. Brain.

[224] Williams T.L., Nwokoye P., Kuc R.E. (2024). Expression of the apelin receptor, a novel potential therapeutic target, and its endogenous ligands in diverse stem cell populations in human glioblastoma. Front Neurosci.

[225] Kleinz M.J., Davenport A.P. (2004). Immunocytochemical localization of the endogenous vasoactive peptide apelin to human vascular and endocardial endothelial cells. Regul Pept.

[226] Peltonen T., Näpänkangas J., Vuolteenaho O. (2009). Apelin and its receptor APJ in human aortic valve stenosis. J Heart Valve Dis.

[227] Chen W., Oue T., Ueno T., Uehara S., Usui N., Fukuzawa M. (2013). Apelin is a marker of the progression of liver fibrosis and portal hypertension in patients with biliary atresia. Pediatr Surg Int.

[228] Wang Z., Greeley G.H., Qiu S. (2008). Immunohistochemical localization of apelin in human normal breast and breast carcinoma. J Mol Histol.

[229] Han S., Wang G., Qiu S. (2007). Increased colonic apelin production in rodents with experimental colitis and in humans with IBD. Regul Pept.

[230] Mlyczyńska E., Kurowska P., Drwal E. (2020). Apelin and apelin receptor in human placenta: expression, signalling pathway and regulation of trophoblast JEG-3 and BeWo cells proliferation and cell cycle. Int J Mol Med.

[231] Nyimanu D., Kay R.G., Kuc R.E. (2021). In vitro metabolism of synthetic Elabela/Toddler (ELA-32) peptide in human plasma and kidney homogenates analyzed with mass spectrometry and validation of endogenous peptide quantification in tissues by ELISA. Peptides.

[232] Bassoni D.L., Raab W.J., Achacoso P.L., Loh C.Y., Wehrman T.S. (2012). Measurements of beta-arrestin recruitment to activated seven transmembrane receptors using enzyme complementation. Methods Mol Biol.

[233] Williams T.L., Nyimanu D., Kuc R.E. (2024). The biased apelin receptor agonist, MM07, reverses Sugen/hypoxia-induced pulmonary arterial hypertension as effectively as the endothelin antagonist macitentan. Front Pharmacol.

[234] Dray C., Knauf C., Daviaud D. (2008). Apelin stimulates glucose utilization in normal and obese insulin-resistant mice. Cell Metab.

[235] Kazemi-Bajestani S.M.R., Patel V.B., Wang W., Oudit G.Y. (2012). Targeting the ACE2 and apelin pathways are novel therapies for heart failure: opportunities and challenges. Cardiol Res Pract.

[236] Davenport A.P., Scully C.C.G., de Graaf C., Brown A.J.H., Maguire J.J. (2020). Advances in therapeutic peptides targeting G protein-coupled receptors. Nat Rev Drug Discov.

[237] De Mota N., Lenkei Z., Llorens-Cortès C. (2000). Cloning, pharmacological characterization and brain distribution of the rat apelin receptor. Neuroendocrinology.

[238] Lee D.K., Saldivia V.R., Nguyen T., Cheng R., George S.R., O’Dowd B.F. (2005). Modification of the terminal residue of apelin-13 antagonizes its hypotensive action. Endocrinology.

[239] Trân K., Murza A., Sainsily X. (2018). A systematic exploration of macrocyclization in Apelin-13: impact on binding, signaling, stability, and cardiovascular effects. J Med Chem.

[240] Gerbier R., Alvear-Perez R., Margathe J.F. (2017). Development of original metabolically stable apelin-17 analogs with diuretic and cardiovascular effects. FASEB J.

[241] Girault-Sotias P.E., Deloux R., De Mota N. (2025). The metabolically resistant Apelin-17 analogue LIT01-196 reduces cardiac dysfunction and remodelling in heart failure after myocardial infarction. Can J Cardiol.

[242] Jia Z.Q., Hou L., Leger A. (2012). Cardiovascular effects of a pegylated apelin. Peptides.

[243] Galon-Tilleman H., Yang H., Bednarek M.A. (2017). Apelin-36 modulates blood glucose and body weight independently of canonical APJ receptor signaling. J Biol Chem.

[244] Nyimanu D., Kuc R.E., Williams T.L. (2019). Apelin-36-[L28A] and Apelin-36-[L28C(30kDa-PEG)] peptides that improve diet induced obesity are G protein biased ligands at the apelin receptor. Peptides.

[245] Ren H., Li J., Zhang N. (2020). Function-based high-throughput screening for antibody antagonists and agonists against G protein-coupled receptors. Commun Biol.

[246] Reaux A., De Mota N., Skultetyova I. (2001). Physiological role of a novel neuropeptide, apelin, and its receptor in the rat brain. J Neurochem.

[247] Evans N.A., Groarke D.A., Warrack J. (2001). Visualizing differences in ligand-induced beta-arrestin-GFP interactions and trafficking between three recently characterized G protein-coupled receptors. J Neurochem.

[248] Goudra B. (2022). Oliceridine- opioid of the 21st century. Saudi J Anaesth.

[249] Ceraudo E., Galanth C., Carpentier E. (2014). Biased signaling favoring gi over beta-arrestin promoted by an apelin fragment lacking the C-terminal phenylalanine. J Biol Chem.

[250] Murza A., Sainsily X., Côté J. (2017). Structure-activity relationship of novel macrocyclic biased apelin receptor agonists. Org Biomol Chem.

[251] Théroux L., Van Den Hauwe R., Trân K. (2023). Signaling modulation via minimal C-terminal modifications of Apelin-13. ACS Pharmacol Transl Sci.

[252] Yokoyama Y., Sekiguchi A., Fujiwara C. (2018). Inhibitory regulation of skin fibrosis in systemic sclerosis by Apelin/APJ signaling. Arthritis Rheumatol.

[253] Yang P., Read C., Kuc R.E. (2019). A novel cyclic biased agonist of the apelin receptor, MM07, is disease modifying in the rat monocrotaline model of pulmonary arterial hypertension. Br J Pharmacol.

[254] Read C., Nyimanu D., Yang P. (2021). The G protein biased small molecule apelin agonist CMF-019 is disease modifying in endothelial cell apoptosis in vitro and induces vasodilatation without desensitisation in vivo. Front Pharmacol.

[255] Margathe J.F., Iturrioz X., Alvear-Perez R. (2014). Structure-activity relationship studies toward the discovery of selective apelin receptor agonists. J Med Chem.

[256] Narayanan S., Maitra R., Deschamps J.R. (2016). Discovery of a novel small molecule agonist scaffold for the APJ receptor. Bioorg Med Chem.

[257] Narayanan S., Vasukuttan V., Rajagopal S., Maitra R., Runyon S.P. (2020). Identification of potent pyrazole based APELIN receptor (APJ) agonists. Bioorg Med Chem.

[258] Eisenstein M. (2022). Machine learning powers biobank-driven drug discovery. Nat Biotechnol.

[259] Arnold C. (2024). After obesity drugs’ success, companies rush to preserve skeletal muscle. Nat Biotechnol.

[260] Sabry M.M., Mahmoud M.M., Shoukry H.S., Rashed L., Kamar S.S., Ahmed M.M. (2019). Interactive effects of apelin, renin-angiotensin system and nitric oxide in treatment of obesity-induced type 2 diabetes mellitus in male albino rats. Arch Physiol Biochem.

[261] Hachtel S., Wohlfart P., Weston J., Müller M. (2014). Benzoimidazole-carboxylic acid amide derivatives as APJ receptor modulators. International patent application WO2014044738. https://patents.google.com/patent/WO2014044738A1/en.

[262] Anto S., Sun C., O’Rourke S.T. (2025). Activation of APJ receptors by CMF-019, but not apelin, causes endothelium-dependent relaxation of spontaneously hypertensive rat coronary arteries. J Cardiovasc Pharmacol.

[263] Lim S., Shi S., Zhang J., Parsley E. (2023). First-in-Human Single/Multiple Ascending Dose Study of ANPA-0073, a Novel Small Molecule G-protein Biased Apelin Receptor Agonist, in Healthy Volunteers. http://SRTX2303C_ATS_ANPA_SAD_MAD_Lim_ePOSTER_0523_FIN-3%5f7.pdf.

[264] Tsyben A. (2023).

[265] Yang P., Maguire J.J., Davenport A.P. (2015). Apelin, Elabela/Toddler, and biased agonists as novel therapeutic agents in the cardiovascular system. Trends Pharmacol Sci.

[266] Kuba K., Sato T., Imai Y., Yamaguchi T. (2019). Apelin and Elabela/Toddler; double ligands for APJ/Apelin receptor in heart development, physiology, and pathology. Peptides.

[267] Kim J., Kang Y., Kojima Y. (2013). An endothelial apelin-FGF link mediated by miR-424 and miR-503 is disrupted in pulmonary arterial hypertension. Nat Med.

[268] Alastalo T.P., Li M., Perez J. (2011). Disruption of PPARγ/β-catenin-mediated regulation of apelin impairs BMP-induced mouse and human pulmonary arterial EC survival. J Clin Invest.

[269] Hu Y., Zong Y., Jin L., Zou J., Wang Z. (2023). Reduced Apela/APJ system expression in patients with pulmonary artery hypertension secondary to chronic obstructive pulmonary disease. Heart Lung.

[270] Owen N.E., Nyimanu D., Kuc R.E. (2021). Plasma levels of apelin are reduced in patients with liver fibrosis and cirrhosis but are not correlated with circulating levels of bone morphogenetic protein 9 and 10. Peptides.

[271] Niemczyk A., Waśkiel-Burnat A., Zaremba M., Czuwara J., Rudnicka L. (2023). The profile of adipokines associated with fibrosis and impaired microcirculation in systemic sclerosis. Adv Med Sci.

[272] Csósza G., Szűcs G., Rozgonyi Z. (2023). Circulating apelin, IL22RA2 and VEGF in pre-capillary pulmonary hypertension. Physiol Int.

[273] Zhou Y., Tabib T., Huang M. (2024). Molecular changes implicate angiogenesis and arterial remodeling in systemic sclerosis-associated and idiopathic pulmonary hypertension. Arterioscler Thromb Vasc Biol.

[274] Gillich A., Zhang F., Farmer C.G. (2020). Capillary cell-type specialization in the alveolus. Nature.

[275] Falcão-Pires I., Gonçalves N., Henriques-Coelho T., Moreira-Gonçalves D., Roncon-Albuquerque R., Leite-Moreira A.F. (2009). Apelin decreases myocardial injury and improves right ventricular function in monocrotaline-induced pulmonary hypertension. Am J Physiol Heart Circ Physiol.

[276] Drake J.I., Bogaard H.J., Mizuno S. (2011). Molecular signature of a right heart failure program in chronic severe pulmonary hypertension. Am J Respir Cell Mol Biol.

[277] Frump A.L., Goss K.N., Vayl A. (2015). Estradiol improves right ventricular function in rats with severe angioproliferative pulmonary hypertension: effects of endogenous and exogenous sex hormones. Am J Physiol Lung Cell Mol Physiol.

[278] Neto-Neves E.M., Frump A.L., Vayl A., Kline J.A., Lahm T. (2017). Isolated heart model demonstrates evidence of contractile and diastolic dysfunction in right ventricles from rats with sugen/hypoxia-induced pulmonary hypertension. Physiol Rep.

[279] Tu L., Desroches-Castan A., Mallet C. (2019). Selective BMP-9 inhibition partially protects against experimental pulmonary hypertension. Circ Res.

[280] Frump A.L., Albrecht M., Yakubov B. (2021). 17β-Estradiol and estrogen receptor α protect right ventricular function in pulmonary hypertension via BMPR2 and apelin. J Clin Invest.

[281] Hennigs J.K., Cao A., Li C.G. (2021). PPARgamma-p53-mediated Vasculoregenerative program to reverse pulmonary hypertension. Circ Res.

[282] Bertero T., Lu Y., Annis S. (2014). Systems-level regulation of microRNA networks by miR-130/301 promotes pulmonary hypertension. J Clin Invest.

[283] Fan J., Fan X., Guang H. (2020). Upregulation of miR-335-3p by NF-κB transcriptional regulation contributes to the induction of pulmonary arterial hypertension via APJ during hypoxia. Int J Biol Sci.

[284] Andersen C.U., Markvardsen L.H., Hilberg O., Simonsen U. (2009). Pulmonary apelin levels and effects in rats with hypoxic pulmonary hypertension. Respir Med.

[285] Hu Y., Jin L., Pan Y., Zou J., Wang Z. (2022). Apela gene therapy alleviates pulmonary hypertension in rats. FASEB J.

[286] Wang L., Wang W., Wu T., Chen L., Zong G. (2025). Mesenchymal stem cell-based apelin gene therapy improves pulmonary artery remodeling in monocrotaline-induced pulmonary hypertension through PI3K/AKT/eNOS and ERK1/2 signaling pathways. J Cardiovasc Transl Res.

[287] Spiekerkoetter E., Tian X., Cai J. (2013). FK506 activates BMPR2, rescues endothelial dysfunction, and reverses pulmonary hypertension. J Clin Invest.

[288] Nickel N.P., Spiekerkoetter E., Gu M. (2015). Elafin reverses pulmonary hypertension via Caveolin-1-Dependent bone morphogenetic protein signaling. Am J Respir Crit Care Med.

[289] Chong K.S., Gardner R.S., Morton J.J., Ashley E.A., McDonagh T.A. (2006). Plasma concentrations of the novel peptide apelin are decreased in patients with chronic heart failure. Eur J Heart Fail.

[290] Iwanaga Y., Kihara Y., Takenaka H., Kita T. (2006). Down-regulation of cardiac apelin system in hypertrophied and failing hearts: Possible role of angiotensin II-angiotensin type 1 receptor system. J Mol Cell Cardiol.

[291] Koguchi W., Kobayashi N., Takeshima H., Ishikawa M., Sugiyama F., Ishimitsu T. (2012). Cardioprotective effect of apelin-13 on cardiac performance and remodeling in end-stage heart failure. Circ J.

[292] Hamada J., Baasanjav A., Ono N. (2015). Possible involvement of downregulation of the apelin-APJ system in doxorubicin-induced cardiotoxicity. Am J Physiol Heart Circ Physiol.

[293] Wang W., McKinnie S.M.K., Patel V.B. (2013). Loss of Apelin exacerbates myocardial infarction adverse remodeling and ischemia-reperfusion injury: therapeutic potential of synthetic Apelin analogues. J Am Heart Assoc.

[294] Han L., Jie B., Luo J. (2020). Increased plasma level of apelin with NYHA grade II and III but not IV. Amino Acids.

[295] Szczurek W., Gasior M., Skrzypek M., Szyguła-Jurkiewicz B. (2020). Apelin improves prognostic value of HFSS (Heart Failure Survival Score) and MAGGIC (meta-analysis global group in chronic heart failure) scales in ambulatory patients with end-stage heart failure. J Clin Med.

[296] Berezin A.A., Fushtey I.M., Berezin A.E. (2022). Discriminative utility of apelin-to-NT-pro-brain natriuretic peptide ratio for heart failure with preserved ejection fraction among type 2 diabetes mellitus patients. J Cardiovasc Dev Dis.

[297] Boal F., Roumegoux J., Alfarano C. (2015). Apelin regulates FoxO3 translocation to mediate cardioprotective responses to myocardial injury and obesity. Sci Rep.

[298] Jia Y.X., Pan C.S., Zhang J. (2006). Apelin protects myocardial injury induced by isoproterenol in rats. Regul Pept.

[299] Berry M.F., Pirolli T.J., Jayasankar V. (2004). Apelin has in vivo inotropic effects on normal and failing hearts. Circulation.

[300] Wang W., Shen M., Fischer C. (2019). Apelin protects against abdominal aortic aneurysm and the therapeutic role of neutral endopeptidase resistant apelin analogs. Proc Natl Acad Sci U S A.

[301] Sidorova M., Studneva I., Bushuev V. (2020). [MeArg^1^, NLe^10^]-apelin-12: optimization of solid-phase synthesis and evaluation of biological properties in vitro and in vivo. Peptides.

[302] Serpooshan V., Sivanesan S., Huang X. (2015). [Pyr1]-Apelin-13 delivery via nano-liposomal encapsulation attenuates pressure overload-induced cardiac dysfunction. Biomaterials.

[303] Reed A.B., Lanman B.A., Holder J.R. (2020). Half-life extension of peptidic APJ agonists by N-terminal lipid conjugation. Bioorg Med Chem Lett.

[304] Tang L., Qiu H., Xu B. (2024). Microparticle-mediated delivery of apelin improves heart function in post myocardial infarction mice. Circ Res.

[305] Lv W., Zhang L., Cheng X. (2020). Apelin inhibits angiotensin ii-induced atrial fibrosis and atrial fibrillation via TGF-β1/Smad2/α-SMA pathway. Front Physiol.

[306] Zeng H., He X., Chen J.X. (2020). Endothelial sirtuin 3 dictates glucose transport to cardiomyocyte and sensitizes pressure overload-induced heart failure. J Am Heart Assoc.

[307] Ni T., Lin N., Huang X. (2020). Icariin ameliorates diabetic cardiomyopathy through Apelin/Sirt3 signalling to improve mitochondrial dysfunction. Front Pharmacol.

[308] Gaddam R.R., Kim Y.R., Jacobs J.S. (2022). The microRNA-204-5p inhibits APJ signalling and confers resistance to cardiac hypertrophy and dysfunction. Clin Transl Med.

[309] Rong R., Yuan T., Yan Z. (2025). LncRNA NAV2-AS2 is critical for fibroblast-to-myofibroblast transition and cardiac fibrosis. Int J Biol Macromol.

[310] Pan Y., Li Q., Yan H., Huang J., Wang Z. (2020). Apela improves cardiac and renal function in mice with acute myocardial infarction. J Cell Mol Med.

[311] Sainsily X., Coquerel D., Giguere H. (2021). Elabela protects spontaneously hypertensive rats from hypertension and cardiorenal dysfunctions exacerbated by dietary high-salt intake. Front Pharmacol.

[312] Xi Y., Yu D., Yang R. (2019). Recombinant Fc-Elabela fusion protein has extended plasma half-life andmitigates post-infarct heart dysfunction in rats. Int J Cardiol.

[313] Wang X., Zhang L., Feng M., Xu Z., Cheng Z., Qian L. (2022). ELA-11 protects the heart against oxidative stress injury induced apoptosis through ERK/MAPK and PI3K/AKT signaling pathways. Front Pharmacol.

[314] Zhang Z., Tang J., Song J. (2022). Elabela alleviates ferroptosis, myocardial remodeling, fibrosis and heart dysfunction in hypertensive mice by modulating the IL-6/STAT3/GPX4 signaling. Free Radic Biol Med.

[315] Zhang T., Wang X., Wang Z. (2023). Canagliflozin ameliorates ventricular remodeling through Apelin/angiotensin-converting enzyme 2 signaling in heart failure with preserved ejection fraction rats. Pharmacology.

[316] Berezin A.A., Fushtey I.M., Berezin A.E. (2022). The effect of SGLT2 inhibitor Dapagliflozin on serum levels of apelin in T2DM patients with heart failure. Biomedicines.

[317] Javaheri A., Diab A., Zhao L. (2022). Proteomic analysis of effects of spironolactone in heart failure with preserved ejection fraction. Circ Heart Fail.

[318] Su J., Hu Y., Cheng J. (2023). Comprehensive analysis of the RNA transcriptome expression profiles and construction of the ceRNA network in heart failure patients with sacubitril/valsartan therapeutic heterogeneity after acute myocardial infarction. Eur J Pharmacol.

[319] Wu Y., Cui C., Bi F.F. (2022). Montelukast, cysteinyl leukotriene receptor 1 antagonist, inhibits cardiac fibrosis by activating APJ. Eur J Pharmacol.

[320] Parikh M., Shah S., Basu R. (2022). Transcriptomic signatures of end-stage human dilated cardiomyopathy hearts with and without left ventricular assist device support. Int J Mol Sci.

[321] Lin R., Rahtu-Korpela L., Szabo Z. (2022). MiR-185-5p regulates the development of myocardial fibrosis. J Mol Cell Cardiol.

[322] Sorhede Winzell M., Magnusson C., Ahrén B. (2005). The apj receptor is expressed in pancreatic islets and its ligand, apelin, inhibits insulin secretion in mice. Regul Pept.

[323] Guo L., Li Q., Wang W. (2009). Apelin inhibits insulin secretion in pancreatic beta-cells by activation of PI3-kinase-phosphodiesterase 3B. Endocr Res.

[324] Li L., Yang G., Li Q. (2006). Changes and relations of circulating visfatin, apelin, and resistin levels in normal, impaired glucose tolerance, and type 2 diabetic subjects. Exp Clin Endocrinol Diabetes.

[325] Han S., Englander E.W., Gomez G.A. (2015). Pancreatic islet APJ deletion reduces islet density and glucose tolerance in mice. Endocrinology.

[326] Attane C., Foussal C., Le Gonidec S. (2012). Apelin treatment increases complete fatty acid oxidation, mitochondrial oxidative capacity, and biogenesis in muscle of insulin-resistant mice. Diabetes.

[327] Castan-Laurell I., El Boustany R., Pereira O. (2020). Plasma apelin and risk of type 2 diabetes in a cohort from the community. Diabetes Care.

[328] Yin C., Zhang H., Zhang M., Xiao Y. (2020). Adropin and apelin-12 efficiently predict metabolic syndrome in obese children. Pediatr Diabetes.

[329] Zheng X.D., Huang Y., Li H. (2021). Regulatory role of Apelin-13-mediated PI3K/AKT signaling pathway in the glucose and lipid metabolism of mouse with gestational diabetes mellitus. Immunobiology.

[330] Guo Y.Y., Li T., Liu H. (2020). Circulating levels of Elabela and Apelin in the second and third trimesters of pregnancies with gestational diabetes mellitus. Gynecol Endocrinol.

[331] Yuzbashian E., Asghari G., Aghayan M. (2019). Dietary glycemic index and dietary glycemic load is associated with apelin gene expression in visceral and subcutaneous adipose tissues of adults. Nutr Metab.

[332] Ranjbar Kohan N., Tabandeh M.R., Nazifi S., Soleimani Z. (2020). L-carnitine improves metabolic disorders and regulates apelin and apelin receptor genes expression in adipose tissue in diabetic rats. Physiol Rep.

[333] Chen K., Ou B., Huang Q., Deng D., Xiang Y., Hu F. (2024). LncRNA NEAT1 aggravates human microvascular endothelial cell injury by inhibiting the Apelin/Nrf2/HO-1 signalling pathway in type 2 diabetes mellitus with obstructive sleep apnoea. Epigenetics.

[334] He S., Li J., Wang J., Zhang Y. (2019). Hypoxia exposure alleviates impaired muscular metabolism, glucose tolerance, and aerobic capacity in apelin-knockout mice. FEBS Open Bio.

[335] Gao Z., Zhong X., Tan Y.X., Liu D. (2021). Apelin-13 alleviates diabetic nephropathy by enhancing nitric oxide production and suppressing kidney tissue fibrosis. Int J Mol Med.

[336] Huang M., Chang J., Liu Y., Yin J., Zeng X. (2025). Apelin/APJ alleviates diabetic nephropathy by improving glomerular endothelial cells dysfunction via SIRT3-KLF15. Mol Med Rep.

[337] Huang M., Chang J., Liu Y., Yin J., Zeng X. (2025). Apelin/APJ increased renal blood flow through endothelial BKCa channel induced p-eNOS and ET-1 in diabetic conditions. Peptides.

[338] Zhong X., Zhang J. (2024). RUNX3-activated apelin signaling inhibits cell proliferation and fibrosis in diabetic nephropathy by regulation of the SIRT1/FOXO pathway. Diabetol Metab Syndr.

[339] Wang Q., Liu X., Zhai A., Xu H., Ma S., Liu Y. (2024). Expression of apelin-13 and its negative correlation with TGF-beta1 in patients with diabetic kidney disease. Exp Ther Med.

[340] Guo C., Liu Y., Zhao W. (2015). Apelin promotes diabetic nephropathy by inducing podocyte dysfunction via inhibiting proteasome activities. J Cell Mol Med.

[341] Zheng X., Yin L., Song J. (2023). ELABELA protects against diabetic kidney disease by activating high glucose-inhibited renal tubular autophagy. J Biomed Res.

[342] Chen Z., Wang Z., Hu Y. (2023). ELABELA/APJ axis prevents diabetic glomerular endothelial injury by regulating AMPK/NLRP3 pathway. Inflammation.

[343] Wu L., Chen L., Li L.L. (2017). Apelin/APJ system: a novel promising therapy target for pathological angiogenesis. Clin Chim Acta.

[344] Feng J., Yang W., Luan F. (2022). The protective role of apelin in the early stages of diabetic retinopathy. Int J Mol Sci.

[345] Yasir M., Senthilkumar G.P., Jayashree K. (2022). Ramesh Babu K, Vadivelan M, Palanivel C. Association of serum omentin-1, apelin and chemerin concentrations with the presence and severity of diabetic retinopathy in type 2 diabetes mellitus patients. Arch Physiol Biochem.

[346] Li Y., Hu Q., Wang B. (2023). Effects of Apelin on the fibrosis of retinal tissues and Muller cells in diabetes retinopathy through the JAK2/STAT3 signalling pathway. Autoimmunity.

[347] Robillard S., Trân K., Lachance M.S. (2023). Apelin prevents diabetes-induced poor collateral vessel formation and blood flow reperfusion in ischemic limb. Front Cardiovasc Med.

[348] Zhang P., Wang A.P., Yang H.P. (2020). Apelin-13 attenuates high glucose-induced calcification of MOVAS cells by regulating MAPKs and PI3K/AKT pathways and ROS-mediated signals. Biomed Pharmacother.

[349] Feng J., Zhao H., Du M., Wu X. (2019). The effect of apelin-13 on pancreatic islet beta cell mass and myocardial fatty acid and glucose metabolism of experimental type 2 diabetic rats. Peptides.

[350] An S., Wang X., Shi H. (2020). Apelin protects against ischemia-reperfusion injury in diabetic myocardium via inhibiting apoptosis and oxidative stress through PI3K and p38-MAPK signaling pathways. Aging.

[351] Li B., Yin J., Chang J. (2021). Apelin/APJ relieve diabetic cardiomyopathy by reducing microvascular dysfunction. J Endocrinol.

[352] Bielska A., Niemira M., Bauer W. (2022). Serum miRNA profile in diabetic patients with ischemic heart disease as a promising non-invasive biomarker. Front Endocrinol.

[353] Li C., Miao X., Wang S. (2021). Elabela may regulate SIRT3-mediated inhibition of oxidative stress through Foxo3a deacetylation preventing diabetic-induced myocardial injury. J Cell Mol Med.

[354] Hu S., Lan C., Shu S., Wang L. (2025). Apelin-13 alleviates diabetes-associated cognitive decline by reducing oxidative stress and mitochondrial dysfunction via the SIRT3/Foxo3 pathway. Biotechnol Appl Biochem.

[355] Jiang Y., Wang S., Liu X. (2022). Low serum apelin levels are associated with mild cognitive impairment in Type 2 diabetic patients. BMC Endocr Disord.

[356] Das M., Annie L., Derkach K.V., Shpakov A.O., Gurusubramanian G., Roy V.K. (2021). Expression and localization of apelin and its receptor in the testes of diabetic mice and its possible role in steroidogenesis. Cytokine.

[357] Song K., Yang X., An G. (2022). Targeting APLN/APJ restores blood-testis barrier and improves spermatogenesis in murine and human diabetic models. Nat Commun.

[358] Mehri K., Hamidian G., Babri S., Farajdokht F., Zavvari Oskuye Z. (2024). Exercise and insulin glargine administration in mothers with diabetes during pregnancy ameliorate function of testis in offspring: consequences on apelin-13 and its receptor. Life Sci.

[359] Hong Y., Li J., Zhong Y. (2023). Elabela inhibits TRAF1/NF-κB induced oxidative DNA damage to promote diabetic foot ulcer wound healing. iScience.

[360] O'Harte F.P.M., Parthsarathy V., Flatt P.R. (2020). Chronic apelin analogue administration is more effective than established incretin therapies for alleviating metabolic dysfunction in diabetic db/db mice. Mol Cell Endocrinol.

[361] Tanday N., Irwin N., Moffett R.C., Flatt P.R., O’Harte F.P.M. (2020). Beneficial actions of a long-acting apelin analogue in diabetes are related to positive effects on islet cell turnover and transdifferentiation. Diabetes Obes Metab.

[362] Cui J., Wang M., Zhang W. (2024). Enhancing insulin sensitivity in type 2 diabetes mellitus using apelin-loaded small extracellular vesicles from Wharton’s jelly-derived mesenchymal stem cells: a novel therapeutic approach. Diabetol Metab Syndr.

[363] Hus-Citharel A., Bouby N., Frugière A., Bodineau L., Gasc J.M., Llorens-Cortes C. (2008). Effect of apelin on glomerular hemodynamic function in the rat kidney. Kidney Int.

[364] Reaux-Le Goazigo A., Morinville A., Burlet A., Llorens-Cortes C., Beaudet A. (2004). Dehydration-induced cross-regulation of apelin and vasopressin immunoreactivity levels in magnocellular hypothalamic neurons. Endocrinology.

[365] Boulkeroua C., Ayari H., Khalfaoui T. (2019). Apelin-13 regulates vasopressin-induced Aquaporin-2 expression and trafficking in kidney collecting duct cells. Cell Physiol Biochem.

[366] Hus-Citharel A., Bodineau L., Frugière A., Joubert F., Bouby N., Llorens-Cortes C. (2014). Apelin counteracts vasopressin-induced water reabsorption via cross talk between apelin and vasopressin receptor signaling pathways in the rat collecting duct. Endocrinology.

[367] Ayari H. (2022). Chraibi A Apelin-13 decreases epithelial sodium channel (ENaC) expression and activity in kidney collecting duct cells. Cell Physiol Biochem.

[368] Azizi M., Iturrioz X., Blanchard A. (2008). Reciprocal regulation of plasma apelin and vasopressin by osmotic stimuli. J Am Soc Nephrol.

[369] Chen H., Wan D., Wang L. (2015). Apelin protects against acute renal injury by inhibiting TGF-beta1. Biochim Biophys Acta.

[370] Chen H., Wang L., Wang W. (2017). ELABELA and an ELABELA fragment protect against AKI. J Am Soc Nephrol.

[371] Gholampour F., Bagheri A., Barati A., Masoudi R., Owji S.M. (2020). Remote ischemic Perconditioning modulates apelin expression after renal ischemia-reperfusion injury. J Surg Res.

[372] Lopes-Goncalves G., Costa-Pessoa J.M., de Ponte M.C., Braz H.M., Oliveira-Souza M. (2025). Insights into the effects of apelin-13 on renal function and NHE3 activity following ischemia/reperfusion-induced acute kidney injury. Front Physiol.

[373] Kim J.S., Yang J.W., Han B.G., Kwon H.J., Kim J.H., Choi S.O. (2017). Protective role of Apelin Against cyclosporine-induced renal tubular injury in rats. Transplant Proc.

[374] Sagiroglu T., Torun N., Yagci M., Yalta T., Sagiroglu G., Oguz S. (2012). Effects of apelin and leptin on renal functions following renal ischemia/reperfusion: an experimental study. Exp Ther Med.

[375] Zheng X., Chen D., Wu J. (2025). Apelin-13 inhibits ischemia-reperfusion mediated podocyte apoptosis by reducing m-TOR phosphorylation to enhance autophagy. FASEB J.

[376] Xu F., Wu M., Lu X. (2022). Effect of Fc-Elabela-21 on renal ischemia/reperfusion injury in mice: mediation of anti-apoptotic effect via Akt phosphorylation. Peptides.

[377] Foreman K.J., Marquez N., Dolgert A. (2018). Forecasting life expectancy, years of life lost, and all-cause and cause-specific mortality for 250 causes of death: reference and alternative scenarios for 2016–40 for 195 countries and territories. Lancet.

[378] Awdishu L., Maxson R., Gratt C., Rubenzik T., Battistella M. (2025). KDIGO 2024 clinical practice guideline on evaluation and management of chronic kidney disease: A primer on what pharmacists need to know. Am J Health Syst Pharm.

[379] Khan M.S., Rashid A.M., Shafi T., Rangaswami J., Cherney D.Z.I., Butler J. (2025). Residual risk of adverse kidney and cardiovascular outcomes in patients with CKD. Clin J Am Soc Nephrol.

[380] Chapman F.A., Nyimanu D., Maguire J.J., Davenport A.P., Newby D.E., Dhaun N. (2021). The therapeutic potential of apelin in kidney disease. Nat Rev Nephrol.

[381] Chen H., Li J., Jiao L. (2014). Apelin inhibits the development of diabetic nephropathy by regulating histone acetylation in Akita mouse. J Physiol.

[382] Day R.T., Cavaglieri R.C., Feliers D. (2013). Apelin retards the progression of diabetic nephropathy. Am J Physiol Renal Physiol.

[383] Muller T., Kalea A.Z., Marquez A. (2018). Apelinergic system in the kidney: implications for diabetic kidney disease. Physiol Rep.

[384] Yin J., Wang Y., Chang J. (2018). Apelin inhibited epithelial-mesenchymal transition of podocytes in diabetic mice through downregulating immunoproteasome subunits β5i. Cell Death Dis.

[385] Zhang Y., Wang Y., Luo M. (2019). Elabela protects against podocyte injury in mice with streptozocin-induced diabetes by associating with the PI3K/Akt/mTOR pathway. Peptides.

[386] Liu Y., Zhang J., Wang Y., Zeng X. (2017). Apelin involved in progression of diabetic nephropathy by inhibiting autophagy in podocytes. Cell Death Dis.

[387] Zhang B.H., Wang W., Wang H., Yin J., Zeng X.J. (2013). Promoting effects of the adipokine, apelin, on diabetic nephropathy. PLoS One.

[388] Zhang J., Yin J., Wang Y., Li B., Zeng X. (2018). Apelin impairs myogenic response to induce diabetic nephropathy in mice. FASEB J.

[389] Wang L.Y., Diao Z.L., Zhang D.L. (2014). The regulatory peptide apelin: a novel inhibitor of renal interstitial fibrosis. Amino Acids.

[390] Wang L.Y., Diao Z.L., Zheng J.F., Wu Y.R., Zhang Q.D., Liu W.H. (2017). Apelin attenuates TGF-beta1-induced epithelial to mesenchymal transition via activation of PKC-epsilon in human renal tubular epithelial cells. Peptides.

[391] Gao R., Wu Y., Yang Q. (2023). The interaction of apelin and FGFR1 ameliorated the kidney fibrosis through suppression of TGFbeta-induced endothelial-to-mesenchymal transition. Oxid Med Cell Longev.

[392] Xu C., Wang F., Chen Y. (2020). ELABELA antagonizes intrarenal renin-angiotensin system to lower blood pressure and protects against renal injury. Am J Physiol Renal Physiol.

[393] Nishida M., Okumura Y., Oka T. (2012). The role of apelin on the alleviative effect of angiotensin receptor blocker in unilateral ureteral obstruction-induced renal fibrosis. Nephron Extra.

[394] Schreiber C.A., Holditch S.J., Generous A., Ikeda Y. (2017). Sustained ELABELA gene therapy in high-salt diet-induced hypertensive rats. Curr Gene Ther.

[395] ‘Chen Z., Wu C., Liu Y. (2020). Correction: ELABELA attenuates deoxycorticosterone acetate/salt-induced hypertension and renal injury by inhibition of NADPH oxidase/ROS/NLRP3 inflammasome pathway. Cell Death Dis.

[396] Blanchard A., Steichen O., De Mota N. (2013). An abnormal apelin/vasopressin balance may contribute to water retention in patients with the syndrome of inappropriate antidiuretic hormone (SIADH) and heart failure. J Clin Endocrinol Metab.

[397] Urwyler S.A., Timper K., Fenske W. (2016). Plasma apelin concentrations in patients with polyuria-polydipsia syndrome. J Clin Endocrinol Metab.

[398] Bizzozero C.A., Monnerat S., Chapman F.A., Dhaun N., Refardt J., Christ-Crain M. (2024). Apelin levels in patients with polyuria-polydipsia syndrome upon copeptin stimulation tests. Eur J Endocrinol.

[399] Chen J., Chen M., Liu Y. (2025). Effects of apelin on age-associated neovascularization impairment in peripheral artery disease. Life Sci.

[400] Kim J., Choi Y.S., Kim J. (2025). Targeted delivery of apelin using a novel extracellular vesicle platform for pulmonary arterial hypertension treatment. Biomaterials.

[401] Markham A. (2020). Oliceridine: first approval. Drugs.

